# Mapping Condition-Dependent Regulation of Lipid Metabolism in *Saccharomyces cerevisiae*

**DOI:** 10.1534/g3.113.006601

**Published:** 2013-11-01

**Authors:** Michael C. Jewett, Christopher T. Workman, Intawat Nookaew, Francisco A. Pizarro, Eduardo Agosin, Lars I. Hellgren, Jens Nielsen

**Affiliations:** * Center for Microbial Biotechnology, DTU Systems Biology, Technical University of Denmark, DK-2800 Kongens Lyngby, Denmark; † Department of Chemical and Biological Engineering and Chemistry of Life Processes Institute Northwestern University, Evanston, Illinois 60208; ‡ Center for Biological Sequence Analysis, DTU Systems Biology, Technical University of Denmark, DK-2800 Kongens Lyngby, Denmark; § Department of Chemical and Biological Engineering, Chalmers University of Technology, SE-412 96 Göteborg, Sweden; ** Faculty of Engineering, King Mongkut’s University of Technology Thonburi, Bangkok 10140, Thailand; †† Department of Chemical and Bioprocess Engineering, School of Engineering, Pontificia Universidad Católica de Chile, Macul, Santiago 8320000, Chile

**Keywords:** integrated systems biology, lipid metabolism, regulation, metabolome, omics

## Abstract

Lipids play a central role in cellular function as constituents of membranes, as signaling molecules, and as storage materials. Although much is known about the role of lipids in regulating specific steps of metabolism, comprehensive studies integrating genome-wide expression data, metabolite levels, and lipid levels are currently lacking. Here, we map condition-dependent regulation controlling lipid metabolism in *Saccharomyces cerevisiae* by measuring 5636 mRNAs, 50 metabolites, 97 lipids, and 57 ^13^C-reaction fluxes in yeast using a three-factor full-factorial design. Correlation analysis across eight environmental conditions revealed 2279 gene expression level-metabolite/lipid relationships that characterize the extent of transcriptional regulation in lipid metabolism relative to major metabolic hubs within the cell. To query this network, we developed integrative methods for correlation of multi-omics datasets that elucidate global regulatory signatures. Our data highlight many characterized regulators of lipid metabolism and reveal that sterols are regulated more at the transcriptional level than are amino acids. Beyond providing insights into the systems-level organization of lipid metabolism, we anticipate that our dataset and approach can join an emerging number of studies to be widely used for interrogating cellular systems through the combination of mathematical modeling and experimental biology.

Lipid metabolism in yeast comprises more than 300 chemical reactions and 100 chemical species (Nookaew *et al.* 2008). These chemical species include fatty acids, sterols, steryl esters, acylglycerols, phospholipids, and sphingolipids. Despite a wide range of chemical and structural diversity, all lipid species share a common carbon precursor, namely acetyl-CoA, and only a few additional molecules, which include, for example, dihydroxyacetone phosphate, glucose-6-phosphate, serine, and S-adenosyl methionine, are needed to synthesize almost all lipids. With so few precursors needed to synthesize such a large number of diverse lipid species, a high level of genetic and cellular regulation is required to ensure homeostasis in lipid composition. It is well-known that lipid metabolism is tightly regulated, particularly at the level of transcription (Nielsen 2009). For example, the transcriptional regulation of sterol homeostasis by Upc2p and Ecm22p (sterol regulatory element-binding protein in humans) uses a feedback pathway to concomitantly control synthesis and uptake (Vik and Rine 2001). As another example, regulation of *INO1* is well-established and serves as a model for studying transcriptional control in general (Chen *et al.* 2007).

Elucidating global regulatory modules in lipid metabolism and how they interact in response to environmental conditions has direct implications for human health and biotechnology (Smith *et al.* 2007; Brehme and Vidal 2010; Gallego *et al.* 2010). In humans, disorders in lipid metabolism, transport, and trafficking are involved in the etiology of many diseases, such as atherosclerosis, diabetes, and cancer (Henneberry and Sturley 2005; Maxfield and Tabas 2005; Hannun and Obeid 2008). Thus, understanding how lipid metabolism is regulated could lead to identification of new therapeutic strategies for treatment of some of these diseases. In biotechnology, efforts to engineer lipid metabolism in microbes offer a promising route toward the production of biodiesel (Steen *et al.* 2010) and dietary supplements or food ingredients (Ruenwai *et al.* 2010; Ruenwai *et al.* 2011; Tavares *et al.* 2011). Hence, mechanistic insights into cellular regulation could help to identify novel metabolic engineering targets, and thus the development of efficient cell factories for the production of these lipids. Despite these interests in understanding regulation of lipid metabolism, comprehensive studies aimed at bridging the gap between transcriptional state and metabolic phenotype are lacking.

Although lacking in lipid metabolism, integrated systems biology studies have been important in gaining a quantitative understanding of complex biological systems (Sauer *et al.* 2007; Yamada and Bork 2009). For example, such studies have revealed DNA damage response pathways (Workman *et al.* 2006), the systemic impact of growth rate (Regenberg *et al.* 2006; Castrillo *et al.* 2007; Fazio *et al.* 2008), the role of Snf1p as a global energy regulator (Usaite *et al.* 2009), the interactions between Snf1p and TORC1 (Zhang *et al.* 2011), novel biosynthetic control mechanisms in amino acid metabolism (Moxley *et al.* 2009), physiological differences between laboratory and wine yeasts (Pizarro *et al.* 2008), the functional landscape of a genome-reduced bacterium (Guell *et al.* 2009; Kuhner *et al.* 2009; Ochman and Raghavan 2009; Yus *et al.* 2009), strategies metabolic networks use to achieve robust operation (Ishii *et al.* 2007), the key factors involved in programmed fruit ripening and tomato development (Rohrmann *et al.* 2011), a model of sphingolipid metabolism (Gupta *et al.* 2011), metabolic pathways involved in environmental adaptation (Gianoulis *et al.* 2009), potential gene–metabolite interactions (Bradley *et al.* 2009), and general design principles controlling gene expression levels/enzyme abundance levels (Fendt *et al.* 2010), among others. The wealth of information generated from these studies emphasizes the importance of integrating data across multiple levels of the cell (mRNAs, proteins, metabolites, fluxes, and others) and protein interaction information (Feist and Palsson 2008; Yamada and Bork 2009; Heinemann and Sauer 2011).

In this study, we set out to elucidate global regulatory structure controlling lipid metabolism under different environmental conditions (Figure 1). Toward this goal, we created a model of lipid metabolism that allows for the integration of mRNA, metabolite, lipid, and flux data with known networks of protein-DNA interactions and metabolic reaction stoichiometry. We present the results of our measurements together with integrative methods for analyzing high-throughput experimental datasets. The condition specificity of the measured data provides the possibility to infer some insights into the global regulatory architecture of lipid metabolism (*i.e.*, regulation structure that is general and can be applied over different environmental perturbations) and into how fluxes toward different lipid species are controlled.

**Figure 1:**
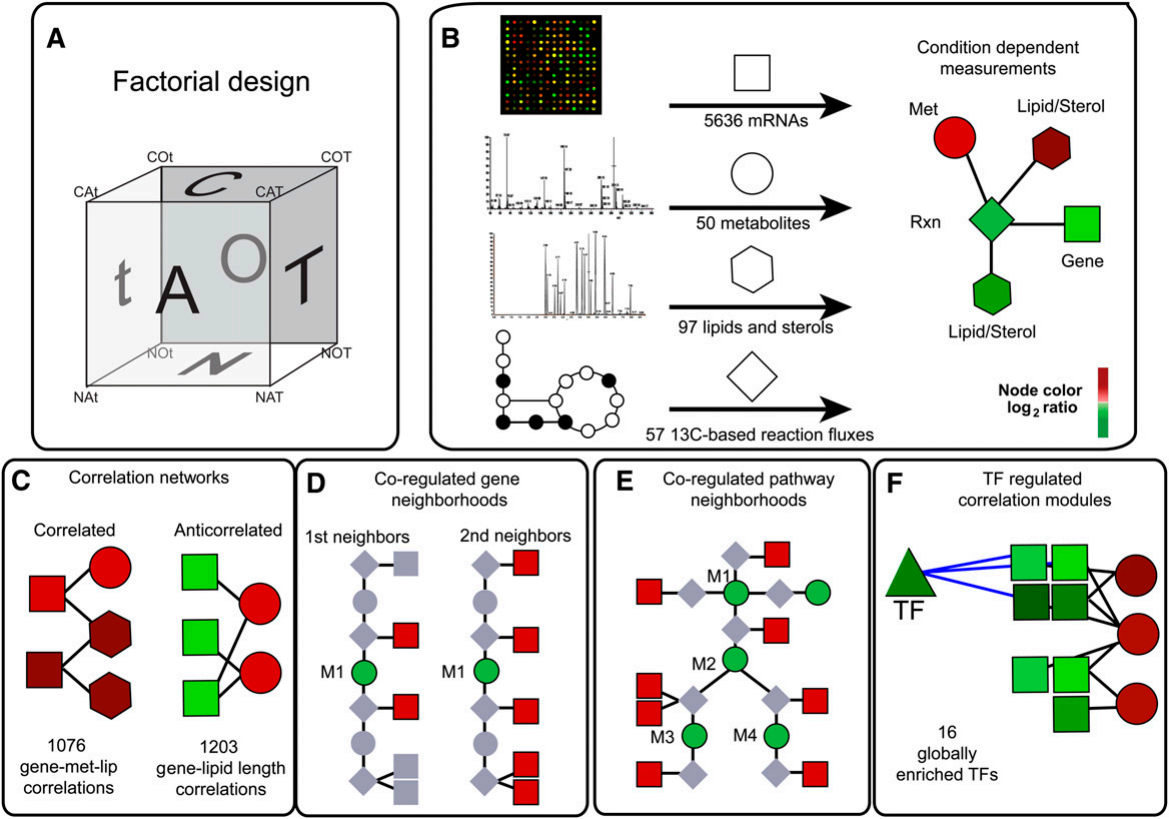
A systems approach to mapping condition-dependent lipid metabolism in the yeast *Saccharomyces cerevisiae*. (A) Cartoon representation of the 2 × 3 factorial design showing each experimental condition as a point on a cube (C-limited, C; N-limited, N; aerobic, O; anaerobic, A; 30°, T; and 15°, t). (B) For each condition (*e.g.*, COT), mRNAs, metabolites, lipids, and reaction fluxes were measured and mapped onto a metabolic model visualized in Cytoscape. (C–F) Integrative analyses used to query the measurement data. These included the identification of correlation networks (C), co-regulated gene neighborhoods (D), co-regulated pathway neighborhoods (E), and transcription factor (TF) correlated modules (F). (D and E) M1 represents a target metabolite in the reaction network. Node colors are meant to indicate a log_2_ color bar for measurement ratios, such as aerobic *vs.* anaerobic conditions.

## Materials and Methods

### Strain and chemostat cultivations

The reference laboratory strain *S. cerevisiae* CEN.PK113-7D (*MAT***a**) (van Dijken *et al.* 2000) was grown in well-controlled 2-liter jacketed chemostats (Braun Biotech) with a constant working volume of 1.0 liter. A factorial design was pursued with three factors comprising two levels each: carbon source [C-limited (C); N-limited (N)]; oxygen availability [aerobic (O); anaerobic (A)]; and temperature [30° (T); 15° (t)]. Cultivations were performed (in triplicates) at each of the eight conditions (COT, CAT, COt, CAt, NOT, NAT, NOt, NAt) for a total of 24 experiments. Fermentation conditions used a stirrer speed of 800 rpm, with pH of 5.0 (maintained by automatic addition of 2 N potassium hydroxide) and a dilution rate of 0.05 hr^−1^. Aerobic conditions were maintained by sparging the cultures with air (1.0 liter⋅min^−1^) and the concentration of dissolved oxygen was measured with Mettler Toledo polarographic electrode. Anaerobic conditions were maintained by sparging the medium reservoir and the fermentor with pure nitrogen gas (0.5 liter⋅min^−1^). Moreover, oxygen diffusion was minimized by using norprene tubing and butyl septa. The bioreactors were fitted with cooled condensers (2°–4°) and the off-gas was led to a gas analyzer (INNOVA and NGA 2000 Rosemount) to measure the content of CO_2_ and O_2_. Steady-state was reached when at least five but less than seven residence times had passed since starting the continuous cultivation and carbon dioxide evolution, dry weight measurements, and high-performance liquid chromatography (HPLC) measurements of extracellular metabolites were constant.

### Media

The medium composition was as previously described (Tai *et al.* 2005). For N-limited cultivations, residual glucose concentration in the chemostat was targeted to 17 ± 2 g⋅liter^−1^. This was to sustain glucose repression at the same level in all cultivations. To achieve the targeted glucose concentrations in the N-limited cultivations, the glucose feed concentration was 46 g⋅liter^−1^ for cells grown at 15° and 74 g⋅liter^−1^ for cells grown at 30°. The mineral medium composition for the N-limited cultivations was (amounts per liter) as follows: 1 g (NH_4_)_2_SO_4_; 3 g KH_2_PO_4_; 5.3 g K_2_SO_4_; 0.5 g MgSO_4_⋅7H_2_O; 1 ml Trace Metal Solution; 0.05 ml antifoaming agent; and 1 ml vitamin solution. The mineral medium composition for the C-limited cultivations was (amounts per liter) as follows: 5 g (NH_4_)_2_SO_4_; 3 g KH_2_PO_4_; 0.5 g MgSO_4_⋅7H_2_O; 1 ml Trace Metal Solution; 0.05 ml antifoaming agent; and 1 ml vitamin solution. The trace metal solution and vitamin solution are reported in Verduyn *et al.* (1992). The anaerobic cultivation medium was supplemented with 420 mg⋅L^−1^ Tween 80 and 10 mg⋅L^−1^ ergosterol (Pizarro *et al.* 2008). Antifoaming agent was used at a concentration of 0.15 ml/liter.

### Dry weight measurements

The concentration of biomass at steady-state was determined on a dry weight basis by filtering 5 ml of culture through a preweighed 0.45-µm nitrocellulose filter (Gelman Sciences, Ann Arbor, MI). The filter was washed with distilled water, dried in a microwave oven at 150 W for 15 min, and finally weighed to determine its increase in dry weight.

### RNA sampling and isolation

Samples for RNA isolation from aerobic cultivations were taken by rapidly sampling 20 ml of culture into a tube with 35–40 ml crushed ice to decrease the sample temperature to less than 2° in less than 10 sec. Cells were then centrifuged (4500 rpm at 0° for 3 min), instantly frozen in liquid nitrogen, and stored at −80° until further use. Sampling for RNA isolations from anaerobic cultivations was performed as described (Piper *et al.* 2002). Total RNA was extracted using FastRNA Pro RED kit (QBiogene, Inc, USA) according to manufacturer’s instructions after partially thawing the samples on ice. RNA sample integrity and quality was assessed before hybridization with an Agilent 2100 Bioanalyzer and RNA 6000 Nano LabChip kit.

### Probe preparation and hybridization to arrays

mRNA extraction, cDNA synthesis, cRNA synthesis and labeling, and hybridization to Affymetrix Yeast Genome 2.0 arrays were performed with a GeneChip one-cycle target labeling kit as described in the Affymetrix user’s manual (Affymetrix 2004). Washing and staining of arrays were performed using a GeneChip fluidics station 400 and scanning with an Affymetrix GeneChip Scanner 3000.

### Microarray data analysis

Raw microarray data (CEL files) were analyzed using R and BioConductor (http://www.bioconductor.org). Briefly, CEL files were normalized and the probe sets were summarized using the GeneChip RMA (GCRMA) package (Wu *et al.* 2003) and the “affinities” method. The CEL files and the normalized GCRMA table can be found in the ArrayExpress database (http://www.ebi.ac.uk/arrayexpress/) with accession E-MEXP-3704.

### Multi-way ANOVA

A linear model was applied to each gene to estimate the influence of each experimental factor (using C, O, and T here as abbreviations for C:N, O:A, and T:t respectively) and the potential interaction between pairs of factors (C:O, C:T, O:T) using an ANOVA approach (multi-way ANOVA). The model for mRNA expression each gene, *i*, was defined as:

$$y_{i}=\beta_{i0}+\sum_{j}^{F}\beta_{ij}x_{i}+\sum_{p\neq q}^{F}\beta_{ipq}x_{i}+\varepsilon_{i}$$

where parameters $\beta_{j}$ were fit for each experimental factor in set F = {C,O,T} and those for $\beta_{pq}$ were fit for interaction factors.

### Flux balance analysis

Estimation of *in silico* fluxes for all growth conditions was performed following a flux balance analysis (FBA) approach using the biopt software (Cvijovic *et al.* 2010). The specific medium and all measurements of highly secreted metabolites (*e.g.*, ethanol, glycerol, acetate) from our experiments were directly used as additional constraints in addition to the stoichiometry of the *iIN800* model (Nookaew *et al.* 2008), with the maximization of growth rate used as the objective function. The biomass equations were formulated based on specific lipid composition and carbon-limited or nitrogen-limited growth from each of the eight growth conditions.

### Biomass amino acid ^13^C enrichment analysis

^13^C-enriched biomass was achieved by chemostat experiments performed in in-house-built reactors with a working volume of 200 ml. Cultivations were performed at 30° or 15° with an agitation speed of 600 rpm, a dilution rate of 0.05 hr^−1^ (to match our other experiments), and pH of 5.0, and gas flow rate was kept at 1 vvm by air and N_2_ for aerobic growth and anaerobic growth, respectively. Steady-state samples were taken after five volume changes; 100% of the glucose used was labeled in position 1 (1-^13^C glucose was from Omicron Biochemicals Inc, USA). ^13^C-labeled biomass was harvested by centrifugation at 4000 rpm and 0° for 5 min. After centrifugation, the supernatant was poured off, and the cell pellet was frozen instantaneously in liquid nitrogen and stored at –80°. The samples were derivatized with both ECF derivatization and DMFDMA derivatization (Christensen *et al.* 2002). Derivatized samples were injected on a GC-MS as described previously to determine metabolite isotopes (Christensen *et al.* 2002). The flux distribution of any growth condition was calculated by the adopted model of Gombert *et al.* (2001) under the concept of summed fractional labeling (SFL) (Christensen *et al.* 2002).

### Endometabolome analysis

Cells were rapidly quenched according to de Koning and van Dam (1992) in 60% (v/v) buffered (12.5 mM Tricine, pH 7.4) cold methanol at –40 to –45°. After quenching, the cells were immediately centrifuged at 10,000 *g* for 4 min in a rotor precooled to −20° to separate the cells from the quenching solution. Chloroform:methanol:buffer (CMB) extraction was performed (Villas-Boas *et al.* 2005a; Villas-Boas *et al.* 2005b). After extraction, samples were freeze-dried at a low temperature (−56°) using a Christ-Alpha 1-4 freeze dryer (de Koning and van Dam 1992). Amino and nonamino organic acid levels were determined by HPLC on an Aminex HPX-87H column (Biorad) according to Fazio *et al.* (2008) and by GC-MS analysis according to Villas-Boas *et al.* (2005b), except that a Finnegan FOCUS gas chromatograph coupled to single quadrupole mass selective detector (EI; Thermo Electron Corporation, Waltham, MA) was used. Peak enumeration was conducted with AMDIS (NIST, Gaithersburg, MD) with default parameters, and identification of conserved metabolites was conducted with SpectConnect (Styczynski *et al.* 2007) using default parameters and a support threshold of 3. Samples were normalized by an internal standard chlorophenylalanine (30 μl of a 4 mM solution was added before extraction) and by the biomass weight per sample.

### Exometabolome analysis

Culture samples for determination of exometabolites were immediately filtered through a cellulose acetate filter (CAMEO 25GAS 0.22; Osmonics, Minnetonka, MN). GC-MS and HPLC analysis were performed as indicated. Samples were normalized by the biomass weight per sample.

### Lipid analysis

Samples for lipid isolation were taken by rapidly centrifuging at 4° and 10,000 rpm. The cell pellets were rinsed twice with DI water, instantly frozen in liquid nitrogen, and stored at −80° until lipid extraction. For quantification of lipid levels, internal standards [heptadecanoic acid, TG (17:0/17:0/17:0); phosphatidylcholine (PC) (17:0/17:0); PS (17:0/17:0); PI (17:0/17:0); phosphatidylethanolamine (PE) (17:0/17:0); ceramide (d17:1/18:1); and sitostanol] were added to each sample before extraction. The lipid extraction was performed using a modified Folch method (Folch *et al.* 1957) using a shaker at 4° with an agitation speed of 200 rpm overnight. The lipid extract was divided into three parts for further analysis of polar lipids, neutral lipids, and sphingolipid components. Using TLC, the polar lipid fraction was separated into PC, PS, PI, and PE using a solvent system consisting of chloroform:methanol:HAc:boric acid [40:20:30:10:1.8g (v/v/v/v/w)] and neutral lipid fraction was separated into sterols, free fatty acids (FFAs), TG, and steryl esters using a solvent mixture of hexane:diethylether:formic acid [80:20:1 (v/v/v)]. After development, lipid spots were visualized using 2,7-dichlorofluorescein and silica gel in the spots was carefully scraped off of TLC plates for analysis of fatty acid content and distribution of each lipid class. Specifically, glycerolipids were *trans*-methylated using a method combining alkaline methanolysis and BFl_3_-catalyzed *trans*-methylation described by Hamilton *et al.* (1992), which has been validated in-house and analyzed on a GC equipped with a flame-ionization detector (GC-FID), as described (Brix *et al.* 2010). Masses of each lipid class were calculated based on the peak area of the heptadecanoic acid released from the internal standards. The use of preparative TLC for separation of the different lipid classes before quantification of fatty acid content in each class using GC-FID analysis has been clearly established as a standard method, and when appropriate internal standards are used relative losses during methylation and extraction of the FAME from the silica are fully compensated by equal losses of the internal standards. Hence, quantification of each lipid class is highly reliable. Sterols were extracted from TLC plates following the validated method described previously (Zangenberg *et al.* 2004). The sterol extract was then derivatized with the TMS method as described previously (Nielsen and Madsen 2000) and injected into GC-MS equipped with 5% phenol column (Zebron column) and quantified in relation to the peak area for sitostanol. For analysis of the sphingolipid fraction, the lipid fraction was hydrolyzed using the method of Aveldano and Horrocks (1983). The long-chained base from the sphingolipids was extracted, derivatized with o-phtalaldehyde (OPA) reagent, and then analyzed by reverse-phase HPLC as described (Merrill *et al.* 1988). Sphingosine, phytosphingosine, C-20 sphingosine, and sphinganine were identified using authentic standards, and the content of sphingolipids was quantified based on the peak area of these compounds compared to the C17 long-chained base derived from the internal standard. Samples were normalized by the biomass weight per sample. All lipid data are presented as μmol/g dry cell weight (DCW), having been converted from mass units obtained from the GC-MS using the molecular weight for each species.

### Visual metabolic map

To gain an integrated perspective of environmental growth condition perturbations on gene, metabolite, and lipid expression, these data were visualized in Cytoscape (Cline *et al.* 2007) in a topological map based on the genome-scale metabolic model *iIN800*. Genes, metabolites, lipids, and *in silico* computed reaction fluxes were defined as nodes. These nodes were connected by one of three types of edges: gene (enzyme)-reaction; metabolite/lipid-reaction; and lipid distribution edges. Gene (enzyme)-reaction and metabolite/lipid-reaction edges were defined from the stoichiometry of the genome-scale model. Lipid distribution edges mapped acyl-chain composition for different species. For reasonable visualization, linear pathway reactions were merged, for example, the following set of reactions, PE + SAM → PMME + SAH, PMME + SAM → PDME + SAH, and PDME + SAM → PC + SAH was simplified to PE + SAM → PC + SAH for metabolites S-adenosyl-L-methionine (SAM), S-adenosyl-homocysteine (SAH), phosphatidylethanolamine (PE), phosphatidylmonomethylethanolamine (PMME), phosphatidyldimethylethanolamine (PDME), and phosphatidylcholine (PC). The simplified reaction PE + SAM → PC + SAH had two gene reaction associations: *CHO2* (encoding the enzyme catalyzing reaction PE + SAM → PMME + SAH ) and *OPI3* (encoding the enzymes catalyzing reactions PMME + SAM → PDME + SAH and PDME + SAM → PC + SAH). To help parse the complexity, we found it useful to visualize smaller networks, such as central metabolism, the TCA cycle and amino acid biosynthesis pathways, sterol biosynthesis, phospholipid biosynthesis, fatty acid biosynthesis, sphingolipid biosynthesis, and lipase degradation.

### Correlation analysis

The influence of gene expression on lipid and metabolite levels, or vice versa, was investigated using Pearson product-moment correlation coefficient, *r*. Correlation coefficients were calculated between transcript levels for all gene expression level–lipid and gene expression level–metabolite combinations across all 24 samples tested. The significance of each correlation coefficient was estimated (test for no correlation using *cor.test* method in R with standard “two-sided” alternative hypothesis) and the resulting *P* values were adjusted using the Bonferroni method to account for the number of gene expression level–lipid or gene expression level–metabolite combinations tested.

### Metabolic network gene neighborhood analysis

An extended yeast metabolic network based on *iIN800* that included acyl chain information and that contained 1754 reactions, 781 genes, and 897 lipids and metabolites was used to investigate regions of surprising correlation between lipids/metabolites and the mRNA of enzymes that may generate or utilize them (Nookaew *et al.* 2008). The modified reconstruction is provided in Supporting Information, Table S1. To achieve this, a neighborhood analysis for each lipid or metabolite was performed as follows. For each lipid or metabolite, the Pearson correlation coefficients to all neighboring enzyme transcripts (lipid–gene expression level or metabolite–gene expression level correlations) were compared to their correlation coefficients to all non-neighbor gene expression levels using a Mann-Whitney *U*-test (Wilcoxon rank-sum test). The nonparametric, two-sided hypothesis test provided a *P* value for each lipid and metabolite regarding how positively or negatively correlated it was to genes directly upstream or downstream in the metabolic network as compared to all other non-neighboring genes. In addition to this “first-neighbor” metabolic network analysis, a similar “second-neighbor” analysis was performed that defined neighbor correlations for all genes within two reaction steps of the lipid/metabolite in question.

### KEGG pathway analysis

Using the pathway definitions in KEGG to group sets of reactions and sets of lipids/metabolites, we performed a Mann-Whitney analysis similar to that applied to individual lipids/metabolites to assess the significance of correlation within a defined pathway represented by the mRNAs encoding the enzymes and the lipids/metabolites they produce. This test was performed to compare the sample of correlation values between lipids/metabolites and gene expression levels within a pathway to all other correlations between lipids/metabolites and gene expression levels not in the tested pathway.

## Results and Discussion

### Profiling of mRNAs, metabolites, and lipids in *S. cerevisiae*

To globally map networks that control lipid metabolism in yeast, we applied a systems approach integrating measurements across multiple cellular component layers (mRNAs, lipids, metabolites) and data from protein interaction databases with metabolic network topology (Figure 1). We pursued a full factorial design in which we varied different environmental conditions known to impact lipid metabolism (Figure 1A). Specifically, we varied the following three factors: carbon availability, known to impact fatty acid and phospholipid biosynthesis (Tehlivets *et al.* 2007; Carman and Han 2011); oxygen availability, known to impact sterol biosynthesis (Espenshade and Hughes 2007); and temperature, known to affect membrane composition (*e.g.*, fatty acid desaturation) and sphingolipid metabolism (Hunter and Rose 1972; Aguilar and De Mendoza 2006; Cowart and Obeid 2007). Each factor comprised two levels as follows: carbon source [carbon-limited (C); nitrogen-limited N)], oxygen availability [aerobic (O); anaerobic (A)]; and temperature [30° (T); 15° (t)]. Wild-type *S. cerevisiae* CEN.PK113-7D was grown in chemostat cultivations at a fixed dilution rate *D* = 0.050 hr^−1^ to avoid confounding affects associated with the dynamic nature of batch cultures or attributable to different specific growth rates under these conditions (Regenberg *et al.* 2006).

From each chemostat cultivation, we measured mRNA, metabolite, and lipid levels (Figure 1B). We were able to detect and quantify a total of 5636 mRNAs, 50 metabolites, and 97 lipid species. The large number of measured lipids arises from having data regarding acyl-chain composition for different lipid species (*i.e.*, the fatty acid composition of phospholipid species; C12:0, C16:1, and others). Detailed physiological data, carbon balances, and complete datasets are provided in Table S2 and Table S3.

Despite similar specific growth rates, marked changes were observed at the molecular level. For example, our lipid measurements confirmed that lipid class levels vary based on the three types of environmental conditions evaluated (Figure S1, Figure S2, Figure S3, Figure S4, Figure S5, Figure S6, Figure S7, Figure S8, and Figure S9). We used a multi-factor ANOVA to identify significant changes at the mRNA, metabolite, and lipid levels (Table S4). In this way, the main experimental factors tested (*i.e.*, C:N, O:A, T:t) and, in addition, all the possible interaction factors were tested (*i.e.*, CN:OA, CN:Tt, OA:Tt, and CN:OA:Tt) to identify mRNAs, metabolites, and lipids significantly influenced by combinations of growth conditions, highlighting the power of the full factorial design. At a threshold of *P* ≤ 0.001 after Bonferroni correction (Bonferroni 1936), we observed 1855 differentially expressed genes by one or more of the tested design factors (including interaction factors), thus showing that one-third of the 5636 measured genes were significantly affected by one or more of the investigated factors. Interestingly, temperature and carbon availability induced a comparably large number of gene expression changes (951 and 910, respectively, at *P* ≤ 0.001). By comparison, aerobicity appeared to influence expression of approximately half as many genes (516 at *P* ≤ 0.001). By contrast, the lipidome data were most influenced by the presence or absence of oxygen. A majority of the 97 lipids measured were significantly changing due to at least one of the tested factors (78 at P \x{2264} 0.001). Aerobicity accounted for changes in two-thirds (65) of the lipids tested. C:N and T:t accounted for 47 and 30 lipid changes, respectively. Two-thirds (33) of the 50 metabolites measured were changing because of at least one factor. C:N and O:A each affected approximately half (24 and 21, respectively), whereas T:t only influenced approximately one-third (17) of the 50 metabolites measured. The overlaps among significant genes, lipids, and metabolites are highlighted in the Supporting Information (Figure S10 and Table S4).

**Figure 2:**
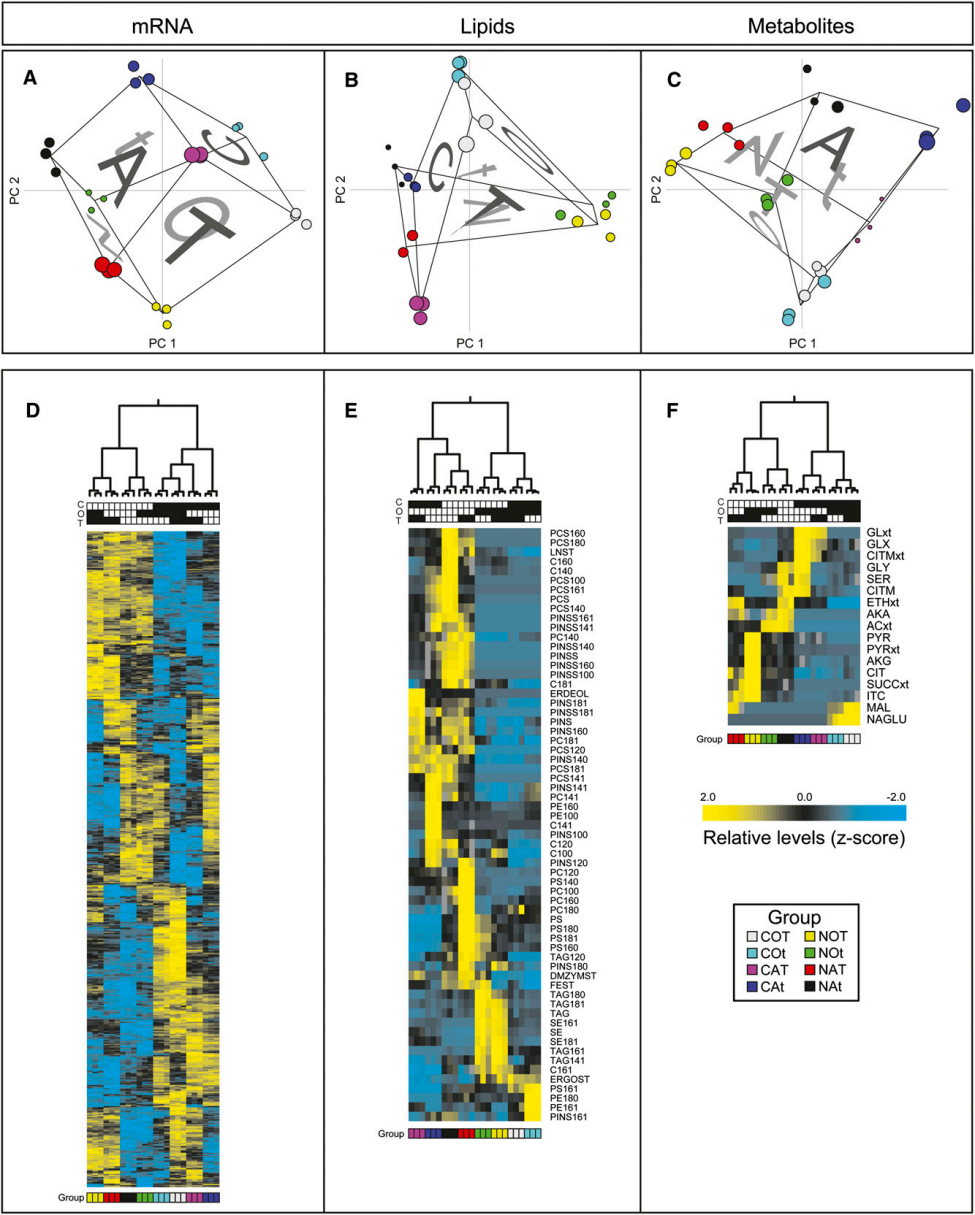
Molecular signatures of mRNAs, lipids, and metabolites for each experimental condition: C-limited, C; N-limited, N; aerobic, O; anaerobic, A; 30°, T; and 15°, t (COT, gray; COt, cyan; CAT, purple; CAt, blue; NOT, yellow; NOt, green; NAT, red; NAt, black). (A–C) Principal components analysis (PCA) with projections for PC 1, PC 2, and PC 3 for all mRNA, lipid, and metabolite levels, respectively. The size of the point represents location in the PC 3 axis, which is perpendicular to the page. (D–F) Hierarchical clustering and resulting heat maps for mRNAs, lipids, and metabolites found to be significant by ANOVA. Measurement *z*-scores for all data types were mapped as defined by the color bar (note that mRNA *z*-scores were based on GCRMA values which are log_2_). For gene, lipid, and metabolite name abbreviations, see Table S6.

Principle components analysis (PCA) was used to provide a nonbiased means of assessing the influence of growth factors (C:N, O:A, and T:t) on mRNA, metabolite, and lipid levels (Figure 2). Because this analysis considered all measured quantities of each type, it provided a more general overview than the multi-factor ANOVA analysis. Strikingly, when the first three components of our transcriptome data were plotted in three-dimensions, the six faces of a cube representing our three-factor experimental design were observed (Figure 1A and Figure 2A). Together, the first three components accounted for >75% of the variation in the mRNA expression data (Table S5). The cube of factor combinations was tilted and rotated in the first three PCA dimensions, indicating that each of these dimensions was influenced by all three factors but to differing extents. The first principle component (37% of mRNA variance) was primarily attributable to C:N factor; the second dimension (24% of mRNA variance) was an approximately equal balance of all experimental factors, whereas the third dimension (15% of mRNA variance) was primarily attributable to aerobicity (O:A). This indicates that mRNA levels were most influenced by C-limitation and N-limitation, which makes sense when we consider that few cellular process are independent of carbon and nitrogen utilization. The PCA analysis of the metabolite data attributed >80% variance to the first two PCA dimensions (63% to PC 1), with C:N as the primary source of variability. Metabolite PC 1 showed similarity to mRNA PC 2 and, vice versa, PC 2 showed similarity to mRNA PC 1. By contrast, lipidome data displayed the greatest variance along the aerobic–anaerobic split (Figure 2, A–C). Because lipid levels are controlled by a subset of metabolic processes and enzymes, they do not necessarily vary on aggregate as metabolites and mRNAs do. Lipids were separated in the PC 1 between aerobic and N-limited *vs.* the rest and in PC 2 by aerobic and C-limited *vs.* the rest. Because the first two PCA dimensions accounted for ∼80% of the variance in the lipid data, we can conclude that aerobicity had the largest influence on the lipid levels, whereas C:N was secondary. A hierarchical clustering of these datasets showed the same trends arising from the environmental conditions (Figure 2, D–F).

### Conditional dependence of molecular players based on environmental perturbation

**Figure 3:**
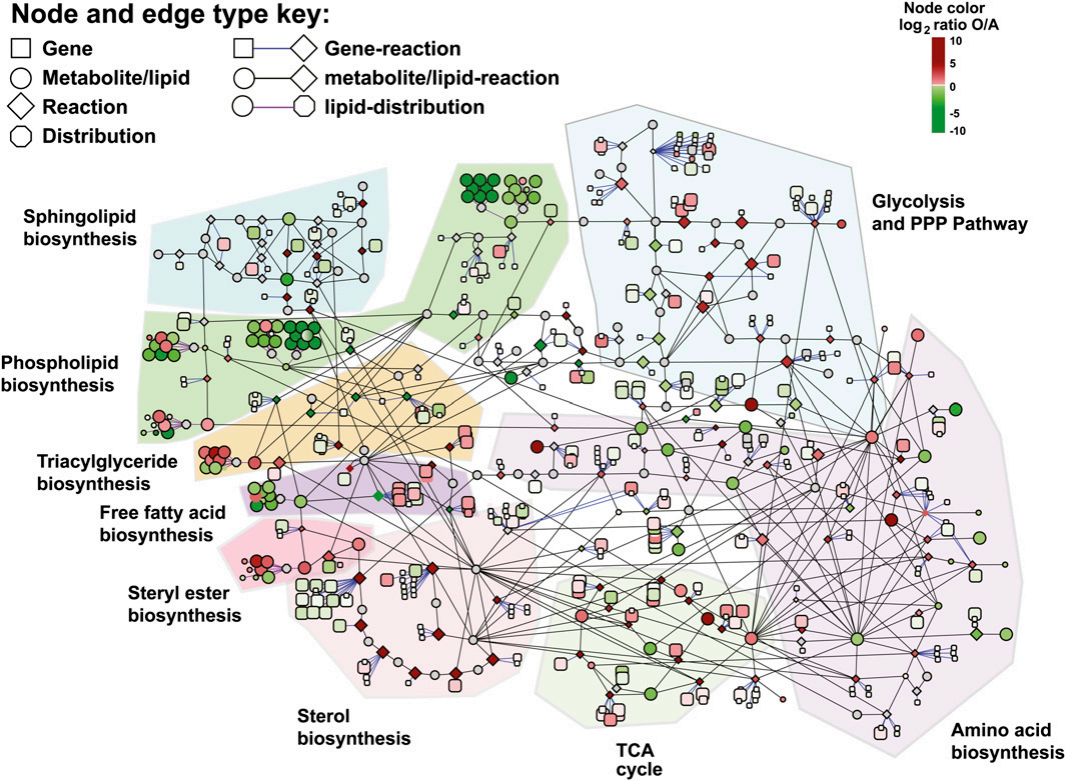
Metabolic map for interrogating the connectivity of mRNAs, metabolites, lipids, and reaction fluxes based on the genome-scale model *iIN800*. This network contains gene (enzyme)-reaction, metabolite/lipid-reaction, and lipid distribution edges (see node type and edge key). Measurement ratios can be visualized with log_2_-fold changes (see node color key; here, shown for aerobic *vs.* anaerobic conditions) and gray indicates the lack of a measurement for that node. Individual pathway networks (*e.g.*, phospholipid biosynthesis, sterol biosynthesis, and amino acid biosynthesis) are highlighted. Findings for individual pathways are discussed in the text.

To determine the conditional dependence of mRNA, metabolite, and lipid data, we first constructed a visual map based on the genome-scale metabolic model, *iIN800* (Nookaew *et al.* 2008). Because we lacked measurements for many of the metabolites in *iIN800*, we centered our biosynthetic network on central metabolism, the tricarboxylic acid (TCA) cycle, amino acid biosynthesis, sterol biosynthesis, steryl ester biosynthesis, triacylglyceride (TAG), biosynthesis, FFA biosynthesis, phospholipid biosynthesis, and sphingolipid biosynthesis. In total, our visual network map consisted of 368 gene, 174 metabolite/lipid, and 131 reaction nodes connected by 849 gene (enzyme)-reaction and metabolite/lipid-reaction edges (Table S6). Nine distribution nodes were also included for mapping information on lipid acyl-chain composition. A distribution node is connected to all of the acyl-chain species involving that particular lipid or fatty acid type. For the reaction nodes, we performed flux balance analysis to estimate values of reaction fluxes *in silico* under the constraints of maximized biomass production, a steady-state metabolic network, and fixed protein composition (Table S7) (Nookaew *et al.* 2008). Our network model linking all measurement types was visualized in Cytoscape (Cline *et al.* 2007) as shown in Figure 3 [with color mapping for measurement log-fold-changes, such as aerobic (O) *vs.* anaerobic (A) conditions]. Although the interplay between different metabolic pathways could be missed in our visualization approach, we have previously shown that such a model provides a useful framework for exploring the connectivity of mRNAs, metabolites, lipids, and fluxes (Moxley *et al.* 2009). Additionally, subsequent integrative analyses we performed for this work were not solely based on this visualization and contain the entire metabolic network (see *Data integration through hypothesis testing across multiple cellular levels* section).

Visualization in Cytoscape enabled the targeted and rapid identification of changes across multiple levels of the cellular hierarchy (Figure S11 and Figure S12). These networks provided a direct route for querying cellular changes one at a time and validated the high quality of our data (Figure S13). For example, we observed the downregulation of acetyl-CoA carboxylase (*ACC1*), which catalyzes the carboxylation of acetyl-CoA to form malonyl-CoA as the first committed step of *de novo* fatty acid biosynthesis when comparing growth at 30° to 15° (Figure S13E). This was positively correlated to a reduction in levels of pyruvate, alpha-ketoglutarate, malate, and citrate (Figure S13G). Citrate is known to allosterically activate Acc1p (Martin and Vagelos 1962).

**Figure 4:**
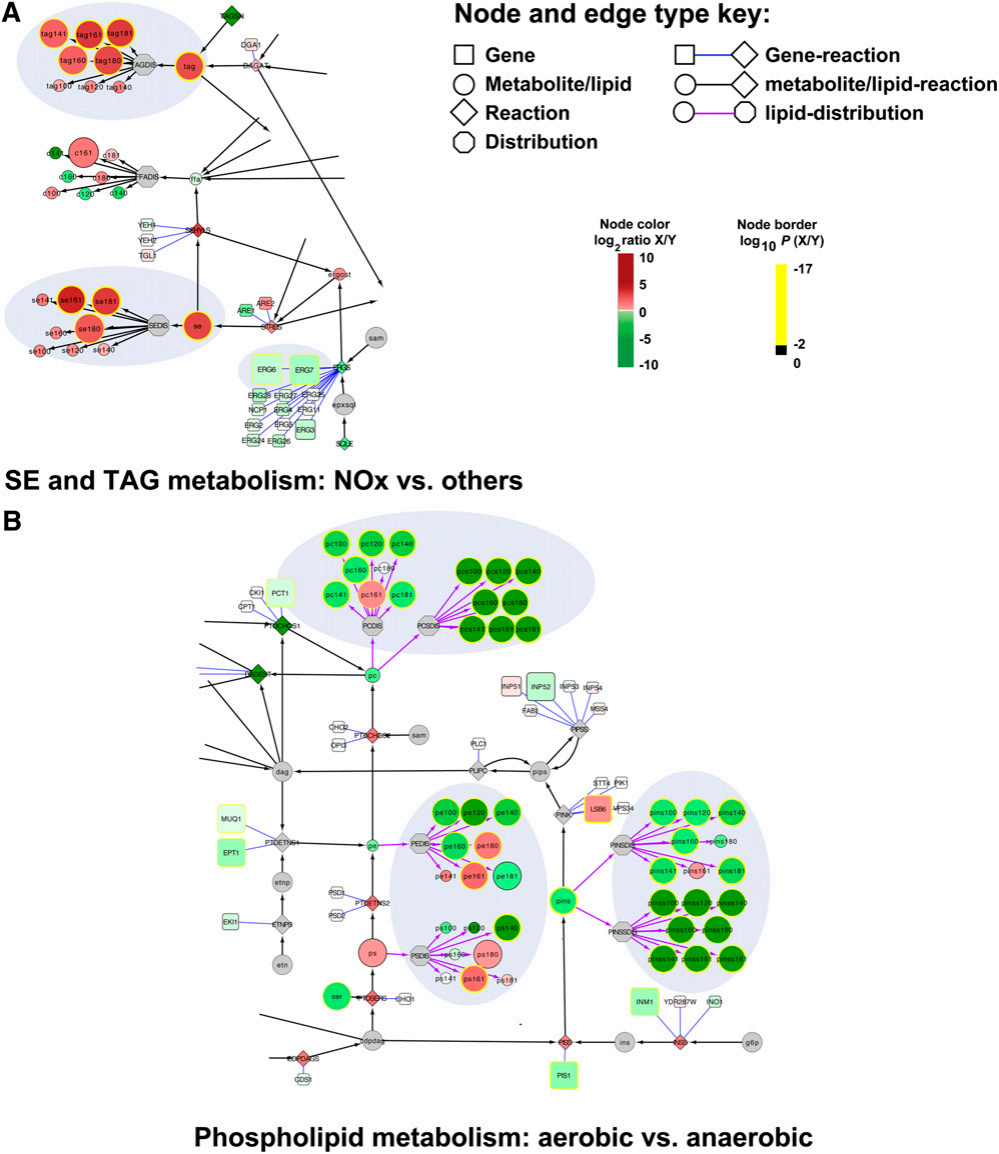
Condition-dependent response of lipid metabolism. (A) The impact of nitrogen-limited aerobic conditions (NOx = NOT and NOt) *vs.* all others on the storage depots triacylglycerol (TAG) and sterylesters (SE). TAG and SE significantly accumulate under NOx conditions and are inversely correlated to *ERG7* and *ERG6*. (B) The impact of aerobicity on phospholipid metabolism. We observed significant increases in phosphotidylethanolamine (PE) and phosphotidylcholine (PC) and in phosphatidylinositol (PINS) species under anaerobic growth. Uniquely, the presence of di-substituted medium acyl-chain fatty acids, which we denote with an “S” (*e.g.*, PCS or PINS), under anaerobic growth suggested redox constraints. Measurement ratios were visualized with a log_2_ color bar (see node color key). The relative node size, thickness of each node border, and color of each node border represents the log_10_(*P* value). See node key. Gray indicates the lack of a measurement for that node.

Some of the most dramatic changes were observed for the storage depots of the cell, TAG and steryl esters (SE) (Figure 4A and Figure S2). Under nitrogen-limited (*i.e.*, carbon excess) and aerobic conditions, levels of TAG increased from 0.7 ± 0.2 μmol/g DCW to 3.8 ± 0.2 μmol/g DCW, and levels of SE increased from 0.8 ± 0.6 μmol/g DCW to 4.9 ± 0.4 μmol/g DCW. When compared to all other conditions (N-limited aerobic *vs.* others), only 26 genes, 11 lipids, and 3 metabolites were significantly changed (*P* ≤ 0.01 following Bonferroni correction) (Table S8). Within this small subset, it is striking that none of the biosynthetic genes encoding enzymes that directly produce diacylglycerol (DAG), TAG, ergosterol, or SEs is significantly upregulated. Rather, we observed a confluence of changes that contribute to the observed phenotype, including high levels of pyruvate and alpha-ketoglutarate (key signaling molecules indicating high carbon levels), more reducing power (flux through the pentose phosphate pathway increases), downregulation of regulatory proteins (*e.g.*, *IZH4*, *DAN3*, *HES1*) (Carman and Han 2007), and a lower flux toward phosphatidic acid (the precursor to *de novo* phospholipids synthesis) (Carman and Han 2011). Consistent with the apparent shift from production of phospholipids to the production of storage compounds, the ratio of nonpolar lipids (sterols, TAG, SE, and FFAs) to phospholipids was higher under nitrogen-limited aerobic conditions (Figure S14). We also observed an inverse regulatory relationship between the expression of *ERG6* and *ERG7* and TAG and SE levels (Figure 4A). Erg6p and Erg7p are involved in sterol biosynthesis and are found almost exclusively in lipid particles (Schulz and Prinz 2007) interacting with Are1p and Are2p, respectively. Because Are1p and Are2p catalyze SE biosynthesis, our data are consistent with the hypothesis that Erg6p and Erg7p may play a regulatory role in SE synthesis (Czabany *et al.* 2007; Teske *et al.* 2008).

Our visual metabolic model also highlighted changes in phospholipid metabolism. Phospholipid levels varied significantly with temperature and oxygen availability. Globally, this trend is a result of increased phosphotidylinositol (PINS) levels, as well as increased PC and PE levels at lower temperatures under anaerobic conditions (Figure S3, Figure S4, Figure S5, and Figure S7). Uniquely, the distribution nodes of our model enabled mapping of quantitative acyl-chain composition information (Figure 4B). After a shift from aerobic to anaerobic growth, we observed the production of a population of phospholipids with di-substituted medium acyl-chain (C10:0, C12:0) fatty acids (Figure S3 and Figure S4). To our knowledge, this fatty acid species has not previously been reported as a major component inside the cell. The presence of di-substituted medium acyl-chain fatty acids, which we denote with an “S” (*e.g.*, PCS), under anaerobic growth suggested redox constraints. Because the fatty acid synthase uses two reduced NADPH for each 2C-atom added to a growing fatty acid, we hypothesized that a decrease in reducing power would hamper fatty acid elongation and cause a shift toward production of medium-chain fatty acids. The flux through the pentose phosphate pathway (PPP) is relatively low under anaerobic conditions, causing cells to be redox-constrained. Previously, for example, it was shown that NADPH is balanced under fermentative growth conditions, whereas excess NADPH is produced under aerobic conditions (Gombert *et al.* 2001). In addition, our *in silico* flux balance analysis (Table S7) revealed a 10-fold average decrease in the PPP flux when comparing anaerobic to aerobic conditions. To experimentally validate this hypothesis, we performed follow-up ^13^C-flux analysis experiments. To experimentally determine fluxes, we cultivated cells on 1-^13^C-glucose and experimentally measured isotopic labeling patterns with GC-MS. We observed that measured flux through the PPP is reduced 3.5-fold in anaerobic conditions (*P* ≤ 0.01) (Figure S15), which is consistent with our *in silico* fluxes and the previous work of Gombert *et al.* (2001).

To reveal how the transcriptional network was reprogrammed across different environmental conditions, we scored the significance of overlap between condition-dependent genes, *i.e.*, genes found significant for each tested experimental factor by multi-factor ANOVA, and known transcription factor (TF) target sets (hypergeometric test at *P* ≤ 0.01) (Harbison *et al.* 2004; Beyer *et al.* 2006; Fazio *et al.* 2008). In total, this analysis revealed 13 TFs having significantly enriched target sets for four of the six factors (C, O, T, C:O) (Figure S16). For example, 14 of 17 known Upc2p regulatory targets (Hodges *et al.* 1998) were found to have significantly lower expression in aerobic conditions relative to anaerobic conditions. Although these TFs appear to be responsible for regulating some of the differentially expressed genes, the relative number of differentially expressed genes targeted was less than 10% of the affected genes in all cases.

### Data integration through hypothesis testing across multiple cellular levels

**Figure 5:**
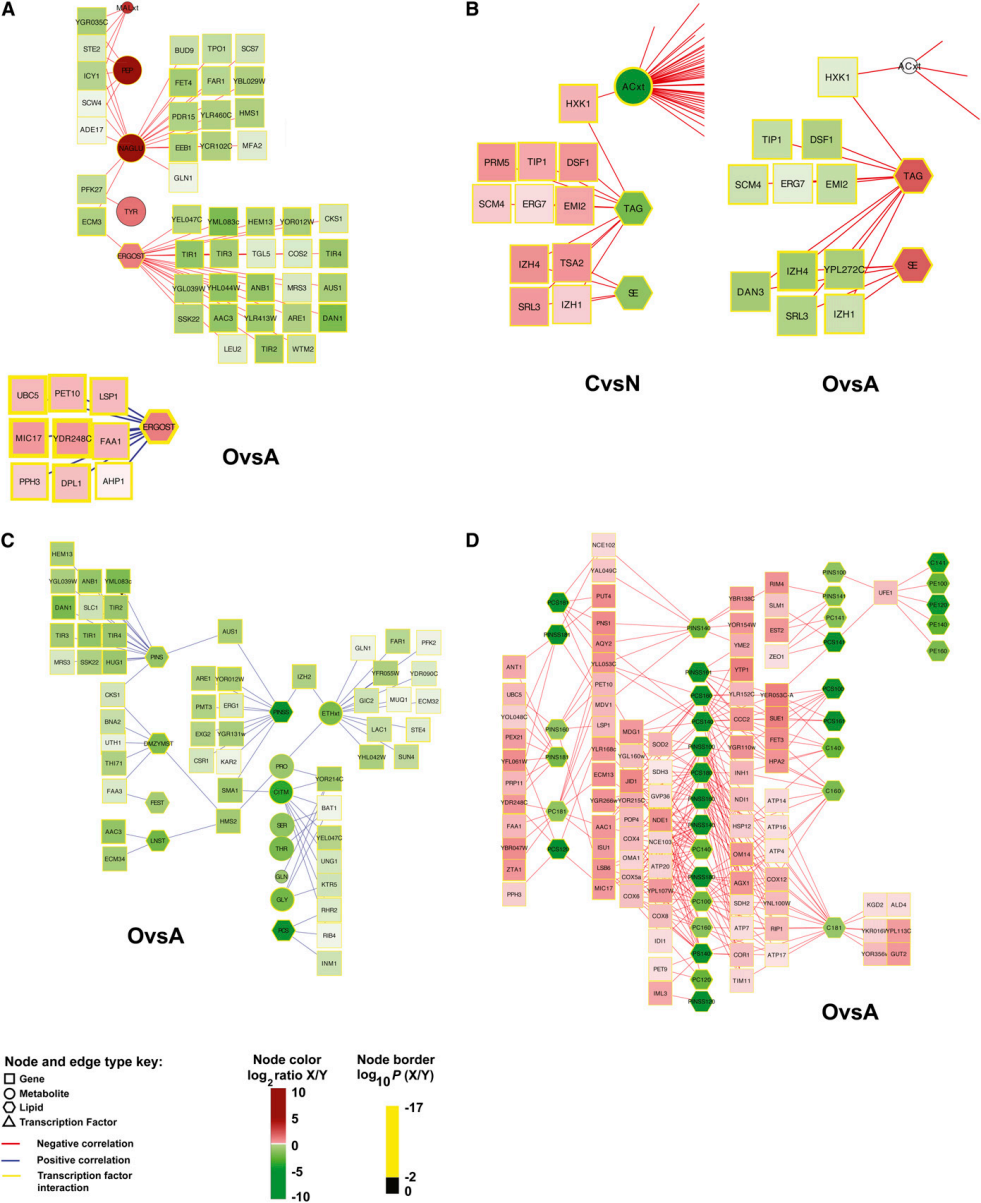
Correlation analysis enabled data integration across multiple levels of the cellular hierarchy. Highly correlated and anti-correlated relationships between gene expression levels–metabolites, gene expression levels–lipids, and gene expression levels–lipids as characterized by acyl-chain length are shown. (A) Correlations with ergosterol (ERGOST) under aerobic (“O”) and anaerobic (“A”) conditions. (B) Correlations with SE and TAG under C-limited (“C”) *vs.* N-limited (“N”) conditions and aerobic *vs.* anaerobic conditions reveal condition-independent conditions (*e.g.*, *IZH1*, *ERG7*, and *HXK1*). (C) Positive correlations with phosphatidylinositol (PINS) under aerobic *vs.* anaerobic conditions. (D) Correlations between gene expression levels and lipids as characterized by length under aerobic *vs.* anaerobic conditions. Measurement ratios were visualized with a log_2_ color bar, and the color of each node border represents the log_10_(*P* value). See node and edge color key. Gray indicates the lack of a measurement for that node. Complete networks are found in Figure S17, Figure S18, Figure S19, Figure S20, Figure S21, and Figure S22.

Beyond interrogating data types one component at a time, we sought to address the pluralism of causes and effects in biological networks by developing integration strategies for observing regulatory signatures. This is one of the grand challenges of 21^st^ century systems biology (Palsson and Zengler 2010; Bebek *et al.* 2011). To identify the most highly correlated and anti-correlated relationships between gene expression level and metabolites, gene expression level and lipids, and gene expression level and lipids as characterized by acyl-chain (Figure 1C), we tested whether the Pearson correlation coefficient, as estimated over all triplicates in the eight conditions (24 measurements), was significantly not zero (*P* ≤ 0.001 after Bonferroni correction). Although experimentally determined or computed fluxes were not directly integrated into this analysis, fluxes are indirectly considered because the lipid levels in the biomass were directly correlated to the fluxes when we used chemostat cultures and lipid levels, which are given per unit biomass. Our analysis revealed 831 significant correlations among gene expression levels and metabolites, 245 significant correlations among gene expression levels and lipids, and 1203 significant interactions among gene expression levels and lipid species characterized by length. Relationships were then separated into positive and negative correlations and filtered based on ANOVA significance main effects (*e.g.*, C-limited *vs.* N-limited) for visualization in Cytoscape. Gene expression level-metabolite and gene expression level–lipid interactions were combined into a single network, leaving gene expression level–lipid by acyl-chain composition correlations separate. The organization of the network contained mainly single-connection, chain, and bi-fan motifs (Figure 5, Figure S17, Figure S18, Figure S19, Figure S20, Figure S21, and Figure S22). There were 2279 significant correlations.

Because of our full factorial design, these data offer a rich resource for exploring correlations across mRNAs, lipids, and metabolites influenced by broad perturbations that were not possible before (Figure 5). Although significance of correlation was assessed across all eight conditions, in some examples, the correlation was evident from changes attributable to a single factor. For example, when comparing aerobic to anaerobic conditions, ergosterol levels are correlated to nine mRNA levels and anti-correlated to 23 mRNA levels (Figure 5A). Identification of gene expression levels that have been previously implicated in controlling sterol levels validated our approach. Erg1p, for example, encodes for squalene synthase, an enzyme in the ergosterol biosynthesis pathway that requires oxygen (Lees *et al.* 1999; Espenshade and Hughes 2007). Aus1p is a sterol transporter required for growth under anaerobic conditions (Lees *et al.* 1999; Wilcox *et al.* 2002; Espenshade and Hughes 2007). As expected, Aus1p is anti-correlated to ergosterol levels when comparing aerobic and anaerobic conditions. In other cases, we observed more than one factor strongly influencing the correlation. For example, nine gene expression level–lipid correlations for TAG and SE were observed in both O:A and C:N conditions (Figure 5B), suggesting strong regulatory control. Genes linked to TAG and SE included *HXK1*, *IZH4*, *IZH1*, *SRL3*, *ERG7*, *TIP1*, *DSF1*, *SCM4*, and *EMI2*. As discussed, Erg7p is known to interact with Are1p, which contributes the major sterol esterification activity under anaerobic conditions (Czabany *et al.* 2007). The family of Izh proteins is involved in zinc ion homeostasis, which regulates phospholipid synthesis (Carman and Han 2007).

Correlation between mRNA and metabolite levels may depend on more than one factor, and potentially in differing ways. For example, phosphoenolpyruvate (PEP) levels were observed to increase when *ILV2* or *ARO3* levels increased in N-limiting conditions, but PEP was negatively correlated to these genes in C-limiting conditions (Bradley *et al.* 2009). To check for such condition-dependent and opposing correlations, we performed analyses to test for gene expression level–metabolite or gene expression level–lipid pairs that were, for example, significantly positively correlated with C-limited trials and significantly negatively correlated with N-limited trials, or vice versa. This was achieved by splitting the data in half by each of the three factors, testing both halves of each split separately and comparing the results. No gene expression level–metabolite or gene expression level–lipid pairs were observed to have significant and opposite correlations in the data set halves, although some pairs with agreeing correlations were observed.

Our correlation analysis enabled exploration of two key features that would not have otherwise been observed by mapping data onto our visual metabolic model alone (Figure 3 and Figure 4). First, metabolites and lipids were linked in ways not found in traditional metabolic maps. In other words, metabolites and lipids not sharing the same metabolic enzymes were correlated. For example, extracellular ethanol levels were linked to phosphatidylinositol through by *IZH2* and *AUS1* (Figure 5C) and phospholipid species with di-substituted medium acyl-chain fatty acids were connected to genes known to impact redox metabolism (Figure 5D), an observation consistent with our ^13^C-flux analysis. Second, the significance of metabolic genes that are not present in the metabolic model (Figure 3 and Figure 4) could be identified. Although biosynthetic metabolic genes in the correlation networks were slightly enriched relative to their percentage in yeast (19% *vs.* 13%), only 5 of 2279 correlations were between first neighbors and direct substrates or products (Table S9). At a quantitative systems level, this suggests that biosynthetic enzymes that either use metabolites and/or lipids as reactants or catalyze their formation were not directly connected. In other words, transcriptional regulation is not tightly controlled at the one-step level.

### Integrative methods for correlation of omics data

Because lipids/metabolites and gene expression levels were not co-regulated at the one-step level, we next sought to refine the correlation analysis using the following three parallel and independent approaches: co-regulated gene neighborhoods (Figure 1D); co-regulated pathway neighborhoods (Figure 1E); and TF-regulated modules (Figure 1F). Identification of such neighborhoods and modules would lead to a better understanding of the underlying organization of the cellular response. For co-regulated gene neighborhoods, we created a topology bi-partite map, whereby metabolites were connected to genes that encode for proteins that catalyze reactions that include a metabolite as a reactant or product. The metabolic network consisted of 945 metabolites and 715 protein-coding genes in which a median of two genes were connected to each metabolite and a median of four metabolites were connected to each gene. We then determined if gene neighbors for each metabolite/lipid had a bias to be significantly correlated (*r* > 0) or anti-correlated (*r* < 0).

We initially looked at first gene neighbors within the metabolic network (Figure 1D). Using this approach, a significant bias would suggest that metabolite production/consumption is driven by differential expression of upstream or downstream enzymes in the metabolic network. First gene neighbors did not show any bias relative to that expected of a random sample (Mann-Whitney *U*-test). However, 22 metabolites and lipid species had a significant bias when considering both first and second neighbors (*P* ≤ 0.01, Benjamini Hochberg *P* value adjustment) (Table 1), with ergosterol being the most highly correlated. Our observations provide new systems-level insight, suggesting that sterols, and perhaps phosphatidylinositol, are more regulated across the range of different growth conditions tested here than amino acids at the transcriptional level (Table S10). Given that amino acids are chiefly directed into proteins, this suggests that the cell has developed more complex methods to regulate proteins post-transcriptionally than sterols and the entry point into phospholipid biosynthesis. Furthermore, biosynthesis of amino acids involves far more branched pathways than the biosynthesis of lipids; to ensure a balanced supply of the 20 different amino acids required for protein biosynthesis, cells have evolved complex regulation of flux at the enzyme level. This finding is consistent with our recent work (Moxley *et al.* 2009) demonstrating that amino acid biosynthesis is primarily metabolically controlled.

**Table 1:** Metabolites and lipids whose levels are most significantly driven by expression of neighboring genes in a bi-partite genome-scale metabolic map

MET/LIP	p.BH	n.MetMap.linked	n.PCC.sig	n.linkedANDsig
ERGOST	7.97E-08	102	76	9
PEP	4.56E-06	293	80	7
LNST	9.69E-06	105	98	7
SUCC	4.68E-05	176	5	1
MAL	4.68E-05	244	11	2
EPST	5.91E-05	69	4	0
DMZYMST	7.83E-05	70	55	2
PINS181	0.000236	203	29	2
NADPH	0.000761	480	0	0
NADP	0.000926	503	0	0
PINS160	0.00227	203	38	0
PINSS181	0.00247	172	33	1
ALA	0.00247	336	11	4
PINS	0.00304	203	56	2
SE161	0.00304	54	20	1
PYRxt	0.00332	67	32	1
TAG100	0.00401	60	58	2
PROxt	0.00401	165	18	1
TAG140	0.00401	60	91	3
LYS	0.00405	336	5	1
ZYMST	0.00521	74	0	0
PYR	0.00729	369	22	3

MET/LIP: metabolites/lipids; p.BH, *P* ≤ 0.01, Benjamini Hochberg *P* value adjustment; n.MetMap.linked, number of genes linked to the specified metabolite in the genome-scale metabolic map; n.PCC.sig, number of genes significantly correlated to the specified metabolite (*P* ≤ 0.01 Bonferroni *P* value adjustment); n.linkedANDsig, number of metabolites both linked in the genome-scale metabolic map and significantly correlated (*i.e.*, the overlap between the previous two columns); ERGOST, ergosterol; PEP, phosphoenolpyruvate; LNST, lanosterol; SUCC, succinate; MAL, malate; EPST, episterol; DMZYMST, 4,4-dimethylzymosterol; PINS181, 18:1 in phosphatidylinositol; NADPH, nicotinamide adenine dinucleotide phosphate (reduced form); NADP, nicotinamide adenine dinucleotide phosphate; PINS160, 16:0 in phosphatidylinositol; PINSS181, 18:1 (Δ^9^) in phosphatidylinositol; ALA, alanine; PINS, phosphatidylinositol; SE161, steryl ester 16:1; PYRxt, pyruvate (extracellular); TAG100, 10:0 in triacylglycerol; PROxt, proline (extracellular); TAG140, 14:0 in triacylglycerol; LYS, lysine; ZYMST, zymosterol; and PYR, pyruvate.

Another way to refine the correlation analysis is to look for transcriptional regulation across genes in pathways (Figure 1E), rather than first-order or second-order gene neighbors (Figure 1D). We therefore tested to see if gene neighbors for sets of metabolites in a KEGG pathway, regardless of reaction direction (Kanehisa *et al.* 2012), have a bias to be significantly correlated or anti-correlated. This approach, therefore, is testing which pathways contain metabolites that tend to correlate with all transcripts in the pathway, as a group, rather than our gene neighbor analysis, which reveals specific metabolites that tend to correlate with their neighboring genes. Although KEGG pathways could be isolated from global metabolic networks that were captured in our gene neighborhood analysis because of choices of database curators, the results were consistent with our co-regulated gene neighborhood analysis (which is unbiased). For example, we observed that the sterol biosynthesis KEGG pathway was significantly co-regulated (*P* ≤ 0.01, Benjamini Hochberg *P* value adjustment) (Table S11), validating the utility of using a systems perspective to integrate omics information.

**Figure 6:**
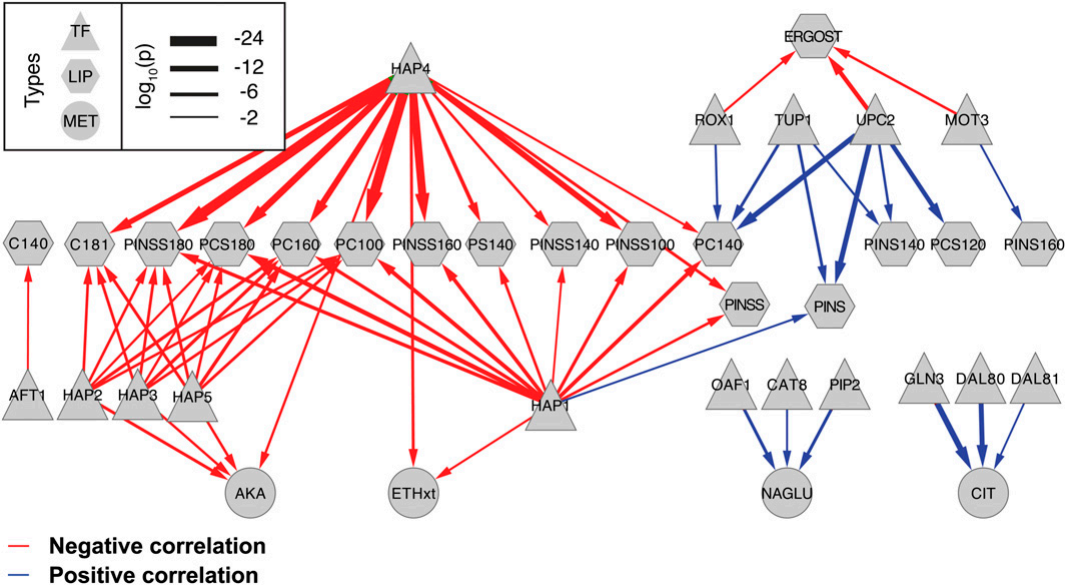
Transcription factor associations with genes correlated to lipid or metabolite levels. The Fisher exact test was used to identify significant overlaps between transcription factor target sets and genes significantly correlated (blue arrows) or anti-correlated (red arrows) to the implicated lipid or metabolite. The relative thickness of each arrow represents the −log_10_(*P* value) from the Fisher exact test.

In an effort to identify factors controlling co-regulated correlation networks, smaller sets of significantly changing genes identified as significantly correlated to specific metabolites or lipids were analyzed for enrichment of TF targets (Figure 1F). This analysis identified 16 TFs significantly associated with four metabolites and 15 different lipid species through the gene sets levels to which they were significantly correlated. Positively and negatively correlated gene expression level–lipid and gene expression level–metabolite sets were analyzed separately. Figure 6 shows the significant (hypergeometric *P* ≤ 0.01) TF gene expression level–lipid and TF gene expression level–metabolite enrichments capture a global regulatory model of lipid metabolism. The Hap family of TFs and, particularly, Hap4p and Hap1p were found associated with genes negatively correlated to a number of lipid levels. Interestingly, Upc2p and Rox1p targets were enriched for genes positively correlated to PINS, PINSS, PC, and PCS, and were negatively correlated to ergosterol. These regulators are known players in sterol regulation (Espenshade and Hughes 2007; Nielsen 2009).

**Figure 7:**
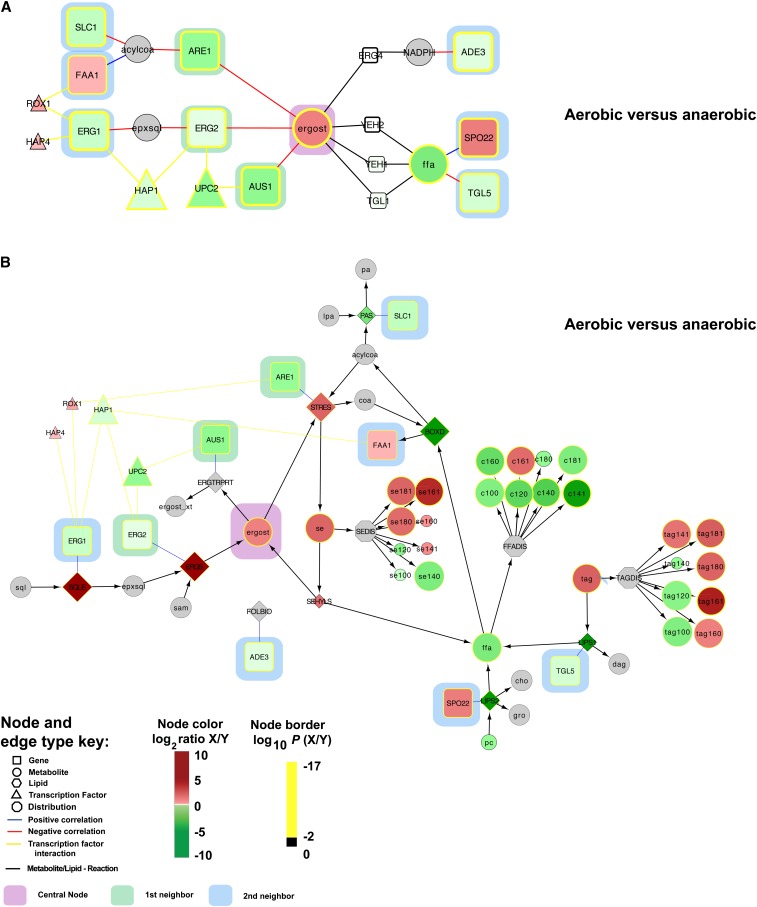

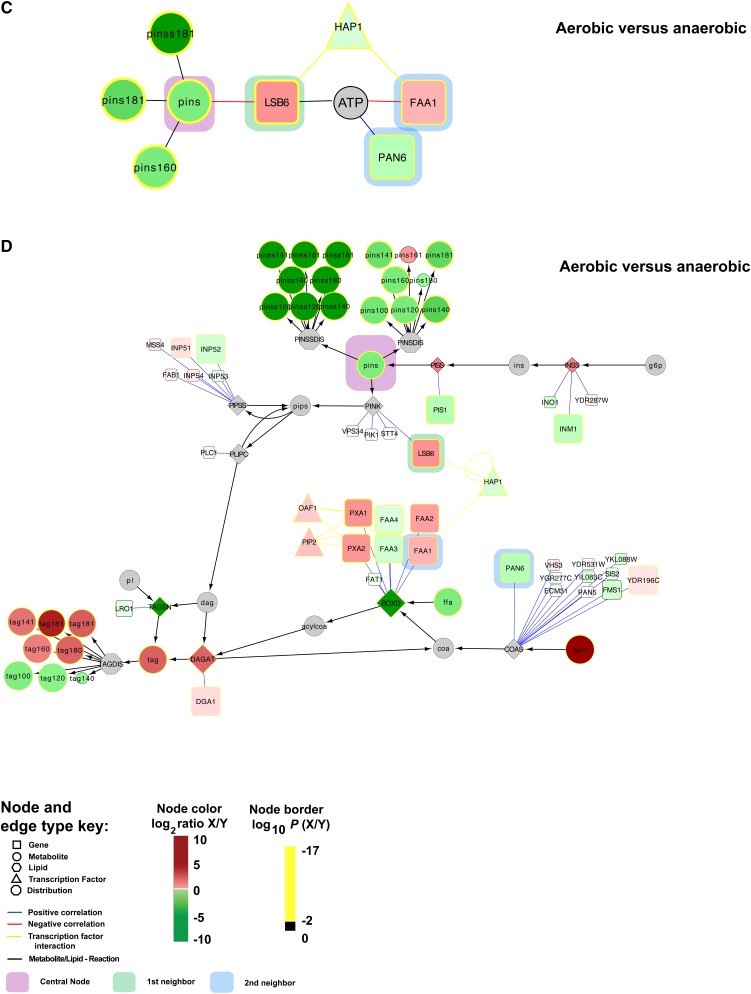
Integrative method for correlation of multi-omics datasets reveals systems-level regulatory signatures. Correlation networks for ergosterol (A and B) and phosphatidylinositol (C and D) show first (green highlight) and second (blue highlight) significantly linked gene neighbors. (A and C) Genes in small white boxes were not identified as significantly correlated to ergosterol or phosphatidylinositol, but they are represented as “connector” nodes between metabolites. TFs implicated by the enrichment analysis are shown. Co-regulated gene neighborhood networks from (A and C) were expanded to include genes and metabolites necessary to perform the metabolic transformations indicated (B and D). This provides a more integrated perspective of cellular regulation. Measurement ratios for aerobic *vs.* anaerobic conditions were visualized with a log_2_ color bar, and the color of each node border represents the log_10_(*P* value). See node and edge color key. Gray indicates the lack of a measurement for that node.

Finally, we built correlation networks for ergosterol and phosphatidylinositol based on the overlap between first and second gene neighbors from the co-regulated gene neighborhoods and the genome-scale metabolic map (Figure 7). The key idea was to identify areas of metabolism that are closely connected with significant and coordinated response to genetic or environmental perturbations. For ergosterol, we identified 76 significant gene expression level–ergosterol interactions in the correlation analysis (Figure S23) and mapped 102 genes linked to ergosterol in the genome scale metabolic model. By taking the intersection of these two groups, we identified nine metabolites that were both linked as a first and second gene neighbors to ergosterol in the metabolic map and were significantly correlated (*e.g.*, ergosterol → *ARE1* → acylCoA → *FAA1*). This topological map was then integrated with the TF-regulated modules implicated in the enrichment analysis to provide a global regulatory picture (Figure 7A). One TF, Upc2p, is known to activate the expression of sterol biosynthetic genes in sterol-depleted cells (Espenshade and Hughes 2007; Gaspar *et al.* 2007) and could be directly linked to Erg2p and Aus1p. The identification of Upc2p as significant when comparing aerobic *vs.* anaerobic conditions validates our undirected approach. Notably, our results suggest that Upc2p functions by controlling the enzyme levels at the transcriptional level and that this results in altered fluxes toward ergosterol. This points to Upc2p playing a similar function as the SREBP-1 transcription factor in mammals (Nielsen 2009; Nookaew *et al.* 2010). Our analysis also identified a significant link between ergosterol and 1-acyl-sn-glycerol-3-phosphate acyltransferase (*SLC1*), which is responsible for the synthesis of phosphatidic acid (PA). PA is the central precursor for glycerophospholipids, DAG, and TAG, and it is also a signaling lipid and key transcriptional regulator of lipid biosynthesis (Carman and Henry 2007). Our correlation network from the co-regulated gene neighborhoods was expanded to provide context within the scope of the genome-scale metabolic map (Figure 7B). Follow-up studies are expected to use the systems interactions here to bring new understanding to lipid metabolism.

For phosphatidylinositol species identified in the co-regulated gene neighborhood analysis (PINS, PINS160, PINS181, PINSS181), we observed that Lsb6p, Faa1p, and Pan6p were significantly correlated and directly linked as first or second neighbors to PINS (Figure 7, C and D). These data suggest that global regulatory points centered at this hub include precursor synthesis (*e.g.*, coenzyme A synthesis, *PAN6*; long chain fatty acyl-CoA synthesis, *FAA1*) and PINS utilization (phosphatidylinsoitol phosphate biosynthesis, *LSB6*). We also analyzed the storage depots of the cell, noting that steryl esters (se161) and triacylglycerol (tag100 and tag140) were significantly correlated and linked in the metabolic network to *PLB2*, *FAA2*, *FAT1*, *LRO1*, *POT1*, and *CAT2* (Figure S24).

In summary, we provide a new integrative method for mapping condition-dependent regulation in cells through the integrated analysis of multi-omics datasets and genome-scale metabolic models given previous knowledge (*e.g.*, known network topology or results from the analysis of other omics datasets such as transcription factor interactions). Specifically, we leveraged a full factorial design to build a visual metabolic model of the regulatory architecture controlling lipid metabolism in *S. cerevisiae*. Simple analysis led to the identification of genetic and metabolic changes involved in TAG and SE accumulation under carbon excess. From an adaptive perspective, yeast and, more broadly, eukaryotes make use of TAG as the major storage unit for energy and fatty acids used in membrane biosynthesis. Our results from growth conditions with high levels of residual glucose are entirely consistent with this phenomena and much is known about the synthesis, turnover, and regulation of nonpolar lipids in yeast (Rajakumari *et al.* 2008). We further revealed a major shift toward the synthesis and utilization of di-substituted medium acyl chain fatty acids under anaerobic conditions and then used ^13^C-reaction flux data to demonstrate this was attributable to redox constraints.

We also developed integrative methods for data analysis that use correlation analysis, metabolic topology, and transcription factor enrichment to interrogate and characterize the complex relationships that arise between gene expression levels, metabolite levels, lipid levels, and reaction fluxes across multiple conditions. Our approach quantitatively revealed that transcriptional regulation is not tightly controlled at the one-step level. Strikingly, only 22 metabolites and lipids were tightly correlated to their first and second gene neighbors. In the case of ergosterol, our analysis identified Upc2p as the key regulator (Espenshade and Hughes 2007; Nielsen 2009). Our systems approach also quantitatively shows that sterol biosynthesis is more regulated at the transcriptional level than amino acid biosynthesis. Looking forward, our work will serve as a rich data resource for studying lipid metabolism, opening the door to new hypothesis-driven targeted experiments. In addition, we expect that it will contribute meaningfully to further efforts to use genome-scale metabolic models for contextualizing information obtained in systems-level data for bridging the gap between transcriptional state and metabolic phenotype.

## Supplementary Material

Supporting Information
