# Supplementary material for: Mapping Condition-Dependent Regulation of Lipid Metabolism in Saccharomyces cerevisiae
Source: G3 (Bethesda). 2013 Nov 1;3(11):1979–95. doi: 10.1534/g3.113.006601 (PMC3815060; doi:10.1534/g3.113.006601)
Supplement: Supporting Information [file supp_3_11_1979__index.html]

Mapping Condition-Dependent Regulation of Lipid Metabolism in Saccharomyces cerevisiae — Supporting Information 

# Mapping Condition-Dependent Regulation of Lipid Metabolism in *Saccharomyces cerevisiae*

## Supporting Information for Jewett *et al.*, 2013

**Files in this Data Supplement:**

- Supporting Information - Figures S1-S24 and Tables S1-S11 (PDF, 2 MB)
- Figure S1 - Pie chart representation of measured lipid classes for each experimental condition based on μmol/gDCW (DCW = dry cell weight). (PDF, 99 KB)
- Figure S2 - Triacylglycerol (A) and steryl ester (B) content for each experimental condition based on μmol/gDCW (dry cell weight). (PDF, 101 KB)
- Figure S3 - Phosphatidylcholine (A) and di-substituted medium acyl-chain phosphatidylcholine (B) content for each experimental condition based on μmol/gDCW (dry cell weight). (PDF, 107 KB)
- Figure S4 - Phosphatidylinositol (A) and di-substituted medium acyl-chain phosphatidylinositol (B) content for each experimental condition based on μmol/gDCW (dry cell weight). (PDF, 101 KB)
- Figure S5 - Phosphatidylserine (A) and phosphatidylethanolamine (B) content for each experimental condition based on μmol/gDCW (dry cell weight). (PDF, 106 KB)
- Figure S6 - Free fatty acids (A) and ergosterol (B) content for each experimental condition based on μmol/gDCW (dry cell weight). (PDF, 101 KB)
- Figure S7 - Total phospholipid (A) and storage lipid (B) content for each experimental condition based on μmol/gDCW (dry cell weight). (PDF, 106 KB)
- Figure S8 - Total fatty acid content in all lipid species for each experimental condition based on μmol/gDCW (dry cell weight). (PDF, 95 KB)
- Figure S9 - Total acyl chain composition from all measured lipid species (phosphatidylinositol, phosphatidylcholine, phosphatidylserine, phosphatidylethanolamine, triacylglycerol, steryl esters, and free fatty acids) for each experimental condition based on μmol/gDCW (dry cell weight). (PDF, 124 KB)
- Figure S10 - The overlap amongst significant genes (A), lipids (B), and metabolites (C) at a threshold of *P* less than or equal to 0.001 following Bonferroni correction across experimental conditions. (PDF, 104 KB)
- Figure S11 - The condition dependent response of the TCA cycle and amino acid biosynthesis comparing C-Limited versus N-limited, 30°C versus 15°C, and aerobic versus anaerobic conditions. (PDF, 1 MB)
- Figure S12 - The condition dependent response of phospholipid biosynthesis comparing C-Limited versus N-limited, 30°C versus 15°C, and aerobic versus anaerobic conditions. (PDF, 287 KB)
- Figure S13 - The condition dependent response of small cellular networks as visualized using Cytoscape. (PDF, 346 KB)
- Figure S14 - The ratio of non-polar lipids (free fatty acids, sterols, and steryl esters) to phospholipids (phosphatidylinositol, phosphatidylcholine, phosphatidylserine, and phosphatidylethanolamine) for each experimental condition based on μmol/gDCW (dry cell weight). (PDF, 103 KB)
- Figure S15 - 13C-flux analysis demonstrates carbon flux through the pentose phosphate pathway is significantly decreased under anaerobic conditions (CAt & CAT relative to COt & COT). (PDF, 118 KB)
- Figure S16 - Network of translation factors enriched for different growth factors. (PDF, 95 KB)
- Figure S17 - Correlation analysis demonstrates significant (*P* less than or equal to 0.001 following Bonferroni correction) relationships between genes and lipids as characterized by length when comparing carbon-limited versus nitrogen-limited conditions. (PDF, 170 KB)
- Figure S18 - Correlation analysis demonstrates significant (*P* less than or equal to 0.001 following Bonferroni correction) relationships between genes and lipids as characterized by length when comparing aerobic "O" versus anaerobic "A" conditions. (PDF, 187 KB)
- Figure S19 - Correlation analysis demonstrates significant (*P* less than or equal to 0.001 following Bonferroni correction) relationships between genes and lipids as characterized by length when comparing high temperature (30°C) versus low temperature (15°C) conditions. (PDF, 138 KB)
- Figure S20 - Correlation analysis demonstrates significant (*P* less than or equal to 0.001 following Bonferroni correction) gene-lipid and gene-metabolite relationships when comparing carbon-limited versus nitrogen-limited conditions. (PDF, 210 KB)
- Figure S21 - Correlation analysis demonstrates significant (*P* less than or equal to 0.001 following Bonferroni correction) gene-lipid and gene-metabolite relationships when comparing aerobic "O" versus anaerobic "A" conditions. (PDF, 186 KB)
- Figure S22 - Correlation analysis demonstrates significant (*P* less than or equal to 0.001 following Bonferroni correction) gene-lipid and gene-metabolite relationships when comparing high temperature (30°C) versus low temperature (15°C) conditions. (PDF, 160 KB)
- Figure S23 - Correlation analysis demonstrates significant gene-ergosterol relationships (*P* less than or equal to 0.01 following Bonferroni correction). (PDF, 132 KB)
- Figure S24 - Integrative method for correlation of omics data reveals global regulatory signatures. (PDF, 154 KB)
- Table S5 - Percent variance captured by each Principle Component (PC) dimension. (PDF, 67 KB)
- Table S8 - Significant lipids, metabolites, and genes when comparing nitrogen-limited aerobic conditions (NOx = NOT & NOt) versus all other conditions (i.e., COT, COt, CAT, CAt, NAT, & NAt) are shown. (PDF, 84 KB)
- Table S9 - Direct connections between genes and lipids or metabolites in the *iIN800* metabolic network and the correlation network. (PDF, 80 KB)
- Table S10 - Based on measured lipids and metabolites that were identified in the correlation analysis, we observed that sterol levels were most highly correlated with 1st and 2nd gene neighbors (*P* less than or equal to 0.01, Benjamini Hochberg *p*-value adjustment). (PDF, 84 KB)
- Table S11 - KEGG pathways whose gene neighbors for sets of metabolites have a bias to be significantly correlated or anti-correlated. (PDF, 75 KB)
- Table S1 - Metabolic reconstruction of *iIN800*. (.xlsx, 212 KB)
- Table S2 - Normalized mRNA, metabolite, and lipid data. (.xlsx, 865 KB)
- Table S3 - Physiological yield data and steady-state nutrient concentrations in chemostat cultures. (.xls, 45 KB)
- Table S4 - Multi-way ANOVA results for single factors: carbon-limited vs nitrogen-limited, "CN"; aerobic vs anaerobic, "OA"; and 30C vs 15C "Tt"; with associated log2-fold-change (LFC) and p-values. (.xlsx, 1 MB)
- Table S6 - Metabolic model for cytoscape visualization used in this study. (.xlsx, 51 KB)
- Table S7 - *In silico* fluxes under the constraints of maximized biomass production, a steady state metabolic network, and fixed protein composition. (.xlsx, 58 KB)
