# Supplementary material for: Mapping Condition-Dependent Regulation of Lipid Metabolism in Saccharomyces cerevisiae
Source: G3 (Bethesda). 2013 Nov 1;3(11):1979–95. doi: 10.1534/g3.113.006601 (PMC3815060; doi:10.1534/g3.113.006601)
Supplement: Supporting Information [file supp_g3.113.006601_006601SI.pdf]

G3: Genes | Genomes | Genetics

Mapping Condition Dependent Regulation of Lipid Metabolism in *Saccharomyces cerevisiae*

Michael C. Jewett[*,§,1], Christopher T. Workman[**,1], Intawat Nookaew[*,§§,†], Francisco A. Pizarro[*,‡], Eduardo Agosin[*,‡], Lars I. Hellgren[**], Jens Nielsen[*, §§, 2]

[*] *Center for Microbial Biotechnology, DTU Systems Biology, Technical University of Denmark, Søltofts Plads, Building 223, DK-2800 Kgs. Lyngby, Denmark.*
[§] *Department of Chemical and Biological Engineering and Chemistry of Life Processes Institute Northwestern University, 2145 Sheridan Road, Evanston, IL 60208, USA*
[**] *Center for Biological Sequence Analysis, DTU Systems Biology, Technical University of Denmark, Building 208, DK-2800 Kgs. Lyngby, Denmark.*
[§§] *Department of Chemical and Biological Engineering, Chalmers University of Technology, Kemivägen 10, SE-412 96 Göteborg, Sweden*
[†] *Faculty of Engineering, King Mongkut's University of Technology Thonburi, Bangkok 10140, Thailand*
[‡] *Department of Chemical and Bioprocess Engineering, School of Engineering, Pontificia Universidad Católica de Chile, Av. Vicuña Mackenna 4860, Macul, Santiago, Chile.*

[1] *These authors contributed equally*
[2] *To whom correspondence should be addressed.* Department of Chemical and Biological Engineering, Chalmers University of Technology, Kemivägen 10, SE-412 96 Göteborg, Sweden *E-mail:* nielsenj@chalmers.se

DOI: 10.1534/g3.113.006601

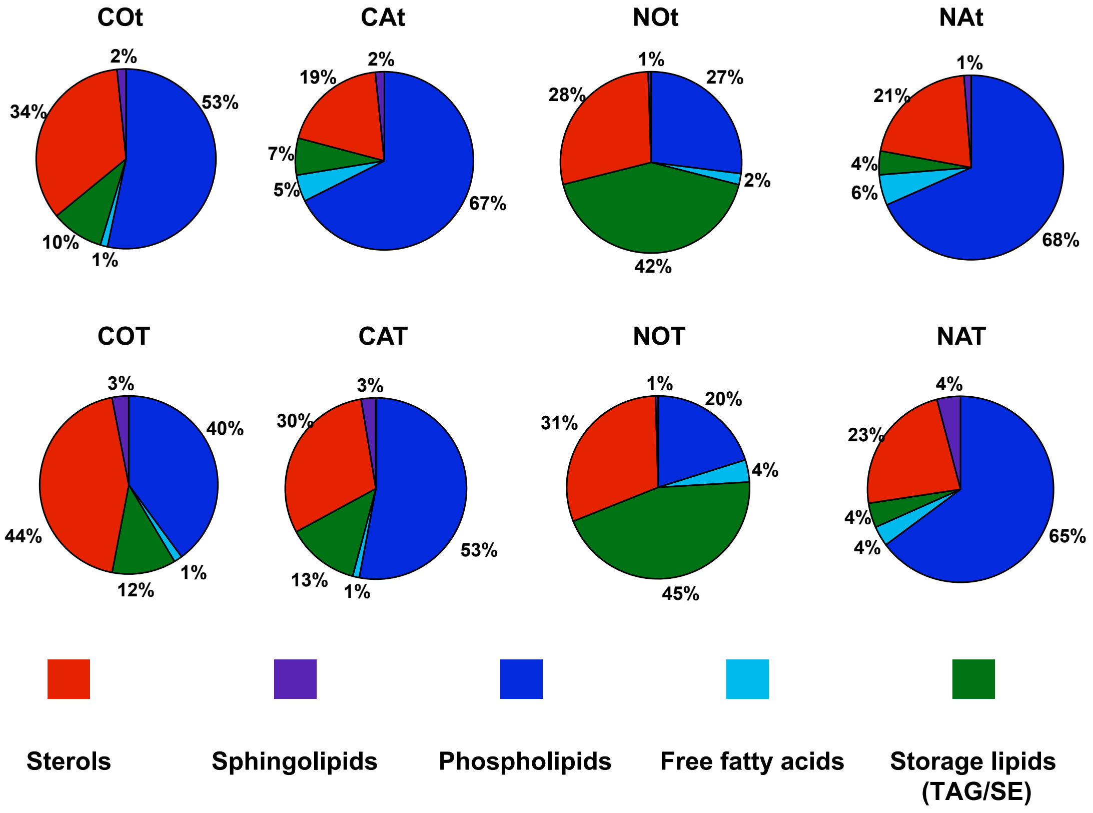

Figure S1 Pie chart representation of measured lipid classes for each experimental condition based on μmol/gDCW (DCW = dry cell weight). Each experiment is given a three letter code (C-limited, "C"; N-limited, "N"; aerobic, "O"; anaerobic, "A"; 30°C, "T"; and 15°C, "t"). For example, "COt" stands for C-limited, aerobic, and 15°C. These data highlight major changes across different conditions. In one example, nitrogen-limited aerobic conditions (NOt & NOT) have the largest percentage of storage lipids in the total lipid pool (42-45%). TAG: triacylglycerol, SE: steryl ester.

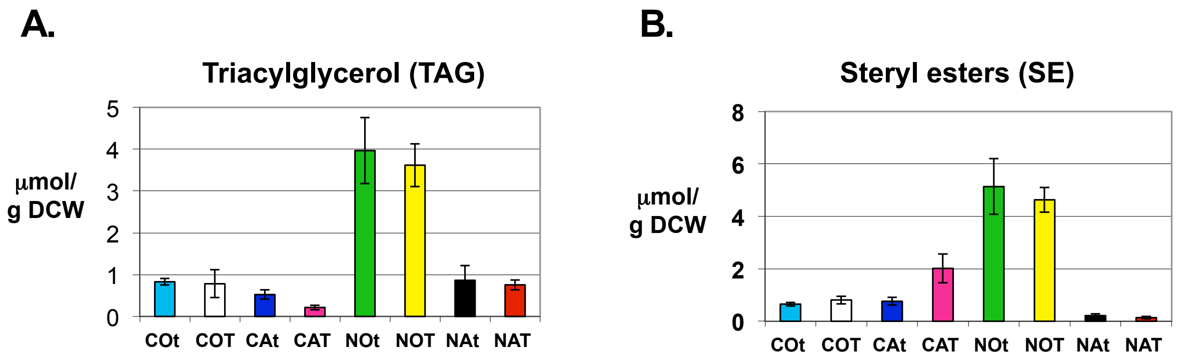

Figure S2 Triacylglycerol (A) and steryl ester (B) content for each experimental condition based on μmol/gDCW (dry cell weight). Each experiment is given a three letter code (C-limited, "C"; N-limited, "N"; aerobic, "O"; anaerobic, "A"; 30°C, "T"; and 15°C, "t"). The content of TAG and SE is increased under nitrogen-limited aerobic conditions (NOt/NOT).

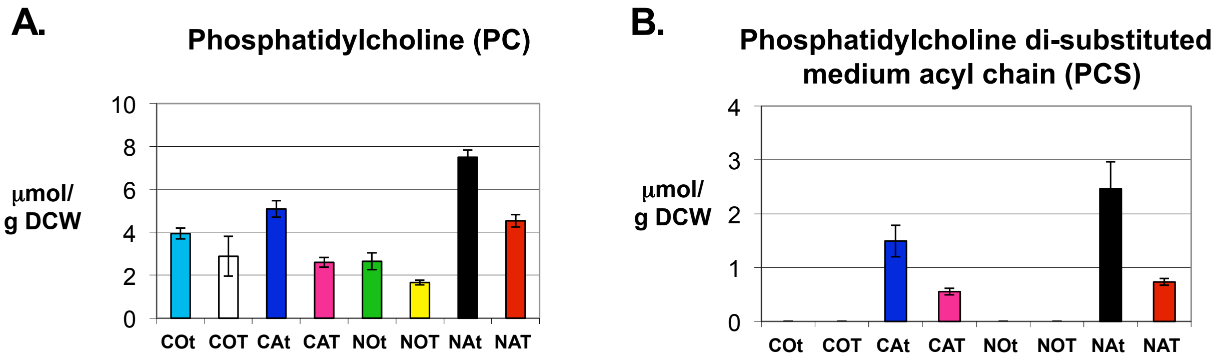

Figure S3 Phosphatidylcholine (A) and di-substituted medium acyl-chain phosphatidylcholine (B) content for each experimental condition based on μmol/gDCW (dry cell weight). Each experiment is given a three letter code (C-limited, "C"; N-limited, "N"; aerobic, "O"; anaerobic, "A"; 30°C, "T"; and 15°C, "t"). Di-substituted medium acyl-chain phosphatidylcholine is only present under anaerobic conditions.

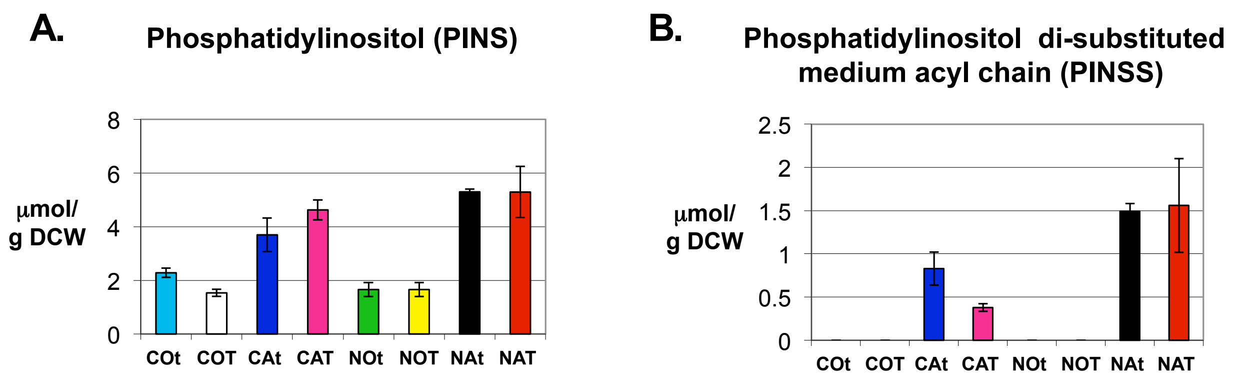

Figure S4 Phosphatidylinositol (A) and di-substituted medium acyl-chain phosphatidylinositol (B) content for each experimental condition based on μmol/gDCW (dry cell weight). Each experiment is given a three letter code (C-limited, "C"; N-limited, "N"; aerobic, "O"; anaerobic, "A"; 30°C, "T"; and 15°C, "t"). Di-substituted medium acyl-chain phosphatidylinositol is only present under anaerobic conditions.

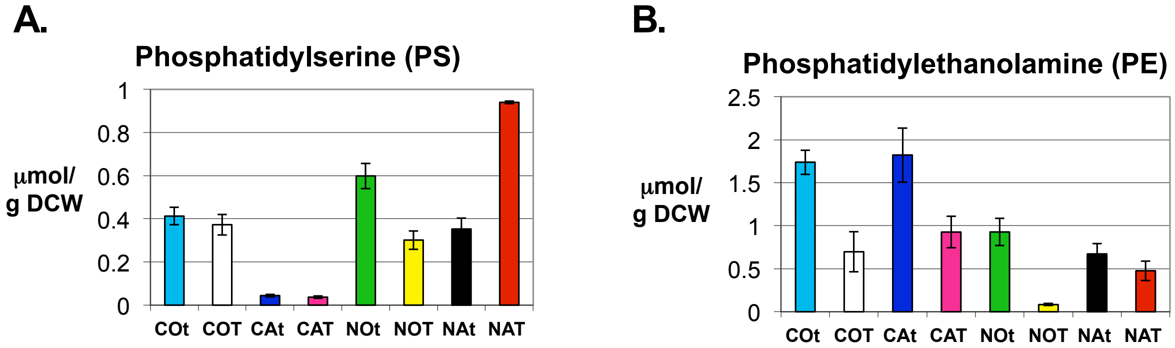

Figure S5 Phosphatidylserine (A) and phosphatidylethanolamine (B) content for each experimental condition based on µmol/gDCW (dry cell weight). Each experiment is given a three letter code (C-limited, "C"; N-limited, "N"; aerobic, "O"; anaerobic, "A"; 30°C, "T"; and 15°C, "t"). Phosphatidylethanolamine increases at lower temperatures.

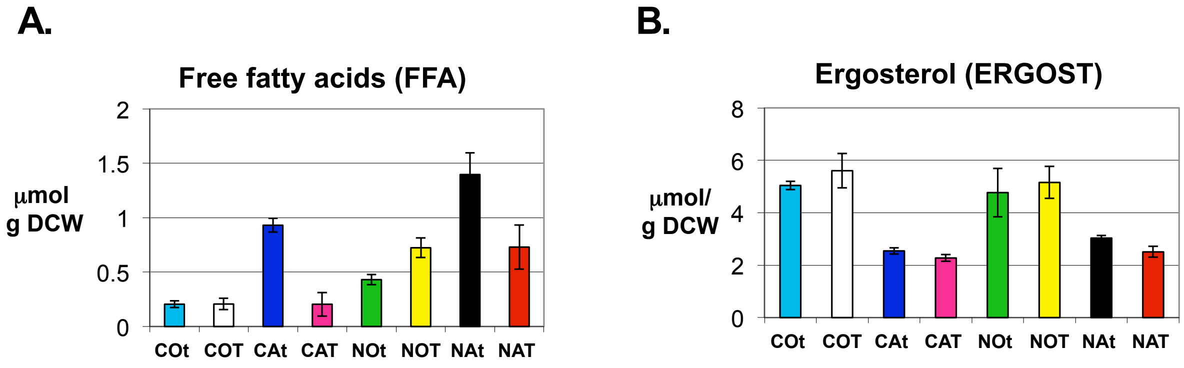

Figure S6 Free fatty acids (A) and ergosterol (B) content for each experimental condition based on µmol/gDCW (dry cell weight). Each experiment is given a three letter code (C-limited, "C"; N-limited, "N"; aerobic, "O"; anaerobic, "A"; 30°C, "T"; and 15°C, "t"). The concentration of free fatty acid is increased under anaerobic conditions, low temperature. Ergosterol levels are significantly higher under aerobic conditions, as expected since ergosterol biosynthesis is not active without oxygen. Note that anaerobic ergosterol levels represent amounts absorbed from the medium.

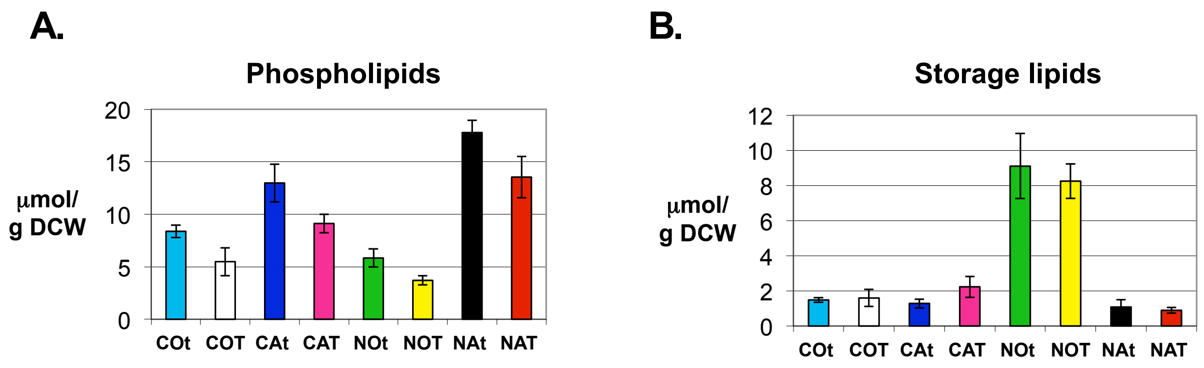

Figure S7 Total phospholipid (A) and storage lipid (B) content for each experimental condition based on μmol/gDCW (dry cell weight). Each experiment is given a three letter code (C-limited, "C"; N-limited, "N"; aerobic, "O"; anaerobic, "A"; 30°C, "T"; and 15°C, "t"). Total phospholipid content (phosphatidylinositol, phosphatidylcholine, phosphatidylserine, and phosphatidylethanolamine) is most dramatically regulated by temperature and oxygen availability. Storage lipids (triacylglycerol and steryl esters) demonstrate a major change in metabolism under N-limited, aerobic conditions.

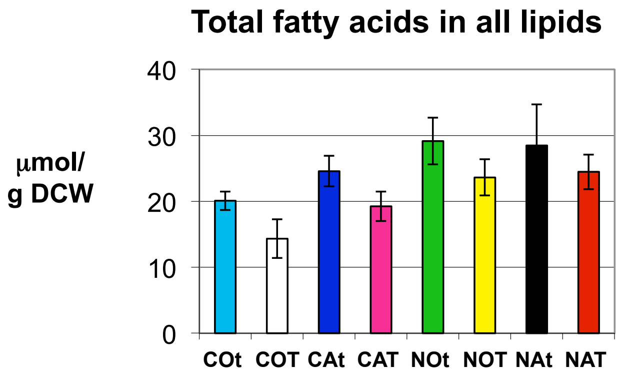

Figure S8 Total fatty acid content in all lipid species for each experimental condition based on μmol/gDCW (dry cell weight). Each experiment is given a three letter code (C-limited, "C"; N-limited, "N"; aerobic, "O"; anaerobic, "A"; 30°C, "T"; and 15°C, "t"). Total fatty acid content is increased at low temperature and under nitrogen-limitation.

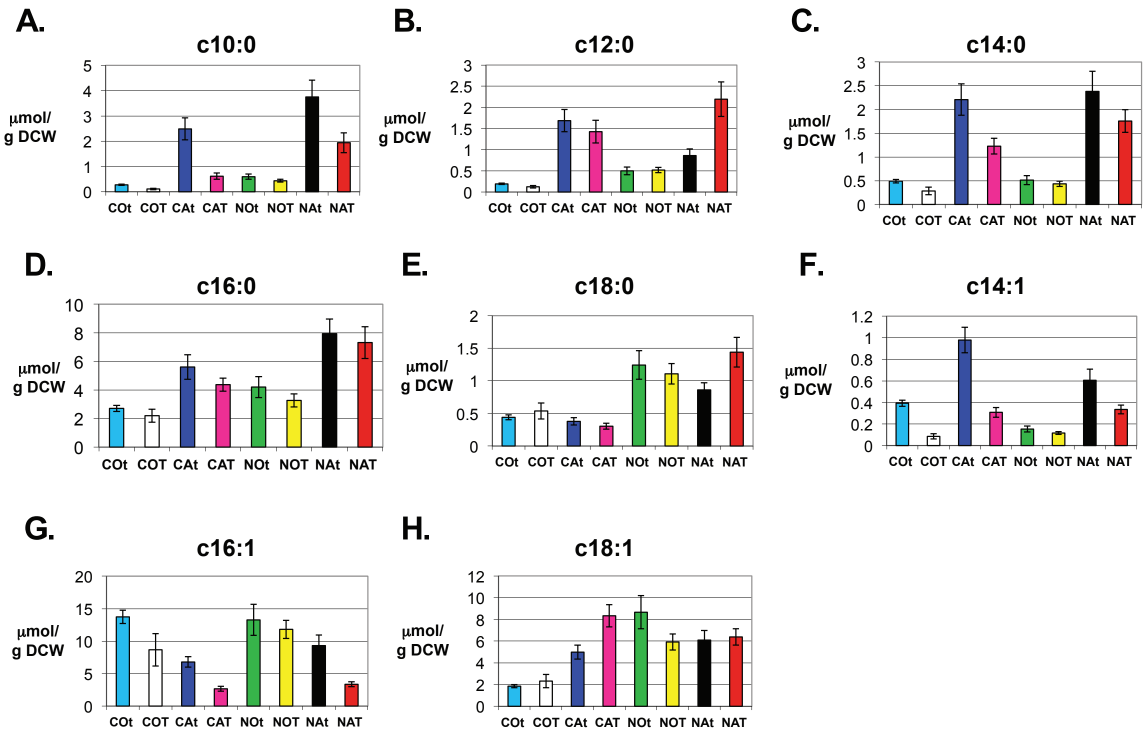

Figure S9 Total acyl chain composition from all measured lipid species (phosphatidylinositol, phosphatidylcholine, phosphatidylserine, phosphatidylethanolamine, triacylglycerol, steryl esters, and free fatty acids) for each experimental condition based on μmol/gDCW (dry cell weight). Each experiment is given a three letter code (C-limited, "C"; N-limited, "N"; aerobic, "O"; anaerobic, "A"; 30°C, "T"; and 15°C, "t"). The notation for acyl-chain length is given as c10:0, where the first number (in this case "10") is the number of carbon atoms and the second number (in this case "0") is the number of double bonds. Key observations include: medium chain acyl groups (C10:0, C12:0, and C14:0) are greater under anaerobic conditions, C16:0 is greater under anaerobic conditions, C18:0 is greater under nitrogen limitation, C14:1 and C16:1 are greater at lower temperatures where both a decreased chain length and the cis double bond will reduce the interaction between the acyl chains, and C18:1 is generally greater under nitrogen limitation.

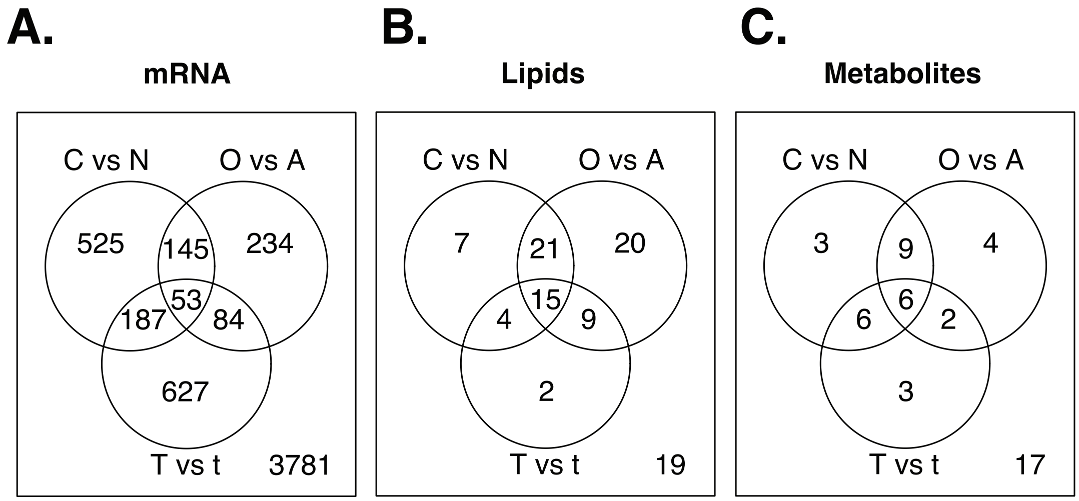

Figure S10 The overlap amongst significant genes (A), lipids (B), and metabolites (C) at a threshold of $P \leq 0.001$ following Bonferroni correction across experimental conditions. C-limited, "C"; N-limited, "N"; aerobic, "O"; anaerobic, "A"; 30°C, "T"; and 15°C, "t". See Table S4 for the list of genes from the Multi-way ANOVA.

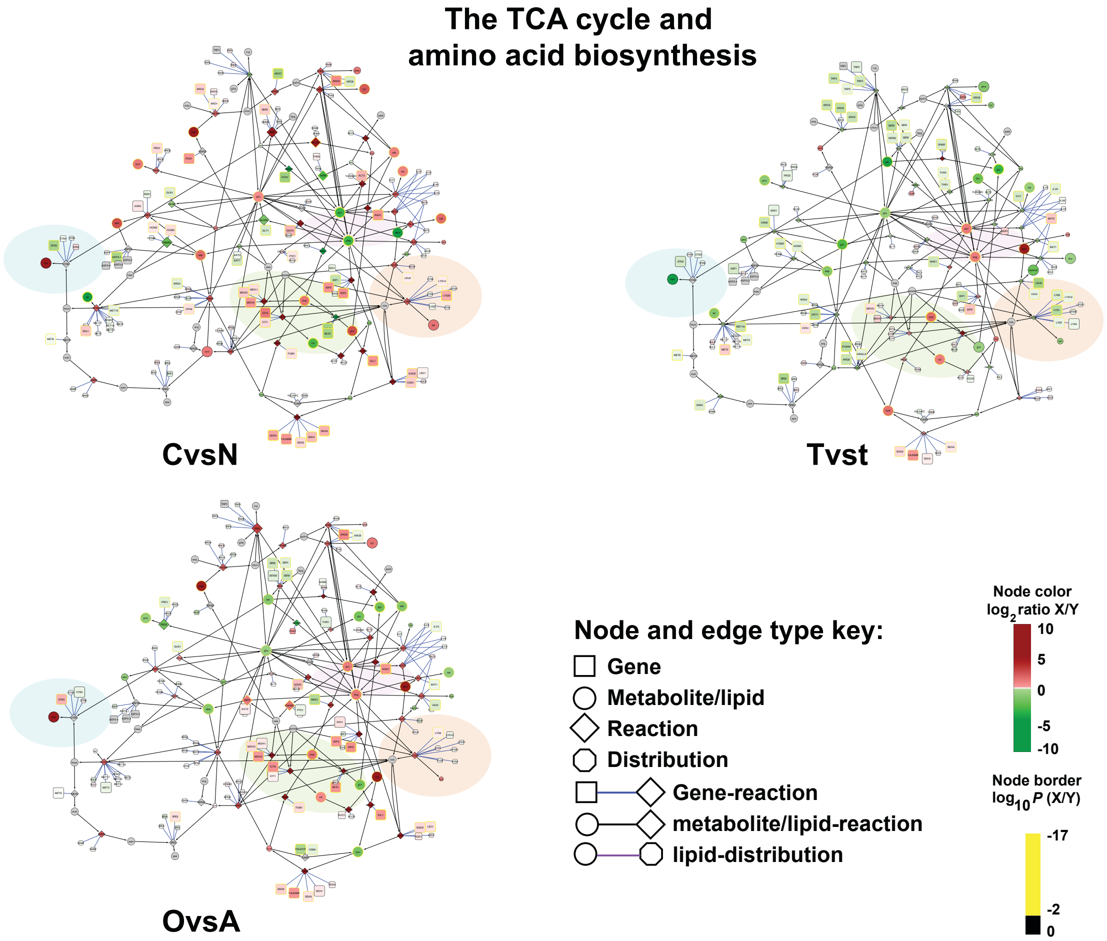

Figure S11 The condition dependent response of the TCA cycle and amino acid biosynthesis comparing C-Limited versus N-limited, 30°C versus 15°C, and aerobic versus anaerobic conditions. The areas highlighted show different up- and down-regulation patterns illustrating how Cytoscape visualization enables the targeted and rapid identification of changes across the cell. C-limited, “C”; N-limited, “N”; aerobic, “O”; anaerobic, “A”; 30°C, “T”; and 15°C, “t”. Measurement ratios were visualized with a $\log_2$ color-bar and the color of each node border represents the $\log_{10}$(*p*-value) (see node and edge color key). Gray coloring indicates the lack of a measurement for that node.

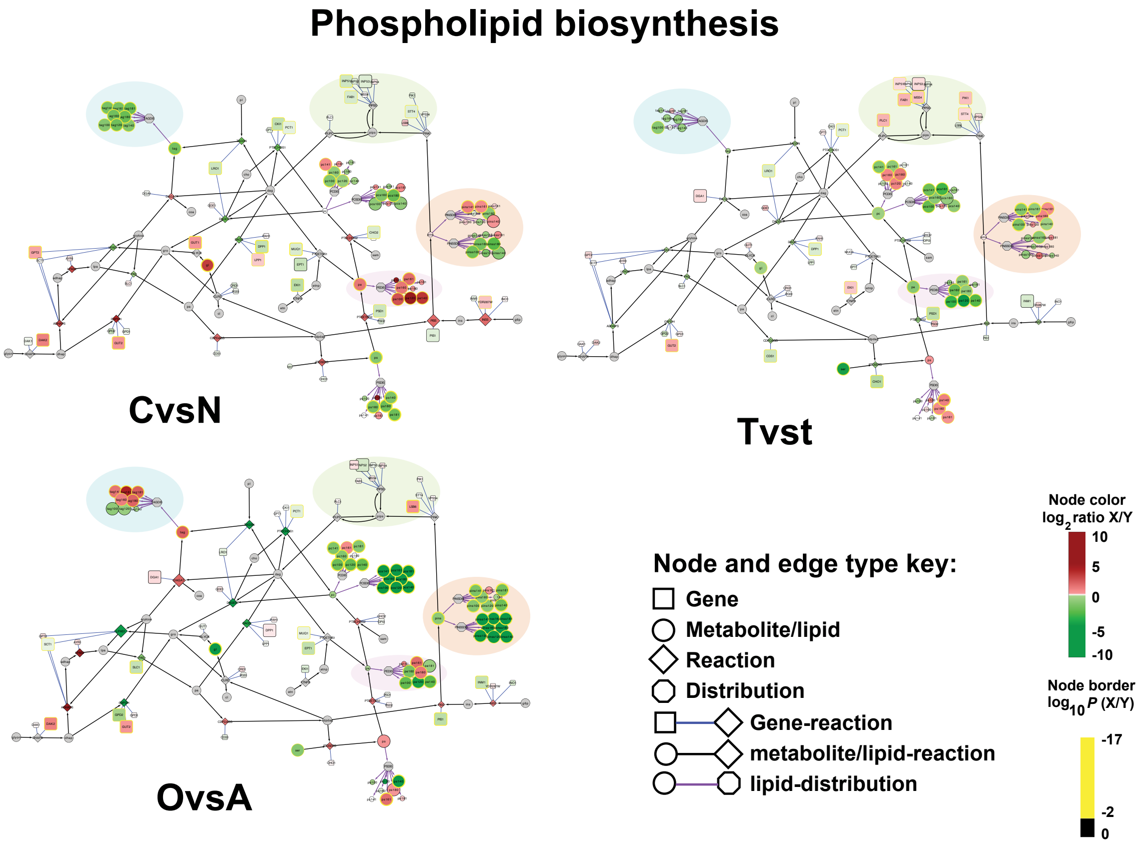

Figure S12 The condition dependent response of phospholipid biosynthesis comparing C-Limited versus N-limited, 30°C versus 15°C, and aerobic versus anaerobic conditions. The areas highlighted show different up- and down- regulation patterns illustrating how Cytoscape visualization enables the targeted and rapid identification of changes across the cell. C-limited, "C"; N-limited, "N"; aerobic, "O"; anaerobic, "A"; 30°C, "T"; and 15°C, "t". Measurement ratios were visualized with a log_2 color-bar and the color of each node border represents the log_{10}(p-value) (see node and edge color key). Gray coloring indicates the lack of a measurement for that node.

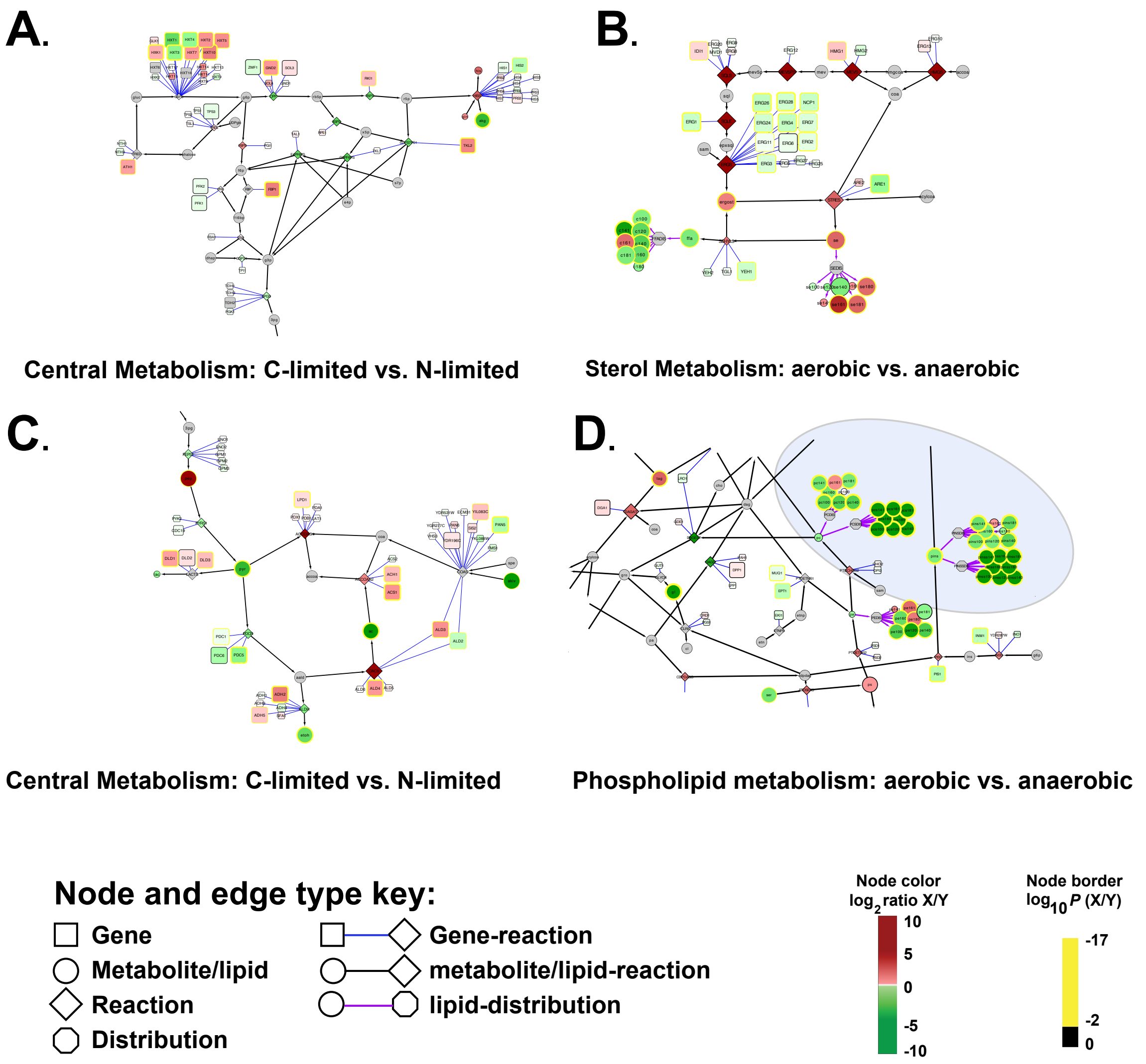
A.
Central Metabolism: C-limited vs. N-limited
B.
Sterol Metabolism: aerobic vs. anaerobic
C.
Central Metabolism: C-limited vs. N-limited
D.
Phospholipid metabolism: aerobic vs. anaerobic
Node and edge type key:
Gene
Metabolite/lipid
Reaction
Distribution
Gene-reaction
metabolite/lipid-reaction
lipid-distribution
Node color
log2 ratio X/Y
10
5
0
-5
-10
Node border
log10 P (X/Y)
-17
-2
0

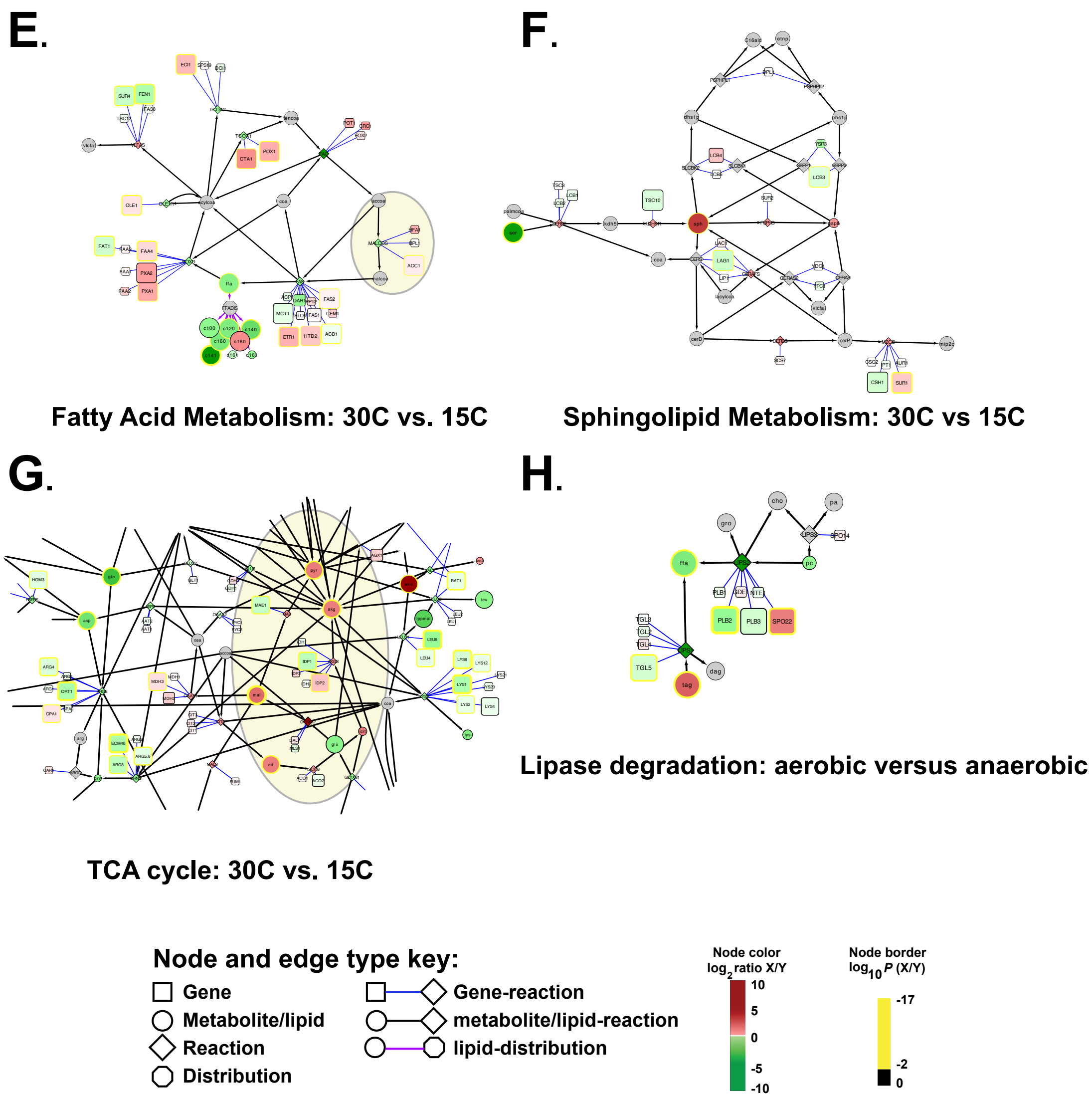

Figure S13 The condition dependent response of small cellular networks as visualized using Cytoscape. (A) Central metabolism: glycolysis, CvsN. (B) Sterol metabolism: OvsA. (C) Central metabolism: pyruvate metabolism, CvsN. (D) Phospholipid metabolism: OvsA. (E) Fatty acid metabolism: Tvst. (F) Sphingolipid metabolism: Tvst. (G) Central metabolism: TCA cycle, Tvst. G. Lipase metabolism: OvsA. C-limited, "C"; N-limited, "N"; aerobic, "O"; anaerobic, "A"; 30°C, "T"; and 15°C, "t". Measurement ratios were visualized with a $\log_2$ color-bar and the color of each node border represents the $\log_{10}$(p-value) (see node and edge color key). Gray coloring indicates the lack of a measurement for that node.

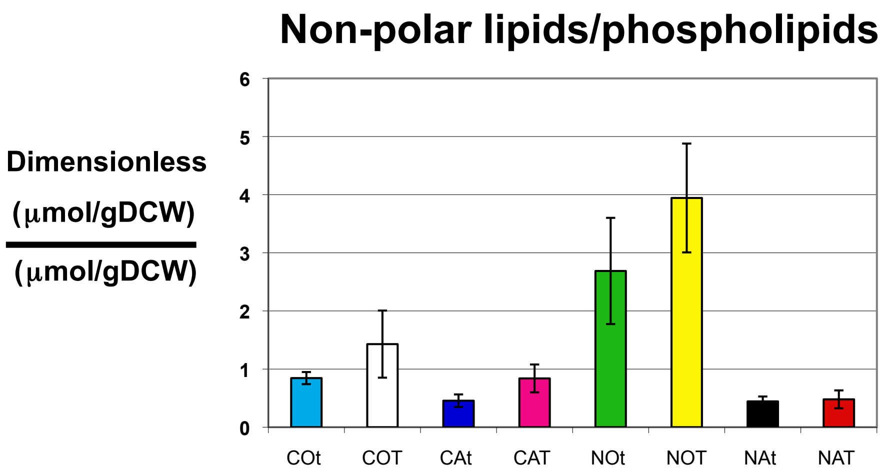

Figure S14 The ratio of non-polar lipids (free fatty acids, sterols, and steryl esters) to phospholipids (phosphatidylinositol, phosphatidylcholine, phosphatidylserine, and phosphatidylethanolamine) for each experimental condition based on μmol/gDCW (dry cell weight). Each experiment is given a three letter code (C-limited, "C"; N-limited, "N"; aerobic, "O"; anaerobic, "A"; 30°C, "T"; and 15°C, "t"). There is a shift away from phospholipid synthesis under nitrogen-limited aerobic conditions (*i.e.,* higher ratio above).

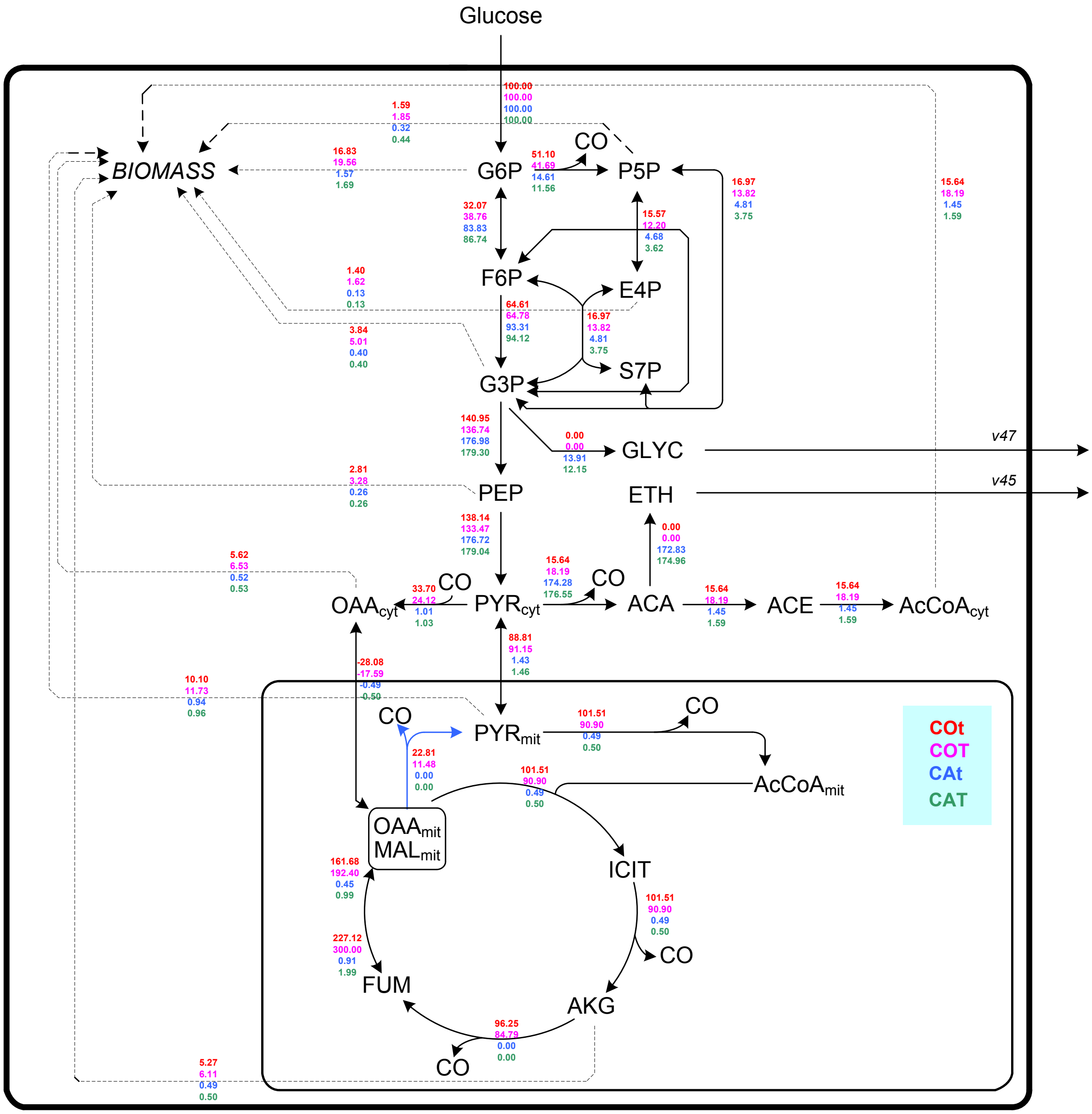

Figure S15 ^{13}C-flux analysis demonstrates carbon flux through the pentose phosphate pathway is significantly decreased under anaerobic conditions (CAt & CAT relative to COt & COT)**.** For example, flux from glucose-6-phosphate (G6P) to pentose-5-phosphate is approximately 3.5-fold higher in the aerobic case. All abbreviations are directly taken from Gombert et al., 2001. Carbon fluxes, measured here, are normalized to glucose uptake. Each experiment is given a three letter code (C-limited, "C"; N-limited, "N"; aerobic, "O"; anaerobic, "A"; 30°C, "T"; and 15°C, "t").

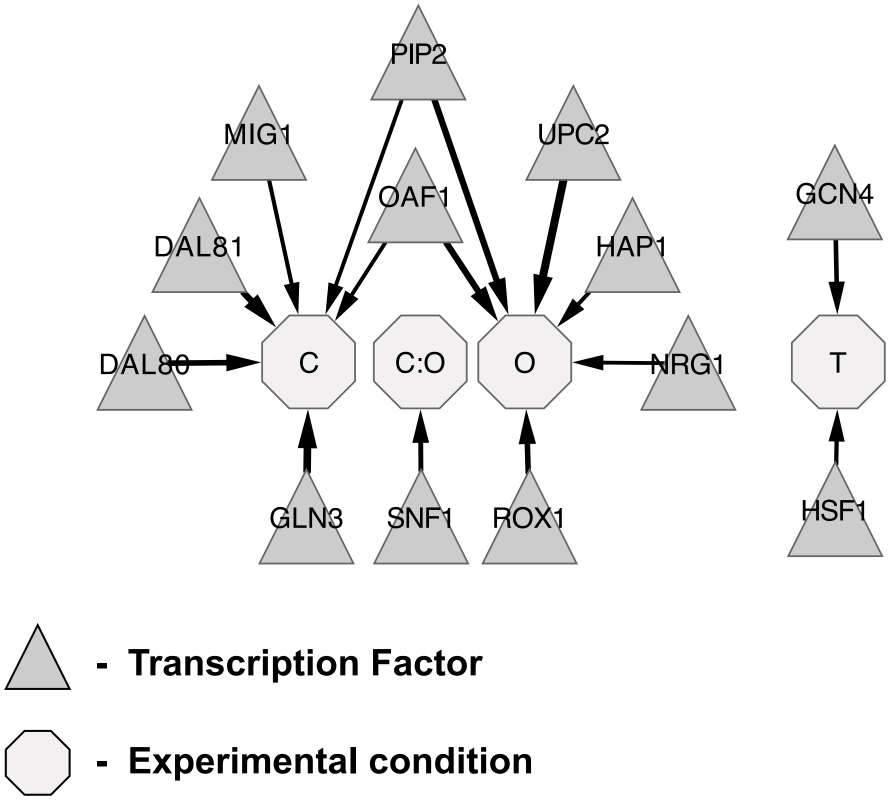

Figure S16 Network of translation factors enriched for different growth factors. Edge thickness represents the number of known transcription factor regulatory targets observed in the experimental condition (with thicker lines indicated more genes) (C-limited, “C”; N-limited, “N”; aerobic, “O”; anaerobic, “A”; 30°C, “T”; and 15°C, “t”).

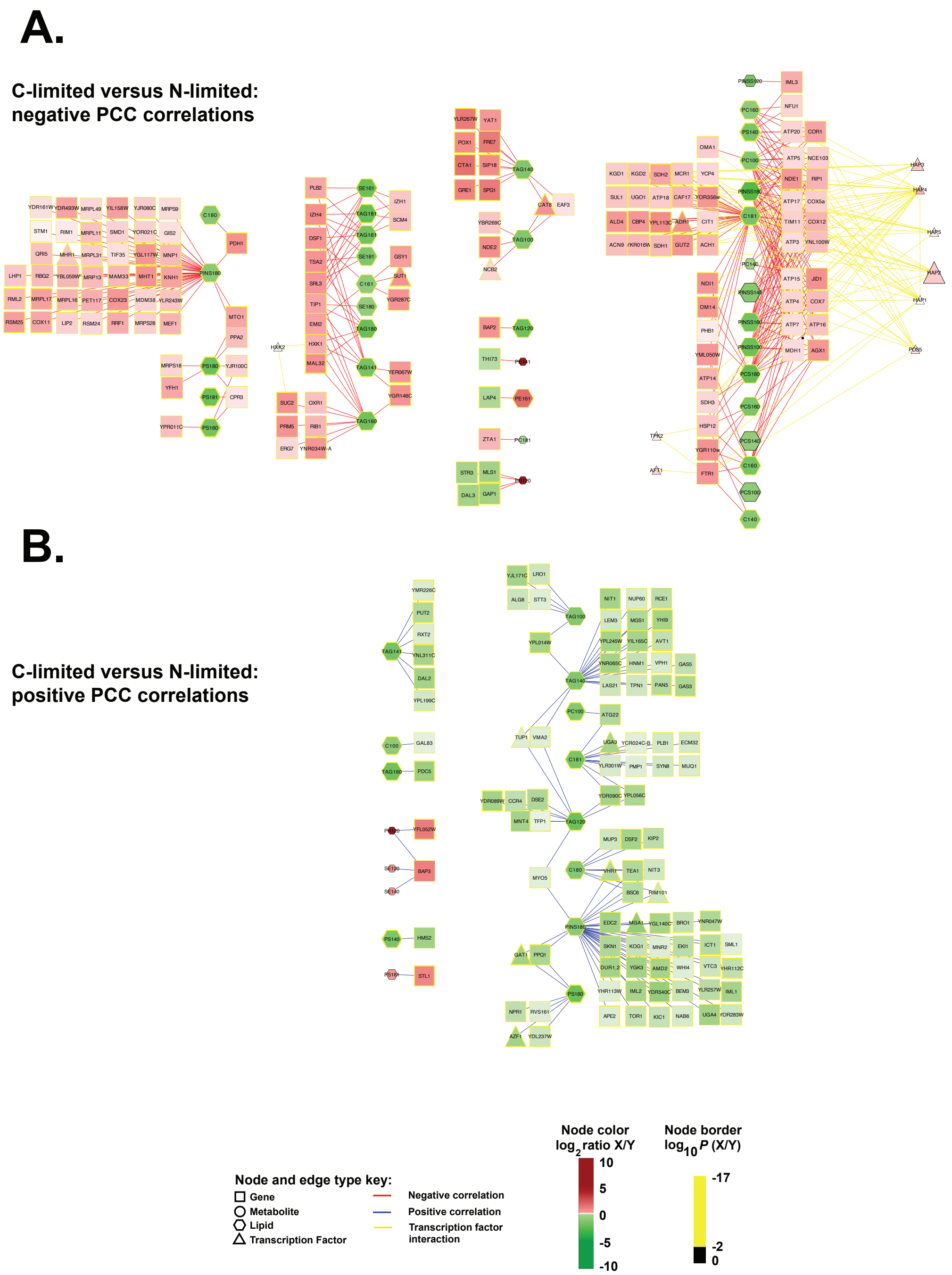

Figure S17 Correlation analysis demonstrates significant ($P \leq 0.001$ following Bonferroni correction) relationships between genes and lipids as characterized by length when comparing carbon-limited versus nitrogen-limited conditions. (A) Negative Pearson Correlation Coefficients (PCC). (B) Positive Pearson Correlation Coefficients (PCC). For example, C18:0 is negatively correlated to *PDH1*. Enriched transcription factors are shown (yellow edges). Measurement ratios were visualized with a $\log_2$ color-bar and the color of each node border represents the $\log_{10}$(p-value) (see node and edge color key).

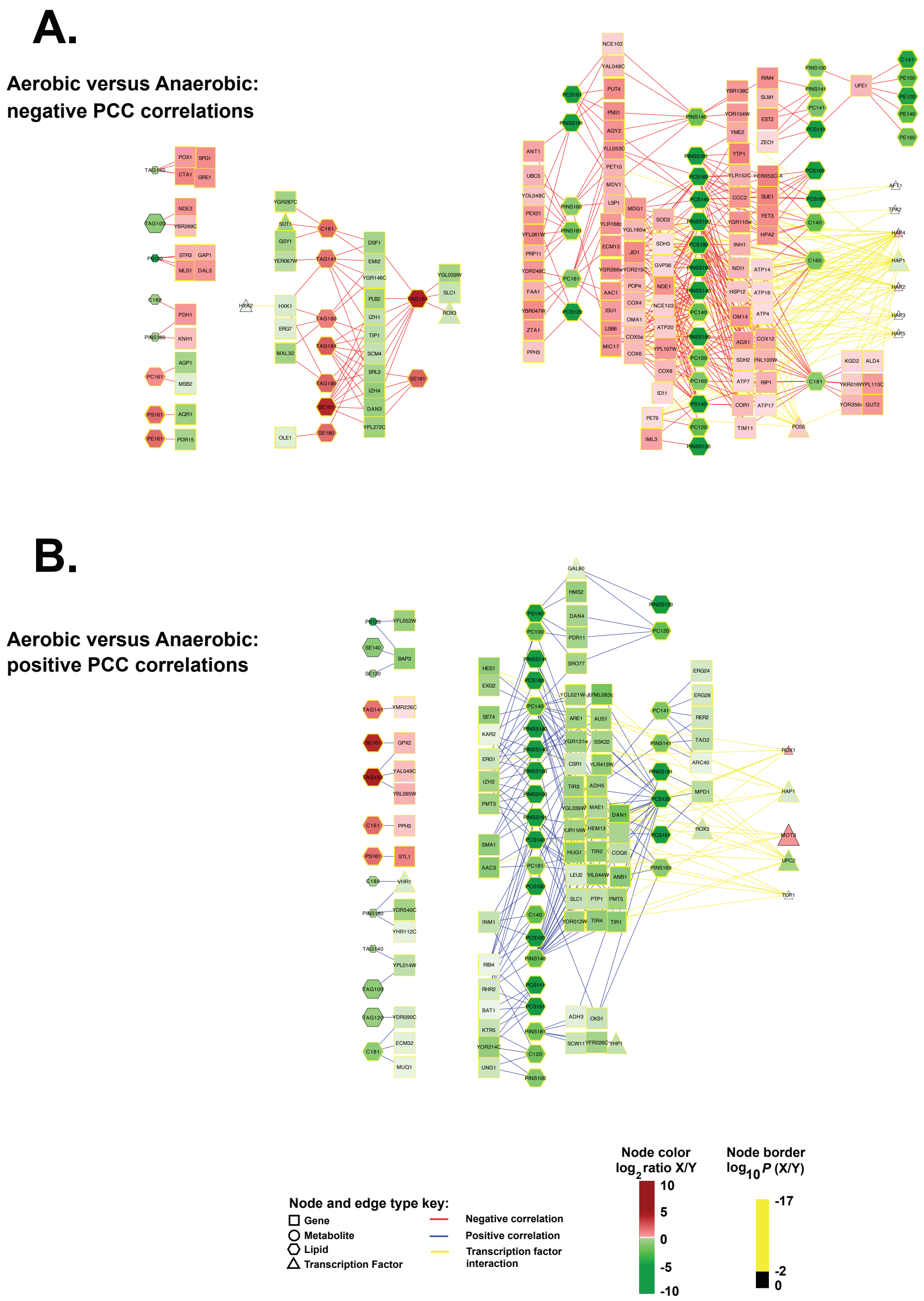

Figure S18 Correlation analysis demonstrates significant ($P \leq 0.001$ following Bonferroni correction) relationships between genes and lipids as characterized by length when comparing aerobic "O" versus anaerobic "A" conditions. (A) Negative Pearson Correlation Coefficients (PCC). (B) Positive Pearson Correlation Coefficients (PCC). Enriched transcription factors are shown (yellow edges). Measurement ratios were visualized with a $\log_2$ color-bar and the color of each node border represents the $\log_{10}$(p-value) (see node and edge color key).

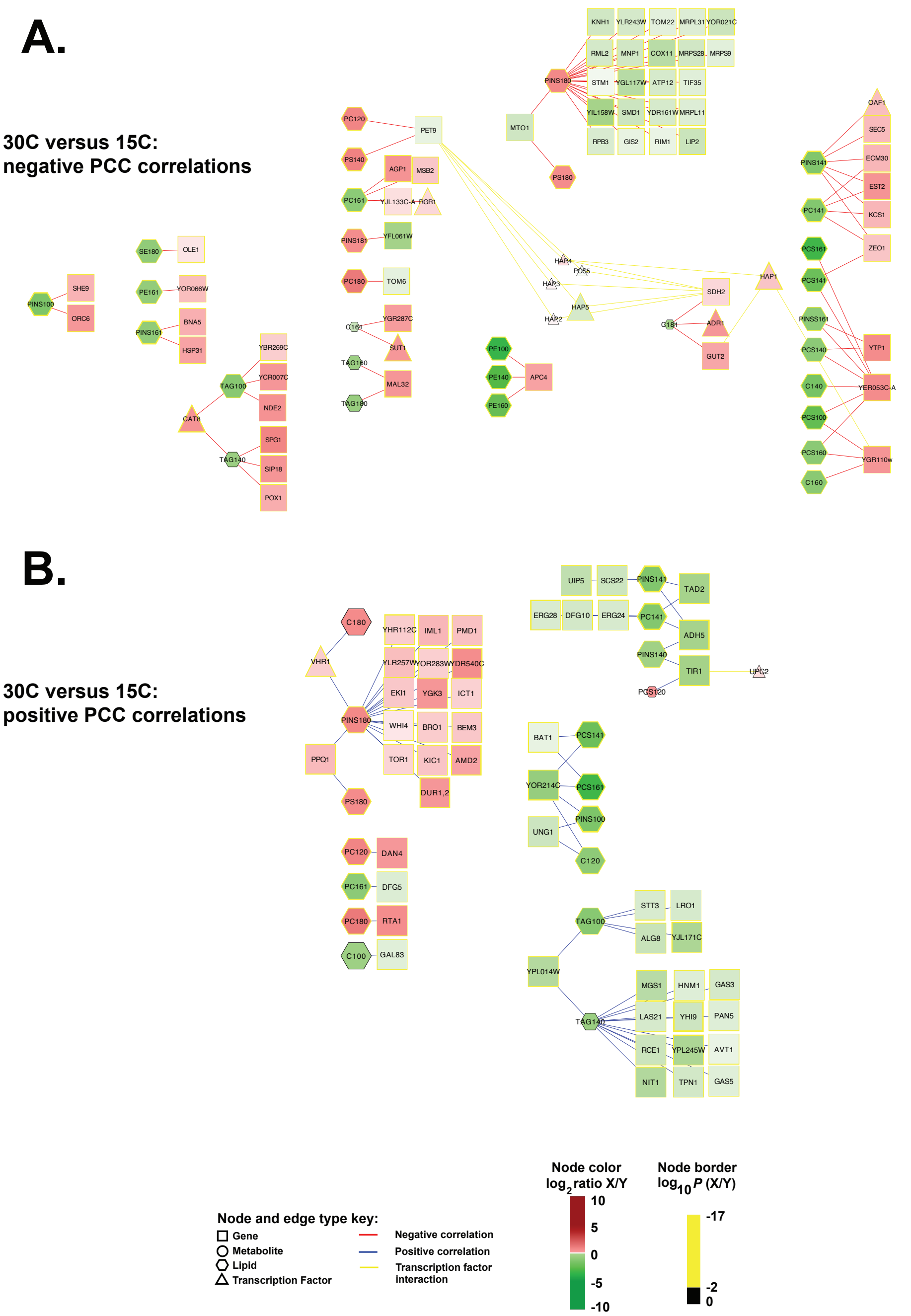

Figure S19 Correlation analysis demonstrates significant ($P \leq 0.001$ following Bonferroni correction) relationships between genes and lipids as characterized by length when comparing high temperature (30°C) versus low temperature (15°C) conditions. (A) Negative Pearson Correlation Coefficients (PCC). (B) Positive Pearson Correlation Coefficients (PCC). Enriched transcription factors are shown (yellow edges). Measurement ratios were visualized with a $\log_2$ color-bar and the color of each node border represents the $\log_{10}$(p-value) (see node and edge color key).

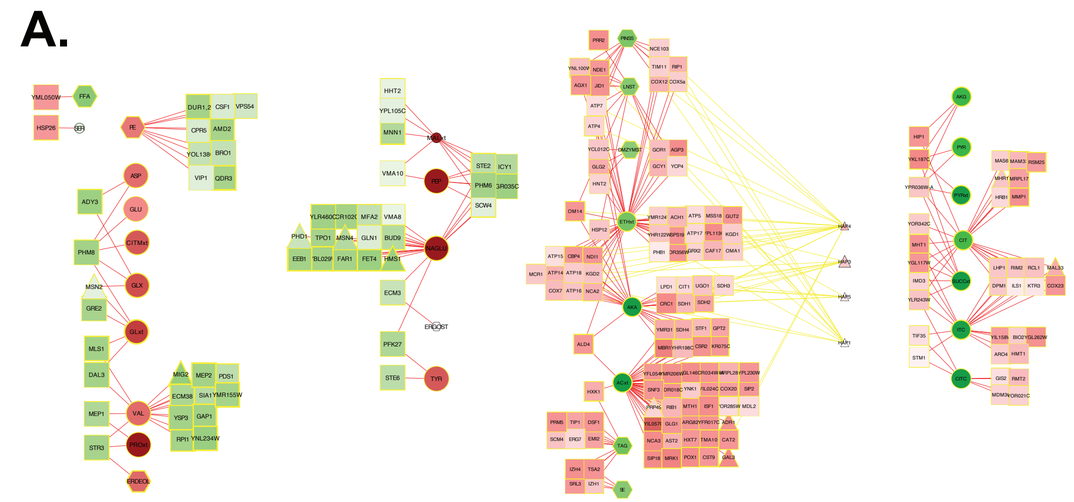

C-limited versus N-limited: negative PCC correlations

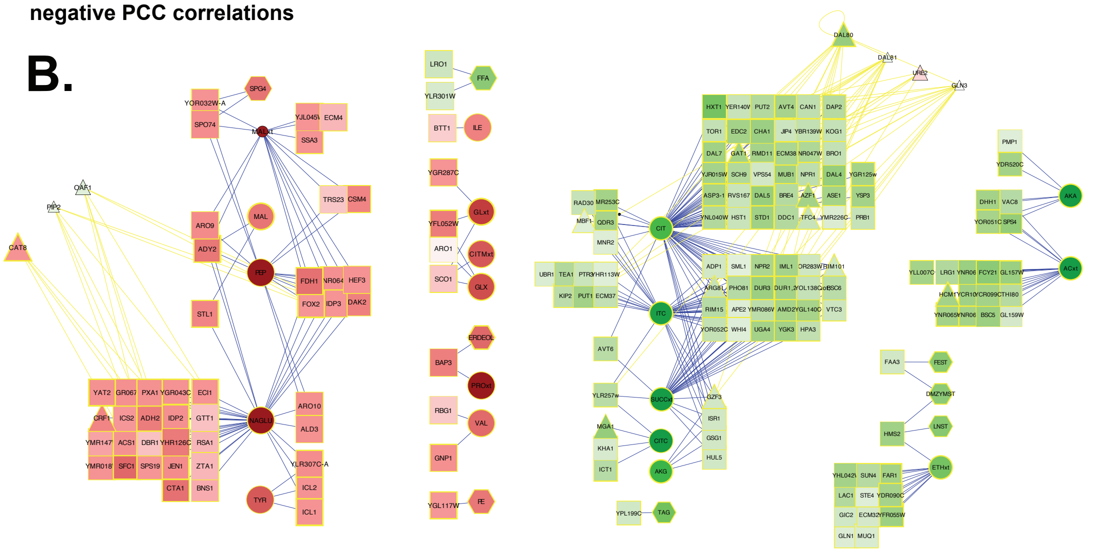

C-limited versus N-limited: positive PCC correlations

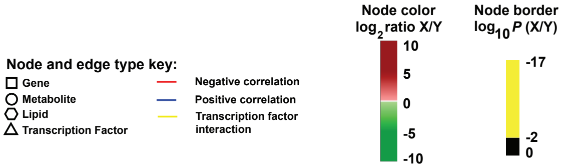

Figure S20 Correlation analysis demonstrates significant ($P \leq 0.001$ following Bonferroni correction) gene-lipid and gene-metabolite relationships when comparing carbon-limited versus nitrogen-limited conditions. (A) Negative Pearson Correlation Coefficients (PCC). (B) Positive Pearson Correlation Coefficients (PCC). Enriched transcription factors are shown (yellow edges). Measurement ratios were visualized with a $\log_2$ color-bar and the color of each node border represents the $\log_{10}$(p-value) (see node and edge color key).

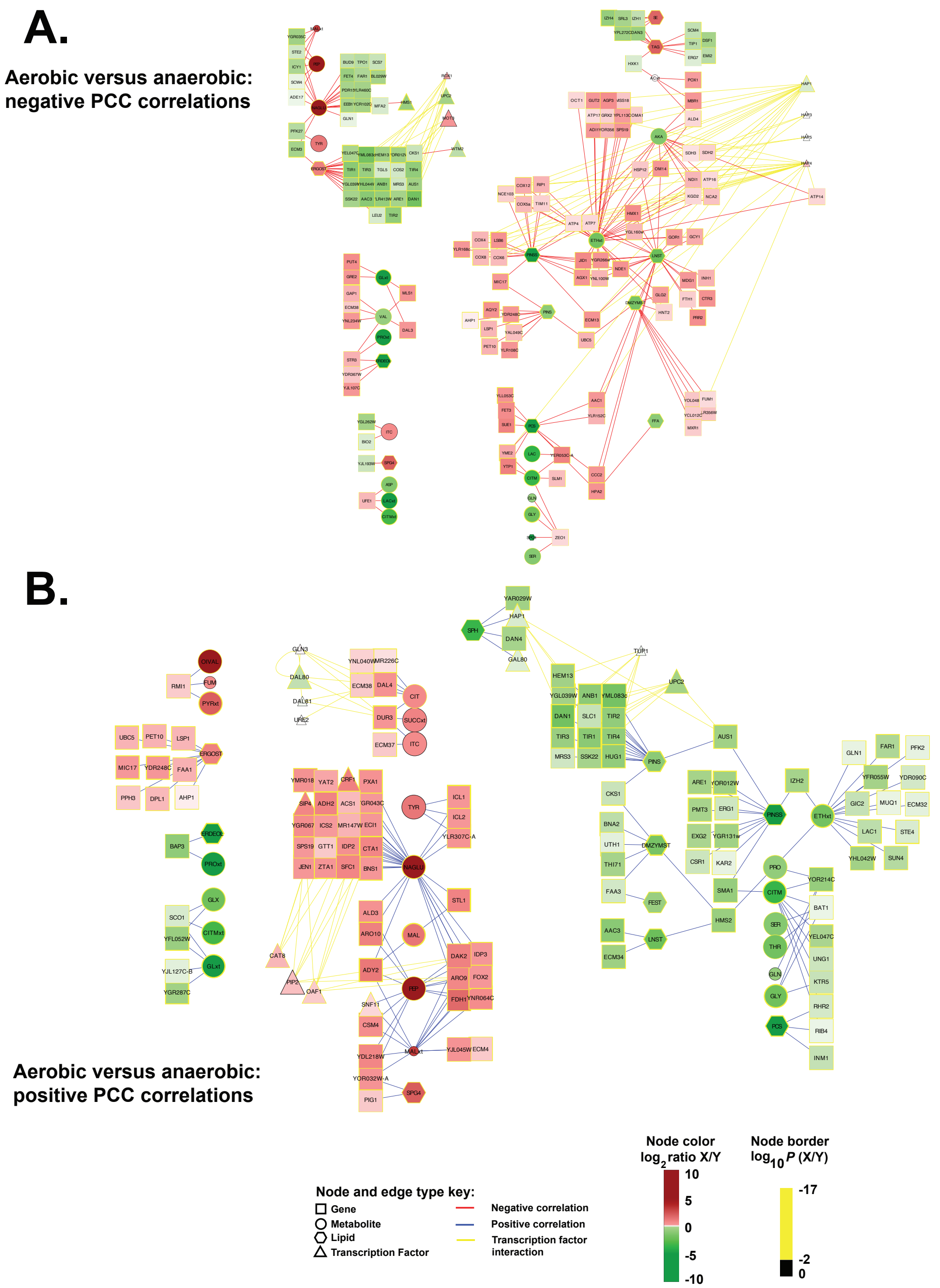

Figure S21 Correlation analysis demonstrates significant ($P \leq 0.001$ following Bonferroni correction) gene-lipid and gene-metabolite relationships when comparing aerobic "O" versus anaerobic "A" conditions. (A) Negative Pearson Correlation Coefficients (PCC). (B) Positive Pearson Correlation Coefficients (PCC). Enriched transcription factors are shown (yellow edges). Measurement ratios were visualized with a $\log_2$ color-bar and the color of each node border represents the $\log_{10}$(p-value) (see node and edge color key).

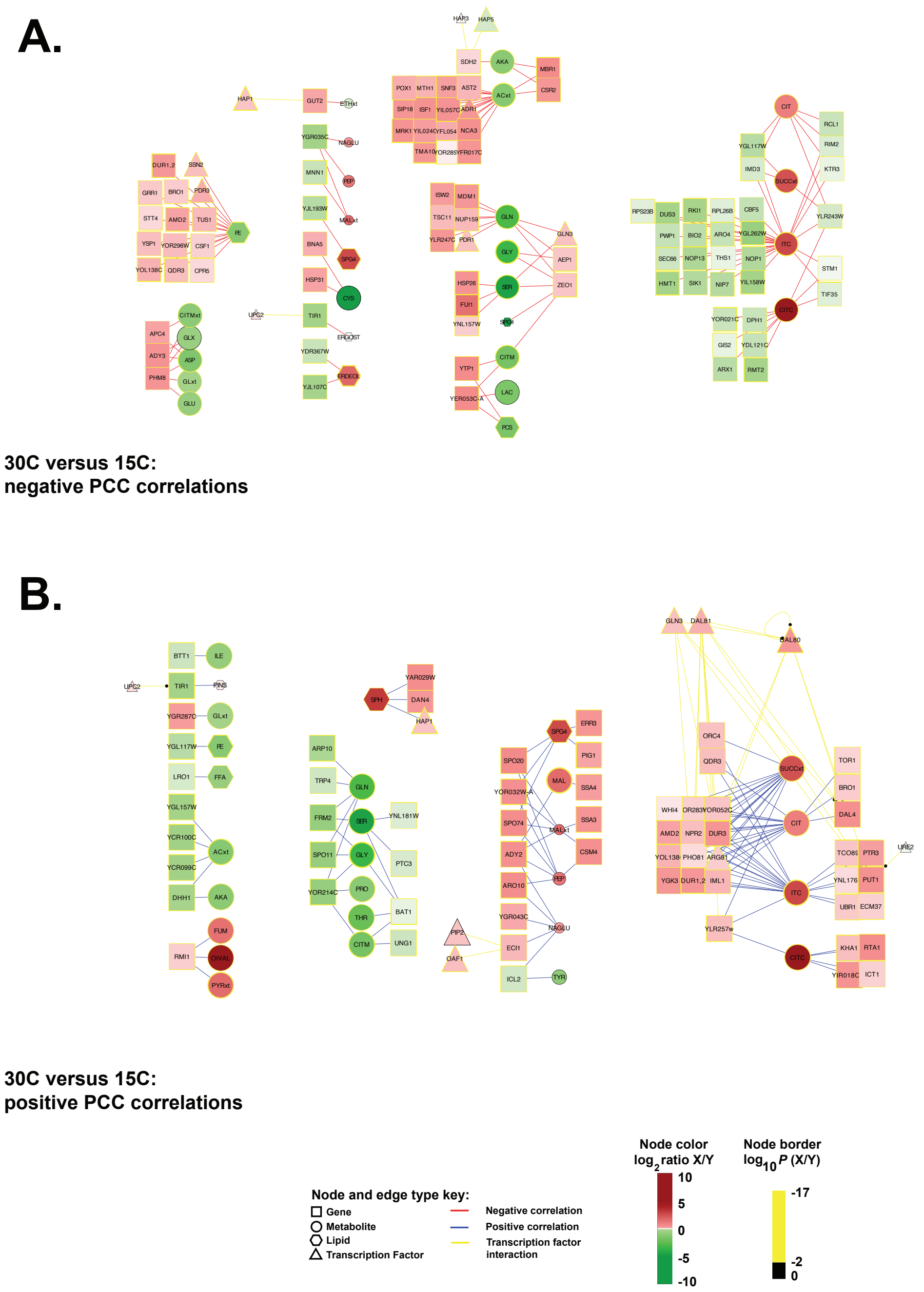

Figure S22 Correlation analysis demonstrates significant ($P \leq 0.001$ following Bonferroni correction) gene-lipid and gene-metabolite relationships when comparing high temperature (30°C) versus low temperature (15°C) conditions. (A) Negative Pearson Correlation Coefficients (PCC). (B) Positive Pearson Correlation Coefficients (PCC). Enriched transcription factors are shown (yellow edges). Measurement ratios were visualized with a $\log_2$ color-bar and the color of each node border represents the $\log_{10}$(p-value) (see node and edge color key).

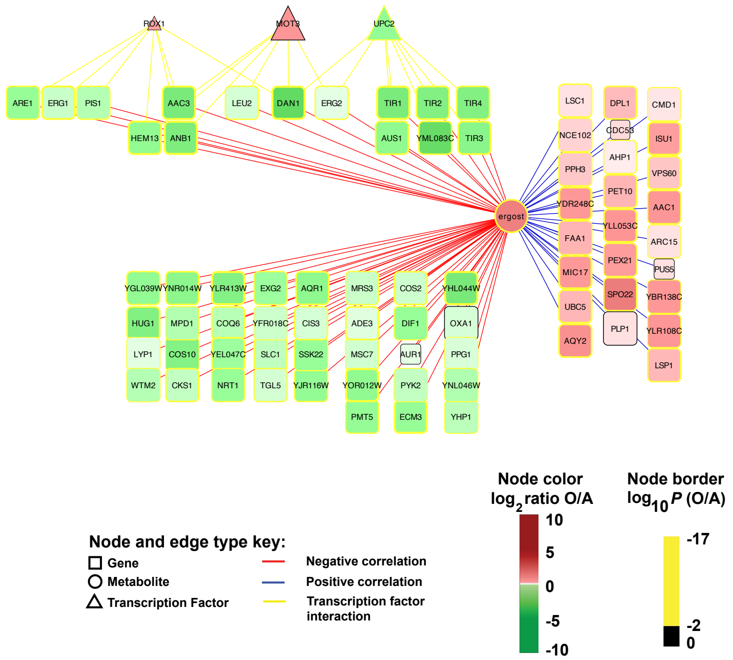

Figure S23 Correlation analysis demonstrates significant gene-ergosterol relationships ($P \leq 0.01$ following Bonferroni correction). As indicated in **Table 1**, the total number of interactions is 76 (all are shown). Measurement ratios for aerobic versus anaerobic conditions were visualized with a $\log_2$ color-bar and the color of each node border represents the $\log_{10}$(p-value) (see node and edge color key). Transcription factors identified in the enrichment analysis are shown (see node and edge key).

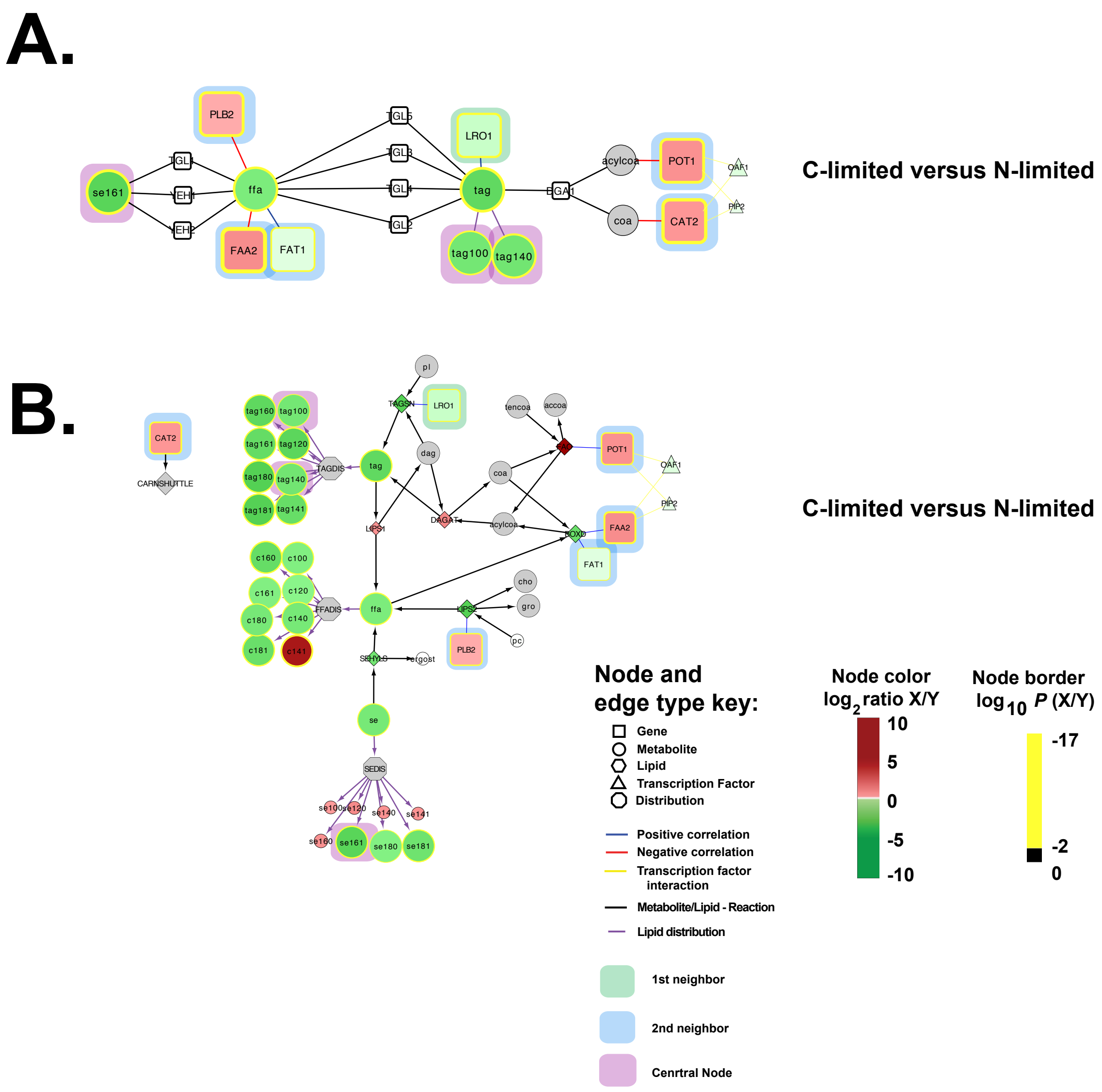

Figure S24 Integrative method for correlation of omics data reveals global regulatory signatures. Correlation networks for steryl ester 16:1 (se161), triacylglycerol 10:0 (tag100), and triacylglycerol 14:0 (tag140) show 1st (green highlight) and 2nd (blue highlight) significantly linked genes under aerobic versus anaerobic conditions. In (A), genes in small white boxes were not identified as significantly correlated to se161, tag100, and tag140, but are represented as "connector nodes" between metabolites. TFs implicated by the enrichment analysis are shown. The co-regulated gene neighborhood network from (A) was expanded to include genes and metabolites necessary to carry out the metabolic transformations indicated (B). This provides a more integrated perspective of cellular regulation. Measurement ratios were visualized with a $\log_2$ color-bar and the color of each node border represents the $\log_{10}$(p-value) (see node and edge color key). Gray coloring indicates the lack of a measurement for that node.

Literature Cited:

Gombert AK, Moreira dos Santos M, Christensen B, Nielsen J (2001) Network identification and flux quantification in the central metabolism of Saccharomyces cerevisiae under different conditions of glucose repression. *Journal of Bacteriology* **183:** 1441-1451.

Nookaew I, Jewett MC, Meechai A, Thammarongtham C, Laoteng K, Cheevadhanarak S, Nielsen J, Bhumiratana S (2008) The genome-scale metabolic model iIN800 of Saccharomyces cerevisiae and its validation: a scaffold to query lipid metabolism. BMC systems biology 2: 71.

Tables S1-S2
Available for download at http://www.g3journal.org/lookup/suppl/doi:10.1534/g3.113.006601/-/DC1

Table S1 Metabolic reconstruction of *iIN800*

Table S2 Normalized mRNA, metabolite, and lipid data

Table S3 Physiological yield data and steady-state nutrient concentrations in chemostat cultures. See below, and download the Excel file at http://www.g3journal.org/lookup/suppl/doi:10.1534/g3.113.006601/-/DC1.

Nutrient Limitation	Oxygen Condition	T [ºC]	Biomass (g/L)	D (hr-1)	Residual Glucose (g/L)	Yxs (Cmol/Cmol)	Yxn (Cmol/Nmol)
Carbon	Anaerobic	30	1.18 ± 0.06	0.047 ± 0.002	b.d.	0.14 ± 0.003	n.d.
Carbon	Anaerobic	15	1.14 ± 0.13	0.051 ± 0.003	b.d.	0.14 ± 0.003	n.d.
Carbon	Aerobic	30	5.93 ± 0.53	0.052 ± 0.005	b.d.	0.59 ± 0.031	n.d.
Carbon	Aerobic	15	6.84 ± 0.49	0.050 ± 0.003	b.d.	0.68 ± 0.032	n.d.
Nitrogen	Anaerobic	30	3.05 ± 0.20	0.050 ± 0.001	18.3 ± 0.8	0.07 ± 0.001	11.6 ± 0.4
Nitrogen	Anaerobic	15	1.80 ± 0.20	0.049 ± 0.001	16.0 ± 0.8	0.07 ± 0.011	8.3 ± 1.5
Nitrogen	Aerobic	30	3.84 ± 0.17	0.049 ± 0.000	17.9 ± 0.5	0.11 ± 0.003	15.8 ± 0.3
Nitrogen	Aerobic	15	2.72 ± 0.39	0.050 ± 0.002	17.0 ± 0.6	0.16 ± 0.014	10.4 ± 1.1

Nutrient Limitation	Oxygen Condition	T [ºC]	Yace/s (Cmol/Cmol)	Ysuc/s (Cmol/Cmol)	Yeth/s (Cmol/Cmol)	Ygly/s (Cmol/Cmol)	Ypyr/s (Cmol/Cmol)
Carbon	Anaerobic	30	0.000 ± 0.000	0.000 ± 0.000	0.53 ± 0.00	0.055 ± 0.002	0.00031 ± 0.00017
Carbon	Anaerobic	15	0.000 ± 0.000	0.000 ± 0.000	0.52 ± 0.00	0.069 ± 0.0052	0.00022 ± 5.5E-05
Carbon	Aerobic	30	0.000 ± 0.000	0.000 ± 0.000	0.00 ± 0.00	0.000 ± 0	0.00024 ± 1.3E-05
Carbon	Aerobic	15	0.000 ± 0.000	0.000 ± 0.000	0.00 ± 0.00	0.000 ± 0	0.00000 ± 0
Nitrogen	Anaerobic	30	0.003 ± 0.000	0.003 ± 0.001	0.62 ± 0.00	0.002 ± 4E-05	0.00080 ± 0.00036
Nitrogen	Anaerobic	15	0.006 ± 0.002	0.000 ± 0.000	0.56 ± 0.02	0.004 ± 0.0008	0.00071 ± 0.00037
Nitrogen	Aerobic	30	0.004 ± 0.000	0.009 ± 0.000	0.49 ± 0.00	0.001 ± 3E-06	0.00919 ± 0.00051
Nitrogen	Aerobic	15	0.015 ± 0.001	0.002 ± 0.000	0.43 ± 0.04	0.000 ± 0.0001	0.00329 ± 7.4E-05

Nutrient Limitation	Oxygen Condition	T [ºC]	Yco2/s (Cmol/Cmol)	Carbon Balance
Carbon	Anaerobic	30	0.20 ± 0.006	0.92 ± 0.007
Carbon	Anaerobic	15	0.19 ± 0.031	0.92 ± 0.032
Carbon	Aerobic	30	0.32 ± 0.048	0.90 ± 0.053
Carbon	Aerobic	15	0.33 ± 0.004	1.01 ± 0.031
Nitrogen	Anaerobic	30	0.28 ± 0.017	0.97 ± 0.017
Nitrogen	Anaerobic	15	0.31 ± 0.033	0.95 ± 0.042
Nitrogen	Aerobic	30	0.35 ± 0.019	0.96 ± 0.02
Nitrogen	Aerobic	15	0.35 ± 0.038	0.99 ± 0.025

b.d. - below detection, n.d. - not determined, ace - acetate, suc – succinate, eth – ethanol, gly – glycerol, pyr – pyruvate, co2 – carbon dioxide

Table S4 Multi-way ANOVA results for single factors: carbon-limited vs nitrogen-limited, "CN"; aerobic vs anaerobic, "OA"; and 30C vs 15C "Tt"; with associated log2-fold-change (LFC) and p-values. This supplemental file also contains data on interaction factors (between pairs of single factors): CN:OA; CN:Tt; OA:Tt; and the interaction between all factors, CN:OA:Tt. Table S4 is available for download as an Excel file at http://www.g3journal.org/lookup/suppl/doi:10.1534/g3.113.006601/-/DC1.

Table S5   Percent variance captured by each Principle Component (PC) dimension

PC	PC1	PC2	PC3
**mRNA**	36.7	23.6	15.2
**Metabolites**	62.9	19.3	8.2
**Lipids**	56.8	21.8	9.8

Tables S6 and S7 are available for download as Excel files at http://www.g3journal.org/lookup/suppl/doi:10.1534/g3.113.006601/-/DC1.

Table S6 Metabolic model for cytoscape visualization used in this study. This model centers the genome scale metabolic network *iIN800* on our metabolite and lipid measurements. As described in the manuscript, integrated analyses we performed used the complete *iIN800* model described by Nookaew *et al.* (2008). All reaction, metabolite, and lipid abbreviations are also described.

Table S7 *In silico* fluxes under the constraints of maximized biomass production, a steady state metabolic network, and fixed protein composition. Fluxes were normalized to the glucokinase reaction G6PS, which was set to 100. Each experiment is given a three letter code (C-limited, “C”; N-limited, “N”; aerobic, “O”; anaerobic, “A”; 30°C, “T”; and 15°C, “t”). For reaction abbreviations, see **Table S6**.

Table S8 Significant lipids, metabolites, and genes when comparing nitrogen-limited aerobic conditions (NOx = NOT & NOt) versus all other conditions (*i.e.*, COT, COt, CAT, CAt, NAT, & NAt) are shown. Significance was determined by $P \leq 0.01$ following Bonferroni correction.

Type	Label	log10pvalue	log2(NOx/others)
LIP	SE161	-13.0	3.8
LIP	TAG	-11.4	2.5
LIP	TAG181	-11.1	3.0
LIP	SE181	-9.8	3.0
LIP	TAG161	-9.0	3.1
LIP	SE	-8.8	2.7
LIP	TAG141	-5.8	1.9
LIP	TAG180	-5.4	2.7
LIP	TAG160	-4.8	1.9
LIP	SE180	-4.1	2.1
LIP	PSPH	-3.6	-1.6
MET	PYRxt	-3.5	3.6
MET	PYR	-3.1	2.7
MET	AKG	-2.5	2.5
GENE	SRL3	-8.6	-0.4
GENE	IZH4	-8.1	-1.9
GENE	TIP1	-7.4	-0.3
GENE	TSA2	-5.7	-0.7
GENE	HSP150	-5.7	-0.1
GENE	DAN3	-5.2	-1.3
GENE	IZH1	-4.6	-0.2
GENE	YPL272C	-4.4	-1.0
GENE	SCM4	-4.2	-0.2
GENE	PLB2	-3.8	-0.5
GENE	DAP1	-3.4	-0.2
GENE	GPX2	-3.2	0.2
GENE	IDS2	-3.2	0.1
GENE	YGR146C	-3.0	-0.6
GENE	YBL095W	-2.9	0.2
GENE	ERG6	-2.9	-0.1
GENE	ERG7	-2.6	-0.1
GENE	YDR352W	-2.6	0.2
GENE	YPL199C	-2.4	0.1
GENE	PTP3	-2.3	0.2
GENE	CRH1	-2.2	-0.1
GENE	YMR226C	-2.1	0.1
GENE	TEP1	-2.1	0.4
GENE	HES1	-2.1	-1.4
GENE	MID2	-2.0	-0.2
GENE	YEL070W	-2.0	-0.4

Table S9 Direct connections between genes and lipids or metabolites in the *iIN800* metabolic network and the correlation network.

Gene	Lip/Met	PCC
ARE1	Ergosterol	Neg
AUS1	Ergosterol	Neg
LSB6	Phosphatidylinositol di-substituted medium acyl-chain (PINSS)	Neg
LSB6	Phosphatidylinositol di-substituted medium acyl-chain 14:0 (PINSS140)	Neg
GAP1	Valine	Neg

Lip: lipid
Met: metabolite
PCC: Pearson correlation coefficient
Neg: negative correlation

Table S10 Based on measured lipids and metabolites that were identified in the correlation analysis, we observed that sterol levels were most highly correlated with 1st and 2nd gene neighbors (*P* ≤ 0.01, Benjamini Hochberg *p*-value adjustment). Whereas ~63% of sterols measured were highly correlated to 1st and 2nd gene neighbors, only ~17% of amino acids were. These data suggest that sterol biosynthesis is more regulated at the transcriptional level than amino acid biosynthesis. Within the phospholipid category, we note that 3 of 9 (or 33%) major phosphatidylinositol species were highly correlated to 1st and 2nd gene neighbors (*PINS*, PINS100, PINS120, PINS140, PINS141, *PINS160*, PINS161, PINS180, *PINS181*; significant species in *italics* and underlined).

	Total species	Significant *P* ≤ 0.01
sterol	***8***	***5***
organic acid	20	5
currency metabolite	2	2
phospholipid	53	4
neutral lipid	16	3
amino acids	***18***	***3***
sphingolipid	2	0
fatty acid	8	0
alcohols	2	0

Total metabolite and lipid species (first column):
sterol: ERGOST, EPST, LNST, DMZYMST, ZYMST, ERG722OST, ERTEOL, FEST
organic acid: PEP, MAL, SUCC, PYRxt, PYR, ACxt, GABA, MALxt, AKG, ORN, FUM, LACxt, ICIT, NAGLUm, GLX, OIVAL, CIT, ITCm, LAC, IPPMAL
currency metabolite: NADPH, NADP
phospholipid: PC, PC100, PC120, PC140, PC141, PC160, PC161, PC180, PC181, PCS, PCS100, PCS120, PCS140, PCS141, PCS160, PCS161, PCS180, PCS181, PE, PE100, PE120, PE140, PE141, PE160, PE161, PE180, PE181, PINS, PINS100, PINS120, PINS140, PINS141, PINS160, PINS161, PINS180, PINS181, PINSS, PINSS100, PINSS120, PINSS140, PINSS141, PINSS160, PINSS161, PINSS180, PINSS181, PS, PS100, PS120, PS140, PS160, PS161, PS180, PS181
neutral lipid: TAG, TAG140, TAG100, SE161, TAG160, TAG120, TAG180, SE, SE181, TAG181,TAG141, SE180, TAG161, SE141, SE140, SE120, SE160, SE100
amino acids: LYS, TYR, PROxt, ALA, HIS, ASP, CYS, VAL, PHE, THR, ILE, GLU, ASN, PRO, GLY, SER, LEU, GLN
sphingolipid: PSPH, SPH
fatty acid: FFA, C10, C12, C18, C14, C161, C141, C181, C16
alcohols: ETHxt, GLxt

Significant metabolites and lipids (second column):
Amino acids: alanine, proline (extracellular), lysine
Currency metabolites: NADPH, NADP
Neutral lipids: SE161, TAG100, TAG140
Organic acids: phosphoenolpyruvate, succinate, malate, pyruvate (extracellular)
Phospholipids: PINS181, PINS160, PINSS181, PINS
Sterols: ergosterol, lanosterol, episterol, 4,4-dimethylzymosterol, zymosterol

For metabolite and lipid abbreviations, see Table S6.

Table S11 KEGG pathways whose gene neighbors for sets of metabolites have a bias to be significantly correlated or anti-correlated. "KEGG:" KEGG pathway; "p.BH:" $P \leq 0.01$, Benjamini Hochberg *p*-value adjustment; "Genes in pathway:" the total number of genes in the defined KEGG pathway; "Genes in pathway and in CN:" the total number of genes in the defined KEGG pathway that are also in the correlation network (CN); "METS/LIPS in pathway:" the total number of metabolites and lipids in the defined KEGG pathway; "METS/LIPS in pathway and in CN:" the total number of metabolites and lipids in the defined KEGG pathway that are also in the correlation network.

KEGG	p.BH	Genes in Pathway	Genes in Pathway and in CN	METS/ LIPS in Pathway	METS/LIPS in Pathway and in CN
Pyrimidine metabolism	0	69	69	84	7
Aminophosphonate metabolism	0	8	8	39	28
Glycerophospholipid metabolism	0	23	23	100	56
Aminoacyl-tRNA biosynthesis	0	37	37	94	17
Glycine, serine and threonine metabolism	1.18E-10	43	42	108	27
One carbon pool by folate	2.89E-10	14	14	42	4
Phenylalanine, tyrosine and tryptophan biosynthesis	3.79E-09	20	19	54	11
Lysine biosynthesis	2.12E-07	15	15	42	7
Purine metabolism	3.47E-06	89	89	106	10
Sphingolipid metabolism	1.37E-05	13	13	19	5
Biosynthesis of steroids	0.000185	21	21	43	9
Alanine and aspartate metabolism	0.000324	34	31	66	10
Biosynthesis of phenylpropanoids	0.000727	35	33	65	8
Drug metabolism - other enzymes	0.000735	8	8	23	2
Valine, leucine and isoleucine biosynthesis	0.000739	18	18	60	10
Porphyrin and chlorophyll metabolism	0.000815	15	15	33	1
Nitrogen metabolism	0.00127	15	12	29	10
Selenoamino acid metabolism	0.00127	19	19	42	6
Histidine metabolism	0.00149	17	17	37	6
Sulfur metabolism	0.00253	12	12	40	5
Glutamate metabolism	0.0046	29	29	60	13
Methionine metabolism	0.00576	17	17	40	4
Glycolysis / Gluconeogenesis	0.00907	47	45	61	5
