# Supplementary material for: Mapping Condition-Dependent Regulation of Lipid Metabolism in Saccharomyces cerevisiae
Source: G3 (Bethesda). 2013 Nov 1;3(11):1979–95. doi: 10.1534/g3.113.006601 (PMC3815060; doi:10.1534/g3.113.006601)
Supplement: Supporting Information [file supp_g3.113.006601_FigureS1.pdf]

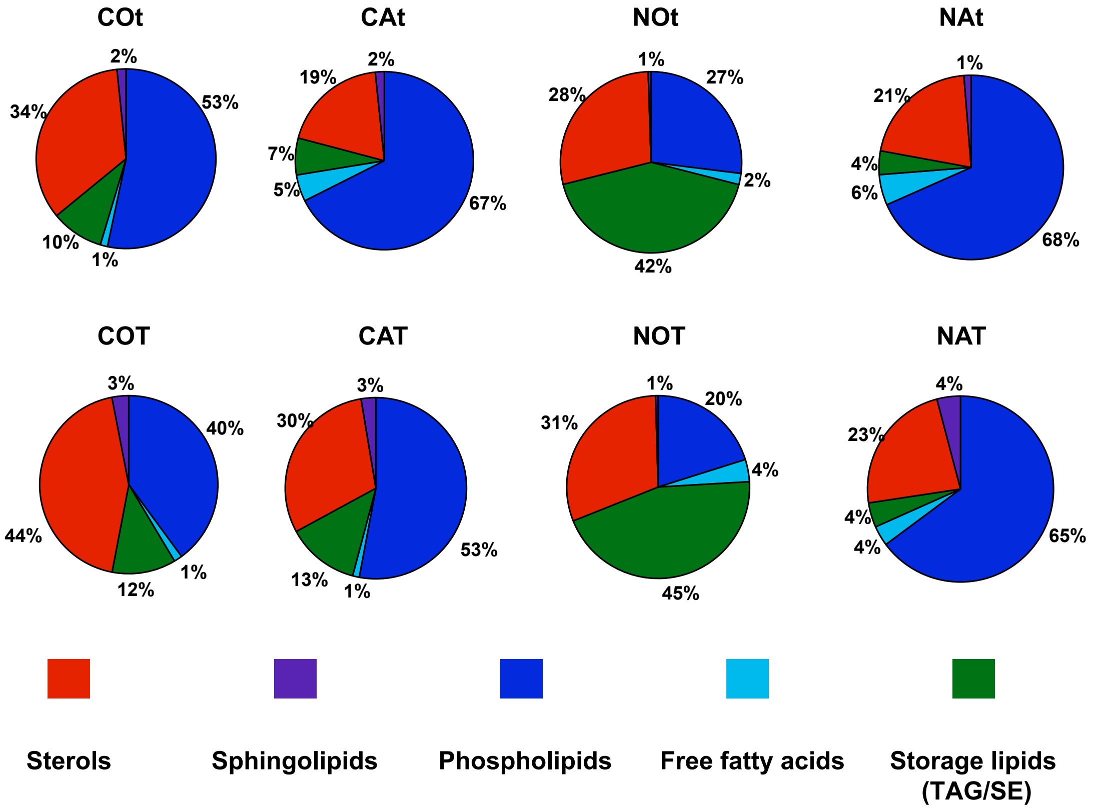

Figure S1 Pie chart representation of measured lipid classes for each experimental condition based on μmol/gDCW (DCW = dry cell weight). Each experiment is given a three letter code (C-limited, "C"; N-limited, "N"; aerobic, "O"; anaerobic, "A"; 30°C, "T"; and 15°C, "t"). For example, "COt" stands for C-limited, aerobic, and 15°C. These data highlight major changes across different conditions. In one example, nitrogen-limited aerobic conditions (NOt & NOT) have the largest percentage of storage lipids in the total lipid pool (42-45%). TAG: triacylglycerol, SE: steryl ester.
