# Supplementary material for: Mapping Condition-Dependent Regulation of Lipid Metabolism in Saccharomyces cerevisiae
Source: G3 (Bethesda). 2013 Nov 1;3(11):1979–95. doi: 10.1534/g3.113.006601 (PMC3815060; doi:10.1534/g3.113.006601)
Supplement: Supporting Information [file supp_g3.113.006601_FigureS5.pdf]

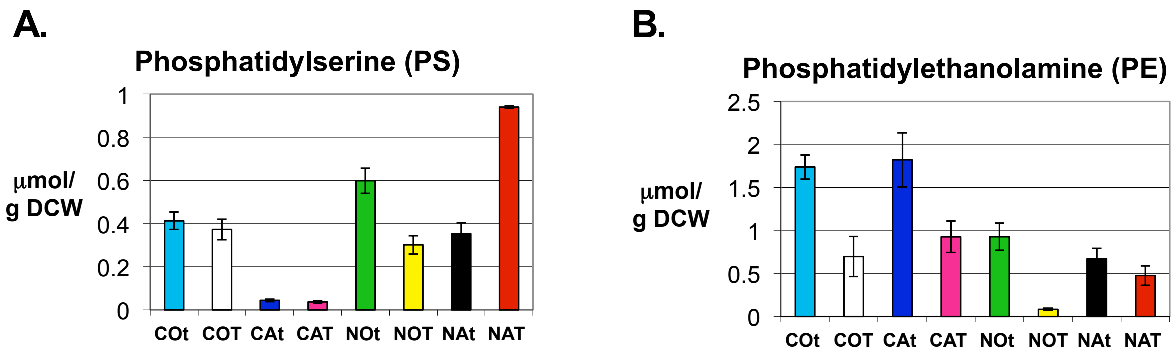

Figure S5 Phosphatidylserine (A) and phosphatidylethanolamine (B) content for each experimental condition based on μmol/gDCW (dry cell weight). Each experiment is given a three letter code (C-limited, "C"; N-limited, "N"; aerobic, "O"; anaerobic, "A"; 30°C, "T"; and 15°C, "t"). Phosphatidylethanolamine increases at lower temperatures.
