# Supplementary material for: Mapping Condition-Dependent Regulation of Lipid Metabolism in Saccharomyces cerevisiae
Source: G3 (Bethesda). 2013 Nov 1;3(11):1979–95. doi: 10.1534/g3.113.006601 (PMC3815060; doi:10.1534/g3.113.006601)
Supplement: Supporting Information [file supp_g3.113.006601_FigureS6.pdf]

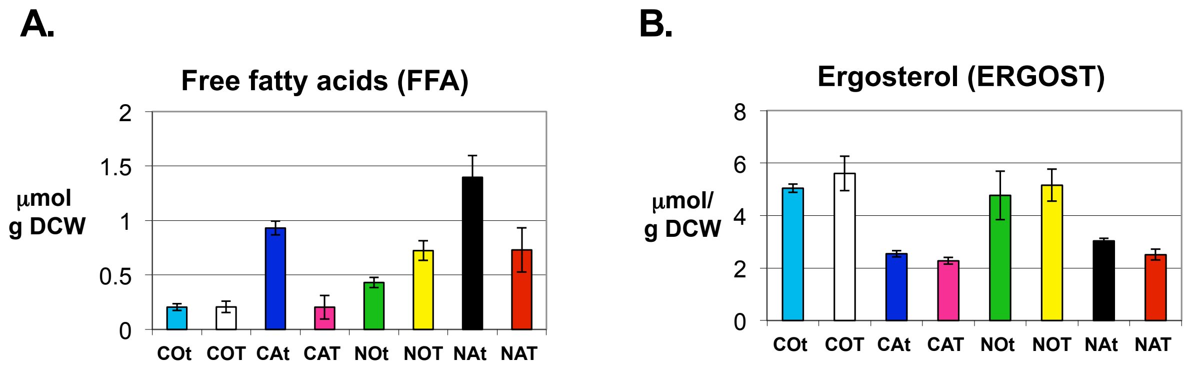

Figure S6 Free fatty acids (A) and ergosterol (B) content for each experimental condition based on μmol/gDCW (dry cell weight). Each experiment is given a three letter code (C-limited, "C"; N-limited, "N"; aerobic, "O"; anaerobic, "A"; 30°C, "T"; and 15°C, "t"). The concentration of free fatty acid is increased under anaerobic conditions, low temperature. Ergosterol levels are significantly higher under aerobic conditions, as expected since ergosterol biosynthesis is not active without oxygen. Note that anaerobic ergosterol levels represent amounts absorbed from the medium.
