# Supplementary material for: Mapping Condition-Dependent Regulation of Lipid Metabolism in Saccharomyces cerevisiae
Source: G3 (Bethesda). 2013 Nov 1;3(11):1979–95. doi: 10.1534/g3.113.006601 (PMC3815060; doi:10.1534/g3.113.006601)
Supplement: Supporting Information [file supp_g3.113.006601_FigureS9.pdf]

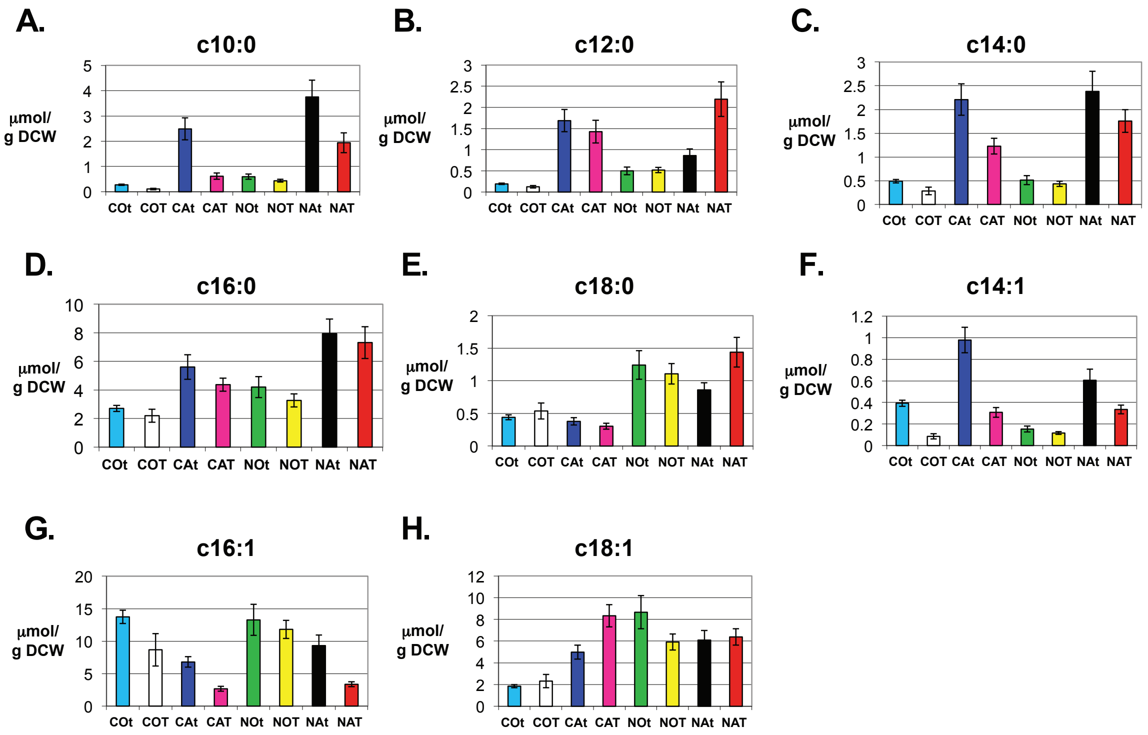

Figure S9 Total acyl chain composition from all measured lipid species (phosphatidylinositol, phosphatidylcholine, phosphatidylserine, phosphatidylethanolamine, triacylglycerol, steryl esters, and free fatty acids) for each experimental condition based on μmol/gDCW (dry cell weight). Each experiment is given a three letter code (C-limited, "C"; N-limited, "N"; aerobic, "O"; anaerobic, "A"; 30°C, "T"; and 15°C, "t"). The notation for acyl-chain length is given as c10:0, where the first number (in this case "10") is the number of carbon atoms and the second number (in this case "0") is the number of double bonds. Key observations include: medium chain acyl groups (C10:0, C12:0, and C14:0) are greater under anaerobic conditions, C16:0 is greater under anaerobic conditions, C18:0 is greater under nitrogen limitation, C14:1 and C16:1 are greater at lower temperatures where both a decreased chain length and the cis double bond will reduce the interaction between the acyl chains, and C18:1 is generally greater under nitrogen limitation.
