# Supplementary material for: Mapping Condition-Dependent Regulation of Lipid Metabolism in Saccharomyces cerevisiae
Source: G3 (Bethesda). 2013 Nov 1;3(11):1979–95. doi: 10.1534/g3.113.006601 (PMC3815060; doi:10.1534/g3.113.006601)
Supplement: Supporting Information [file supp_g3.113.006601_FigureS10.pdf]

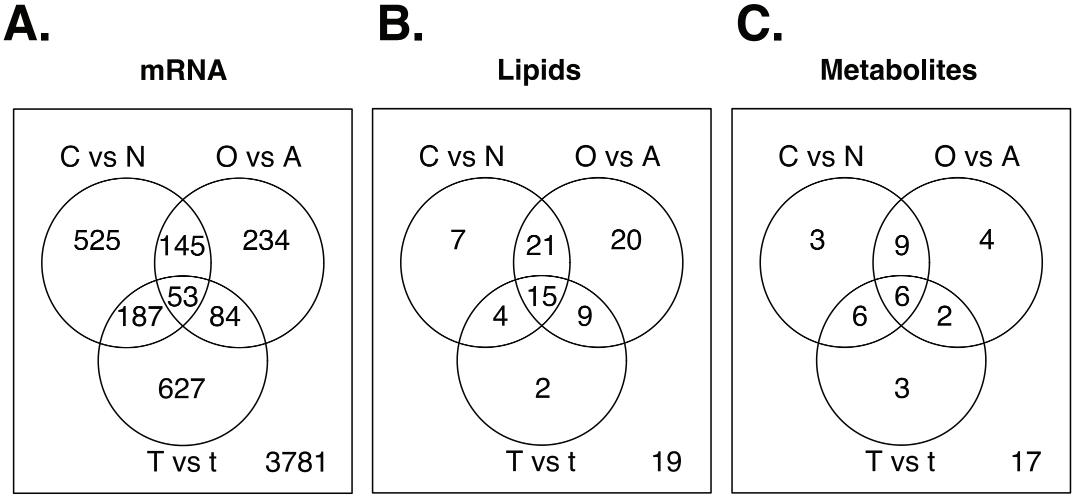

Figure S10 The overlap amongst significant genes (A), lipids (B), and metabolites (C) at a threshold of $P \leq 0.001$ following Bonferroni correction across experimental conditions. C-limited, "C"; N-limited, "N"; aerobic, "O"; anaerobic, "A"; 30°C, "T"; and 15°C, "t". See Table S4 for the list of genes from the Multi-way ANOVA.
