# Supplementary material for: Mapping Condition-Dependent Regulation of Lipid Metabolism in Saccharomyces cerevisiae
Source: G3 (Bethesda). 2013 Nov 1;3(11):1979–95. doi: 10.1534/g3.113.006601 (PMC3815060; doi:10.1534/g3.113.006601)
Supplement: Supporting Information [file supp_g3.113.006601_FigureS11.pdf]

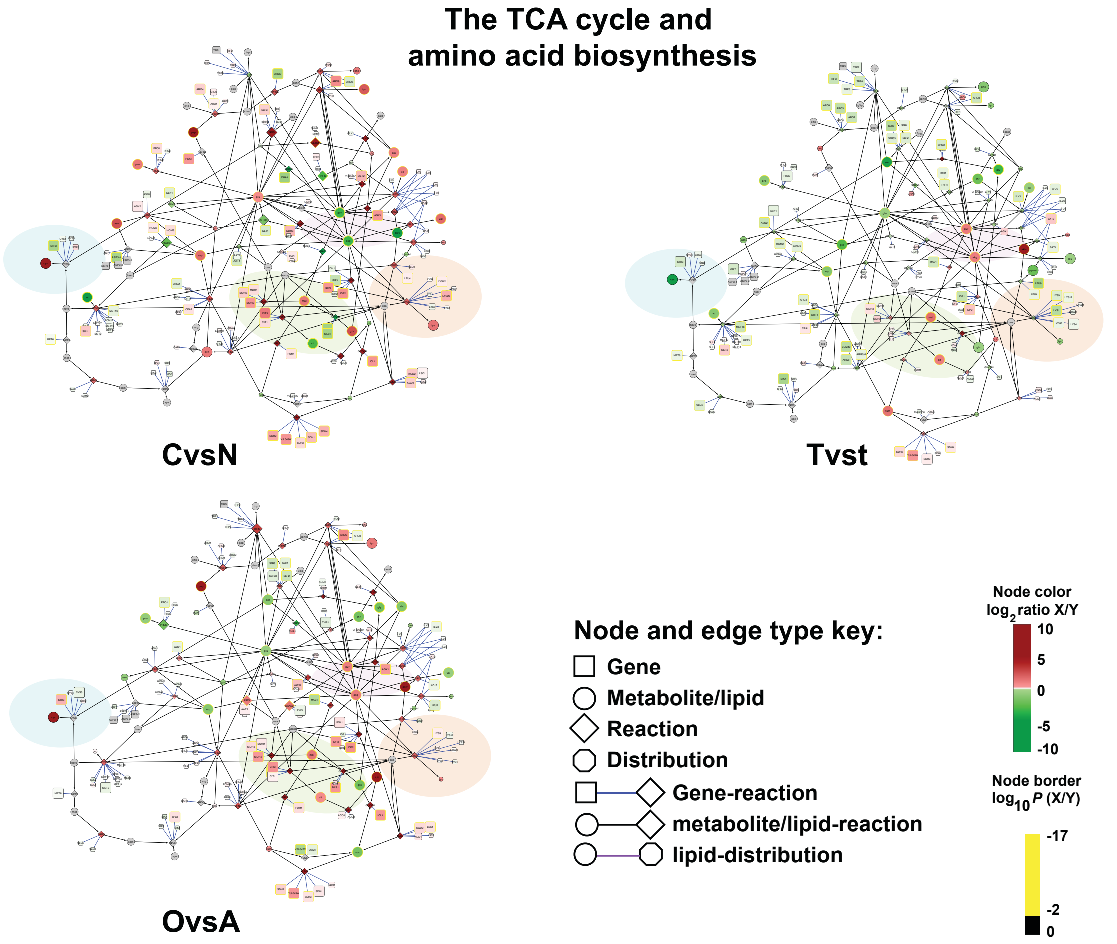

Figure S11 The condition dependent response of the TCA cycle and amino acid biosynthesis comparing C-Limited versus N-limited, 30°C versus 15°C, and aerobic versus anaerobic conditions. The areas highlighted show different up- and down-regulation patterns illustrating how Cytoscape visualization enables the targeted and rapid identification of changes across the cell. C-limited, “C”; N-limited, “N”; aerobic, “O”; anaerobic, “A”; 30°C, “T”; and 15°C, “t”. Measurement ratios were visualized with a $\log_2$ color-bar and the color of each node border represents the $\log_{10}$(*p*-value) (see node and edge color key). Gray coloring indicates the lack of a measurement for that node.
