# Supplementary material for: Mapping Condition-Dependent Regulation of Lipid Metabolism in Saccharomyces cerevisiae
Source: G3 (Bethesda). 2013 Nov 1;3(11):1979–95. doi: 10.1534/g3.113.006601 (PMC3815060; doi:10.1534/g3.113.006601)
Supplement: Supporting Information [file supp_g3.113.006601_FigureS13.pdf]

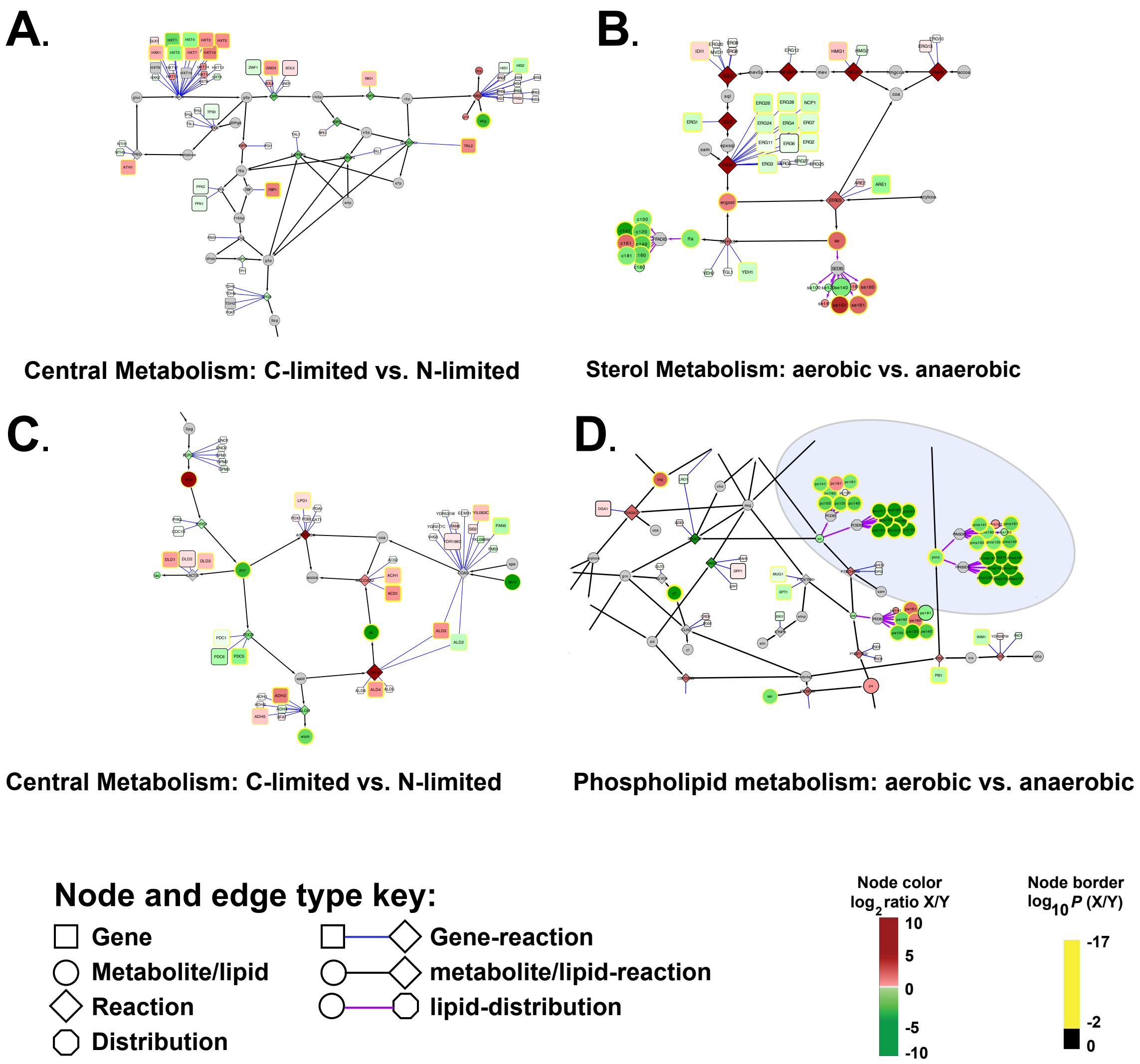
A.
Central Metabolism: C-limited vs. N-limited
B.
Sterol Metabolism: aerobic vs. anaerobic
C.
Central Metabolism: C-limited vs. N-limited
D.
Phospholipid metabolism: aerobic vs. anaerobic
Node and edge type key:
Gene
Metabolite/lipid
Reaction
Distribution
Gene-reaction
metabolite/lipid-reaction
lipid-distribution
Node color
log2 ratio X/Y
10
5
0
-5
-10
Node border
log10 P (X/Y)
-17
-2
0

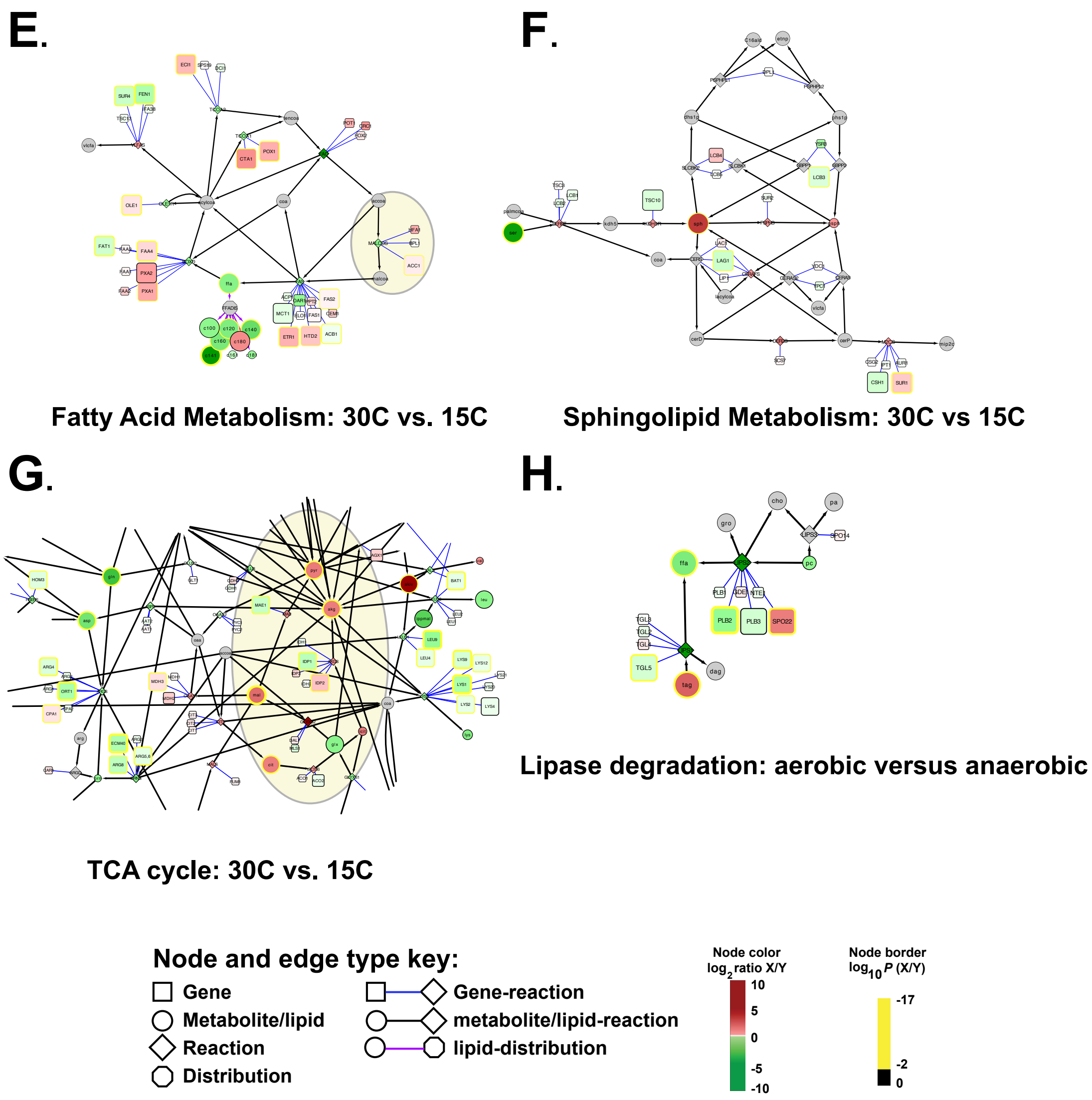

Figure S13 The condition dependent response of small cellular networks as visualized using Cytoscape. (A) Central metabolism: glycolysis, CvsN. (B) Sterol metabolism: OvsA. (C) Central metabolism: pyruvate metabolism, CvsN. (D) Phospholipid metabolism: OvsA. (E) Fatty acid metabolism: Tvst. (F) Sphingolipid metabolism: Tvst. (G) Central metabolism: TCA cycle, Tvst. G. Lipase metabolism: OvsA. C-limited, “C”; N-limited, “N”; aerobic, “O”; anaerobic, “A”; 30°C, “T”; and 15°C, “t”. Measurement ratios were visualized with a $\log_2$ color-bar and the color of each node border represents the $\log_{10}$(p-value) (see node and edge color key). Gray coloring indicates the lack of a measurement for that node.
