# Supplementary material for: Mapping Condition-Dependent Regulation of Lipid Metabolism in Saccharomyces cerevisiae
Source: G3 (Bethesda). 2013 Nov 1;3(11):1979–95. doi: 10.1534/g3.113.006601 (PMC3815060; doi:10.1534/g3.113.006601)
Supplement: Supporting Information [file supp_g3.113.006601_FigureS14.pdf]

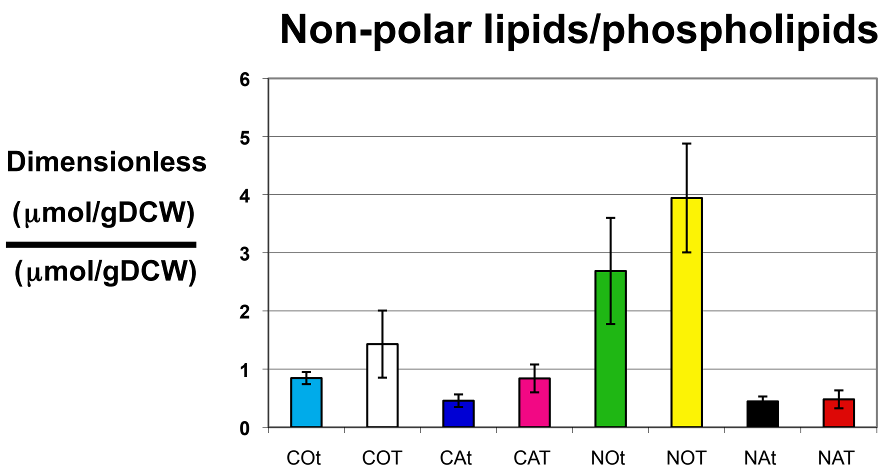

Figure S14 The ratio of non-polar lipids (free fatty acids, sterols, and steryl esters) to phospholipids (phosphatidylinositol, phosphatidylcholine, phosphatidylserine, and phosphatidylethanolamine) for each experimental condition based on μmol/gDCW (dry cell weight). Each experiment is given a three letter code (C-limited, “C”; N-limited, “N”; aerobic, “O”; anaerobic, “A”; 30°C, “T”; and 15°C, “t”). There is a shift away from phospholipid synthesis under nitrogen-limited aerobic conditions (*i.e.,* higher ratio above).
