# Supplementary material for: Mapping Condition-Dependent Regulation of Lipid Metabolism in Saccharomyces cerevisiae
Source: G3 (Bethesda). 2013 Nov 1;3(11):1979–95. doi: 10.1534/g3.113.006601 (PMC3815060; doi:10.1534/g3.113.006601)
Supplement: Supporting Information [file supp_g3.113.006601_FigureS16.pdf]

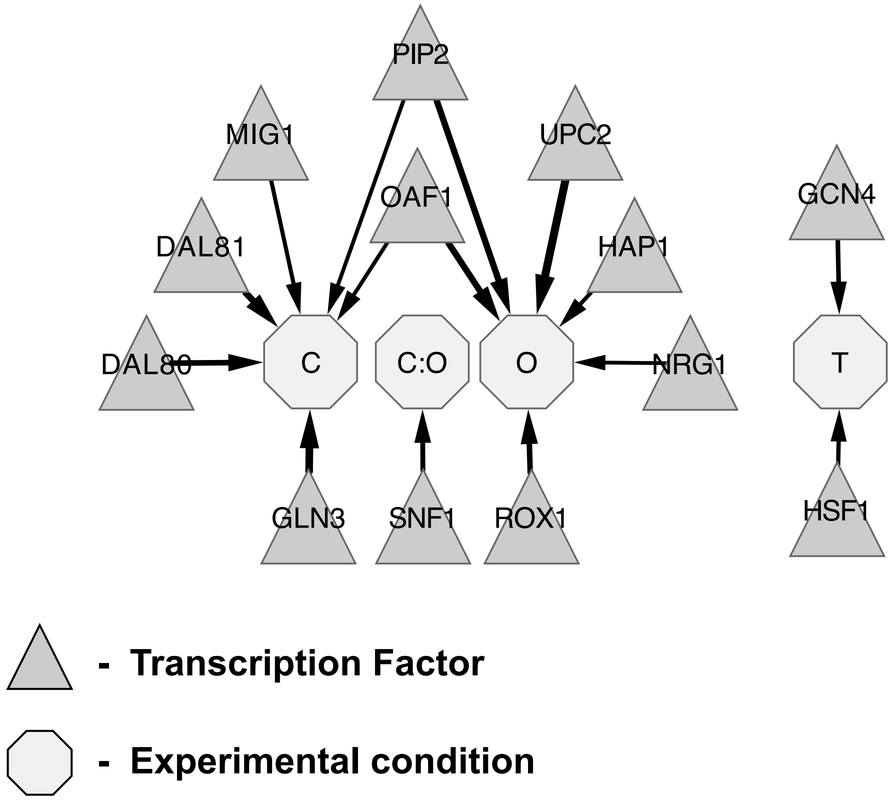

Figure S16 Network of translation factors enriched for different growth factors. Edge thickness represents the number of known transcription factor regulatory targets observed in the experimental condition (with thicker lines indicated more genes) (C-limited, “C”; N-limited, “N”; aerobic, “O”; anaerobic, “A”; 30°C, “T”; and 15°C, “t”).
