# Supplementary material for: Mapping Condition-Dependent Regulation of Lipid Metabolism in Saccharomyces cerevisiae
Source: G3 (Bethesda). 2013 Nov 1;3(11):1979–95. doi: 10.1534/g3.113.006601 (PMC3815060; doi:10.1534/g3.113.006601)
Supplement: Supporting Information [file supp_g3.113.006601_FigureS21.pdf]

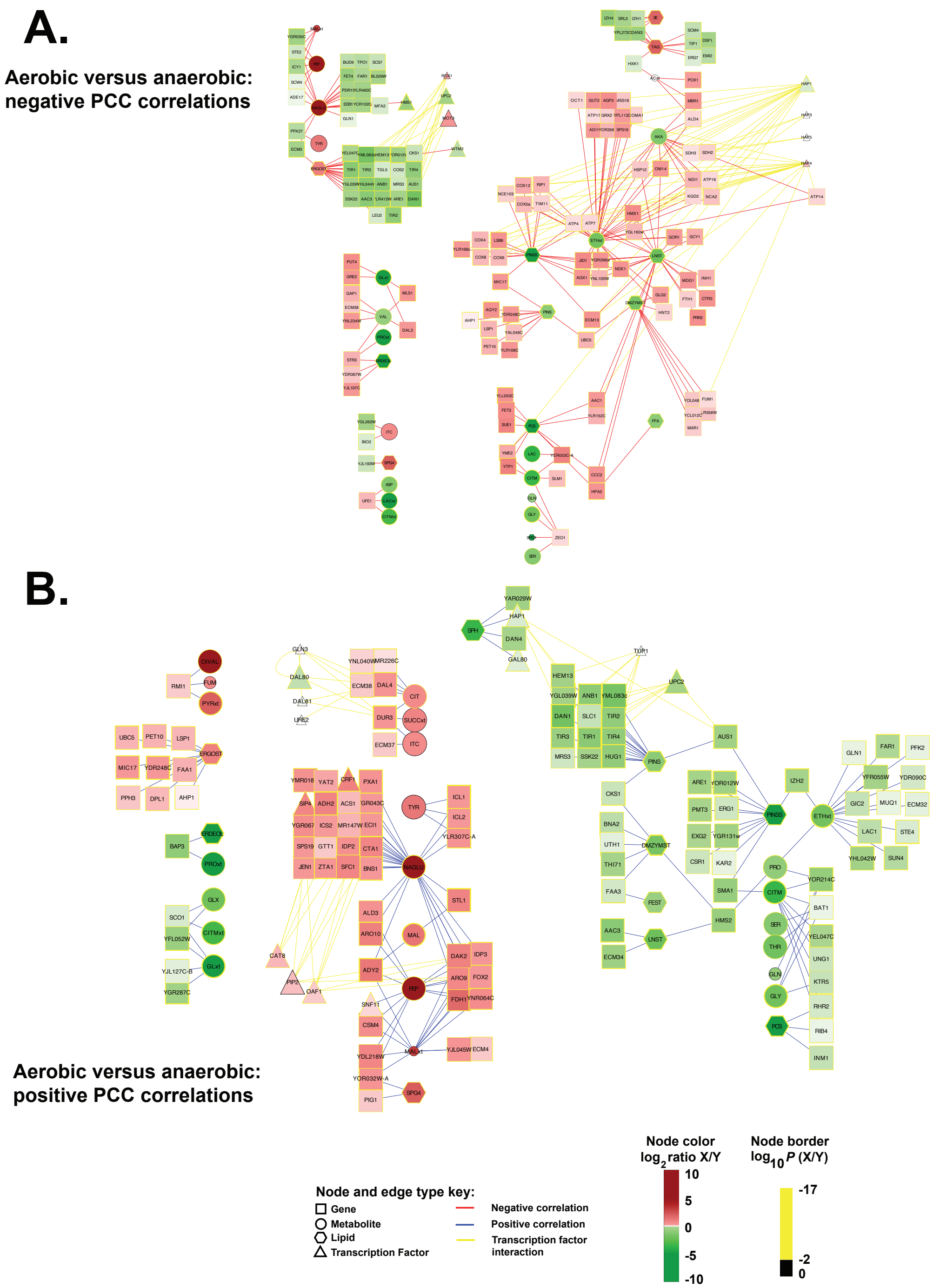

Figure S21 Correlation analysis demonstrates significant ($P \leq 0.001$ following Bonferroni correction) gene-lipid and gene-metabolite relationships when comparing aerobic "O" versus anaerobic "A" conditions. (A) Negative Pearson Correlation Coefficients (PCC). (B) Positive Pearson Correlation Coefficients (PCC). Enriched transcription factors are shown (yellow edges). Measurement ratios were visualized with a $\log_2$ color-bar and the color of each node border represents the $\log_{10}$(*p*-value) (see node and edge color key).
