# Supplementary material for: Mapping Condition-Dependent Regulation of Lipid Metabolism in Saccharomyces cerevisiae
Source: G3 (Bethesda). 2013 Nov 1;3(11):1979–95. doi: 10.1534/g3.113.006601 (PMC3815060; doi:10.1534/g3.113.006601)
Supplement: Supporting Information [file supp_g3.113.006601_FigureS22.pdf]

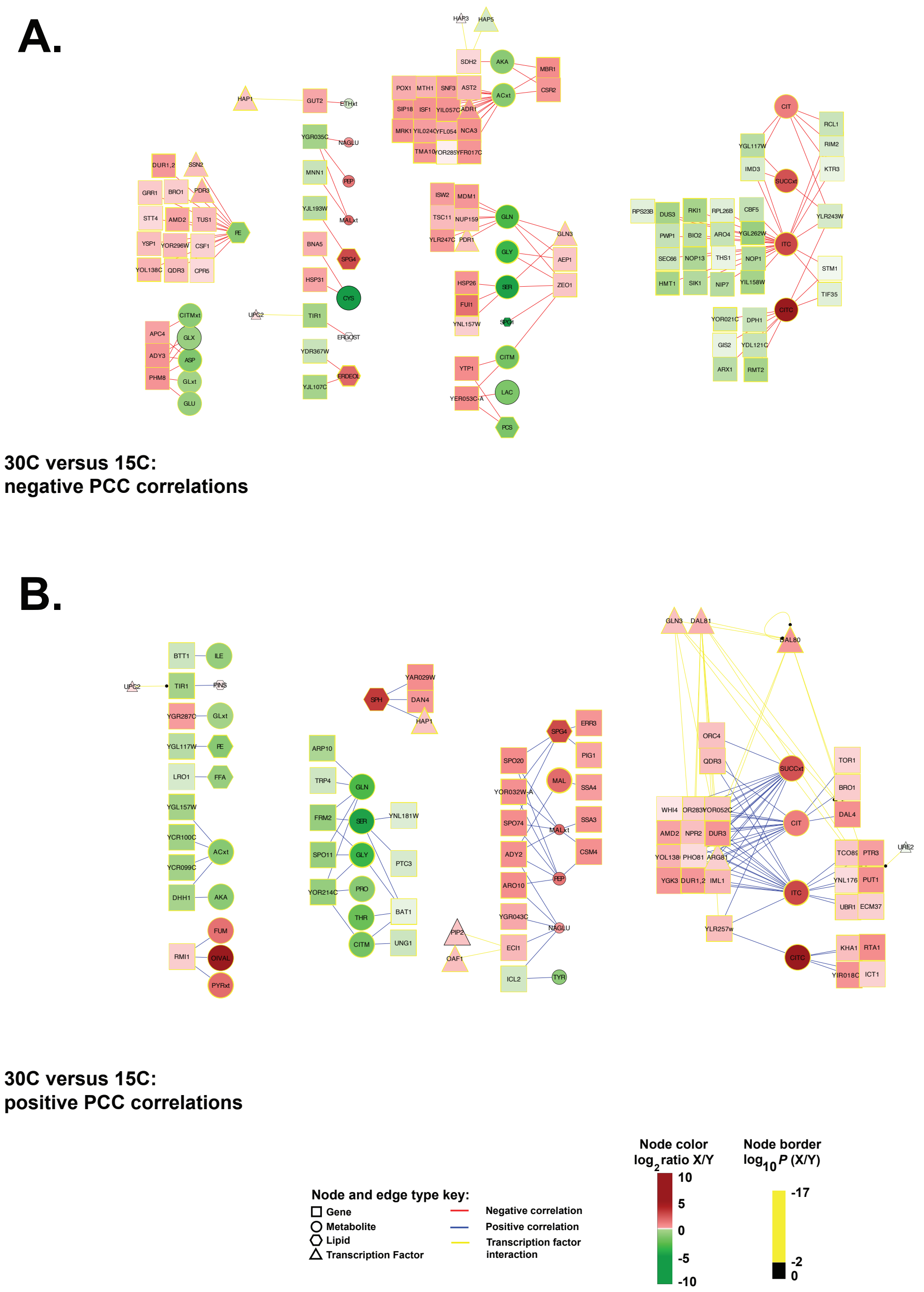

Figure S22 Correlation analysis demonstrates significant ($P \leq 0.001$ following Bonferroni correction) gene-lipid and gene-metabolite relationships when comparing high temperature (30°C) versus low temperature (15°C) conditions. (A) Negative Pearson Correlation Coefficients (PCC). (B) Positive Pearson Correlation Coefficients (PCC). Enriched transcription factors are shown (yellow edges). Measurement ratios were visualized with a log_2 color-bar and the color of each node border represents the log_{10}(*p*-value) (see node and edge color key).
