# Supplementary material for: Mapping Condition-Dependent Regulation of Lipid Metabolism in Saccharomyces cerevisiae
Source: G3 (Bethesda). 2013 Nov 1;3(11):1979–95. doi: 10.1534/g3.113.006601 (PMC3815060; doi:10.1534/g3.113.006601)
Supplement: Supporting Information [file supp_g3.113.006601_FigureS23.pdf]

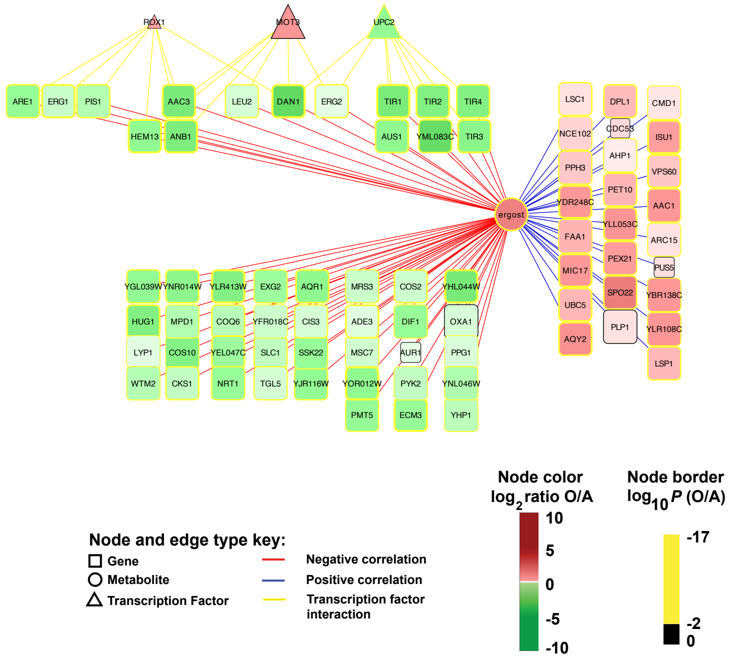

Figure S23 Correlation analysis demonstrates significant gene-ergosterol relationships ($P \leq 0.01$ following Bonferroni correction). As indicated in **Table 1**, the total number of interactions is 76 (all are shown). Measurement ratios for aerobic versus anaerobic conditions were visualized with a $\log_2$ color-bar and the color of each node border represents the $\log_{10}(p$-value) (see node and edge color key). Transcription factors identified in the enrichment analysis are shown (see node and edge key).
