# Supplementary material for: Mapping Condition-Dependent Regulation of Lipid Metabolism in Saccharomyces cerevisiae
Source: G3 (Bethesda). 2013 Nov 1;3(11):1979–95. doi: 10.1534/g3.113.006601 (PMC3815060; doi:10.1534/g3.113.006601)
Supplement: Supporting Information [file supp_g3.113.006601_FigureS24.pdf]

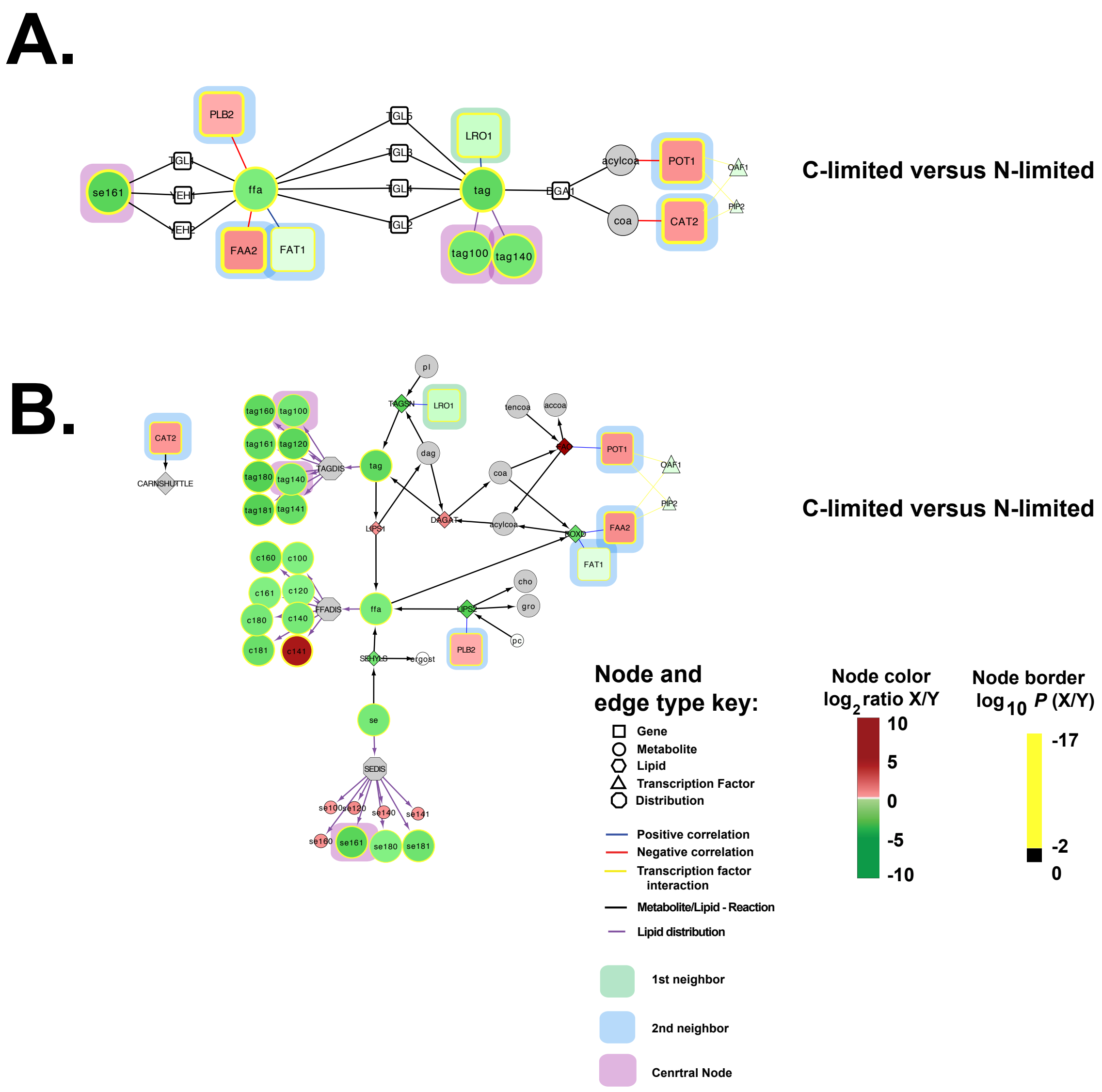

Figure S24 Integrative method for correlation of omics data reveals global regulatory signatures. Correlation networks for steryl ester 16:1 (se161), triacylglycerol 10:0 (tag100), and triacylglycerol 14:0 (tag140) show 1st (green highlight) and 2nd (blue highlight) significantly linked genes under aerobic versus anaerobic conditions. In (A), genes in small white boxes were not identified as significantly correlated to se161, tag100, and tag140, but are represented as "connector nodes" between metabolites. TFs implicated by the enrichment analysis are shown. The co-regulated gene neighborhood network from (A) was expanded to include genes and metabolites necessary to carry out the metabolic transformations indicated (B). This provides a more integrated perspective of cellular regulation. Measurement ratios were visualized with a $\log_2$ color-bar and the color of each node border represents the $\log_{10}$(*p*-value) (see node and edge color key). Gray coloring indicates the lack of a measurement for that node.
