# Supplementary material for: Mapping Condition-Dependent Regulation of Lipid Metabolism in Saccharomyces cerevisiae
Source: G3 (Bethesda). 2013 Nov 1;3(11):1979–95. doi: 10.1534/g3.113.006601 (PMC3815060; doi:10.1534/g3.113.006601)
Supplement: Supporting Information [file supp_g3.113.006601_TableS5.pdf]

Table S5   Percent variance captured by each Principle Component (PC) dimension

PC	PC1	PC2	PC3
**mRNA**	36.7	23.6	15.2
**Metabolites**	62.9	19.3	8.2
**Lipids**	56.8	21.8	9.8
