# Supplementary material for: Mapping Condition-Dependent Regulation of Lipid Metabolism in Saccharomyces cerevisiae
Source: G3 (Bethesda). 2013 Nov 1;3(11):1979–95. doi: 10.1534/g3.113.006601 (PMC3815060; doi:10.1534/g3.113.006601)
Supplement: Supporting Information [file supp_g3.113.006601_TableS8.pdf]

Table S8 Significant lipids, metabolites, and genes when comparing nitrogen-limited aerobic conditions (NOx = NOT & NOt) versus all other conditions (*i.e.*, COT, COt, CAT, CAt, NAT, & NAt) are shown. Significance was determined by $P \leq 0.01$ following Bonferroni correction.

Type	Label	log10pvalue	log2(NOx/others)
LIP	SE161	-13.0	3.8
LIP	TAG	-11.4	2.5
LIP	TAG181	-11.1	3.0
LIP	SE181	-9.8	3.0
LIP	TAG161	-9.0	3.1
LIP	SE	-8.8	2.7
LIP	TAG141	-5.8	1.9
LIP	TAG180	-5.4	2.7
LIP	TAG160	-4.8	1.9
LIP	SE180	-4.1	2.1
LIP	PSPH	-3.6	-1.6
MET	PYRxt	-3.5	3.6
MET	PYR	-3.1	2.7
MET	AKG	-2.5	2.5
GENE	SRL3	-8.6	-0.4
GENE	IZH4	-8.1	-1.9
GENE	TIP1	-7.4	-0.3
GENE	TSA2	-5.7	-0.7
GENE	HSP150	-5.7	-0.1
GENE	DAN3	-5.2	-1.3
GENE	IZH1	-4.6	-0.2
GENE	YPL272C	-4.4	-1.0
GENE	SCM4	-4.2	-0.2
GENE	PLB2	-3.8	-0.5
GENE	DAP1	-3.4	-0.2
GENE	GPX2	-3.2	0.2
GENE	IDS2	-3.2	0.1
GENE	YGR146C	-3.0	-0.6
GENE	YBL095W	-2.9	0.2
GENE	ERG6	-2.9	-0.1
GENE	ERG7	-2.6	-0.1
GENE	YDR352W	-2.6	0.2
GENE	YPL199C	-2.4	0.1
GENE	PTP3	-2.3	0.2
GENE	CRH1	-2.2	-0.1
GENE	YMR226C	-2.1	0.1
GENE	TEP1	-2.1	0.4
GENE	HES1	-2.1	-1.4
GENE	MID2	-2.0	-0.2
GENE	YEL070W	-2.0	-0.4
