# Supplementary material for: Mapping Condition-Dependent Regulation of Lipid Metabolism in Saccharomyces cerevisiae
Source: G3 (Bethesda). 2013 Nov 1;3(11):1979–95. doi: 10.1534/g3.113.006601 (PMC3815060; doi:10.1534/g3.113.006601)
Supplement: Supporting Information [file supp_g3.113.006601_TableS9.pdf]

Table S9 Direct connections between genes and lipids or metabolites in the *iIN800* metabolic network and the correlation network.

Gene	Lip/Met	PCC
ARE1	Ergosterol	Neg
AUS1	Ergosterol	Neg
LSB6	Phosphatidylinositol di-substituted medium acyl-chain (PINSS)	Neg
LSB6	Phosphatidylinositol di-substituted medium acyl-chain 14:0 (PINSS140)	Neg
GAP1	Valine	Neg

Lip: lipid
Met: metabolite
PCC: Pearson correlation coefficient
Neg: negative correlation
