# Supplementary material for: Mapping Condition-Dependent Regulation of Lipid Metabolism in Saccharomyces cerevisiae
Source: G3 (Bethesda). 2013 Nov 1;3(11):1979–95. doi: 10.1534/g3.113.006601 (PMC3815060; doi:10.1534/g3.113.006601)
Supplement: Supporting Information [file supp_g3.113.006601_TableS10.pdf]

Table S10 Based on measured lipids and metabolites that were identified in the correlation analysis, we observed that sterol levels were most highly correlated with 1st and 2nd gene neighbors ($P \leq 0.01$, Benjamini Hochberg *p*-value adjustment). Whereas ~63% of sterols measured were highly correlated to 1st and 2nd gene neighbors, only ~17% of amino acids were. These data suggest that sterol biosynthesis is more regulated at the transcriptional level than amino acid biosynthesis. Within the phospholipid category, we note that 3 of 9 (or 33%) major phosphatidylinositol species were highly correlated to 1st and 2nd gene neighbors (*PINS*, PINS100, PINS120, PINS140, PINS141, *PINS160*, PINS161, PINS180, *PINS181*; significant species in *italics* and underlined).

	Total species	Significant $P \leq 0.01$
sterol	***8***	***5***
organic acid	20	5
currency metabolite	2	2
phospholipid	53	4
neutral lipid	16	3
amino acids	***18***	***3***
sphingolipid	2	0
fatty acid	8	0
alcohols	2	0

Total metabolite and lipid species (first column):
sterol: ERGOST, EPST, LNST, DMZYMST, ZYMST, ERG722OST, ERTEOL, FEST
organic acid: PEP, MAL, SUCC, PYRxt, PYR, ACxt, GABA, MALxt, AKG, ORN, FUM, LACxt, ICIT, NAGLUm, GLX, OIVAL, CIT, ITCm, LAC, IPPMAL
currency metabolite: NADPH, NADP
phospholipid: PC, PC100, PC120, PC140, PC141, PC160, PC161, PC180, PC181, PCS, PCS100, PCS120, PCS140, PCS141, PCS160, PCS161, PCS180, PCS181, PE, PE100, PE120, PE140, PE141, PE160, PE161, PE180, PE181, PINS, PINS100, PINS120, PINS140, PINS141, PINS160, PINS161, PINS180, PINS181, PINSS, PINSS100, PINSS120, PINSS140, PINSS141, PINSS160, PINSS161, PINSS180, PINSS181, PS, PS100, PS120, PS140, PS160, PS161, PS180, PS181
neutral lipid: TAG, TAG140, TAG100, SE161, TAG160, TAG120, TAG180, SE, SE181, TAG181,TAG141, SE180, TAG161, SE141, SE140, SE120, SE160, SE100
amino acids: LYS, TYR, PROxt, ALA, HIS, ASP, CYS, VAL, PHE, THR, ILE, GLU, ASN, PRO, GLY, SER, LEU, GLN
sphingolipid: PSPH, SPH
fatty acid: FFA, C10, C12, C18, C14, C161, C141, C181, C16
alcohols: ETHxt, GLxt

Significant metabolites and lipids (second column):
Amino acids: alanine, proline (extracellular), lysine
Currency metabolites: NADPH, NADP
Neutral lipids: SE161, TAG100, TAG140
Organic acids: phosphoenolpyruvate, succinate, malate, pyruvate (extracellular)
Phospholipids: PINS181, PINS160, PINSS181, PINS
Sterols: ergosterol, lanosterol, episterol, 4,4-dimethylzymosterol, zymosterol

For metabolite and lipid abbreviations, see Table S6.
