# Supplementary material for: Mapping Condition-Dependent Regulation of Lipid Metabolism in Saccharomyces cerevisiae
Source: G3 (Bethesda). 2013 Nov 1;3(11):1979–95. doi: 10.1534/g3.113.006601 (PMC3815060; doi:10.1534/g3.113.006601)
Supplement: Supporting Information [file supp_g3.113.006601_TableS11.pdf]

Table S11 KEGG pathways whose gene neighbors for sets of metabolites have a bias to be significantly correlated or anti-correlated. "KEGG:" KEGG pathway; "p.BH:" $P \leq 0.01$, Benjamini Hochberg *p*-value adjustment; "Genes in pathway:" the total number of genes in the defined KEGG pathway; "Genes in pathway and in CN:" the total number of genes in the defined KEGG pathway that are also in the correlation network (CN); "METS/LIPS in pathway:" the total number of metabolites and lipids in the defined KEGG pathway; "METS/LIPS in pathway and in CN:" the total number of metabolites and lipids in the defined KEGG pathway that are also in the correlation network.

KEGG	p.BH	Genes in Pathway	Genes in Pathway and in CN	METS/ LIPS in Pathway	METS/LIPS in Pathway and in CN
Pyrimidine metabolism	0	69	69	84	7
Aminophosphonate metabolism	0	8	8	39	28
Glycerophospholipid metabolism	0	23	23	100	56
Aminoacyl-tRNA biosynthesis	0	37	37	94	17
Glycine, serine and threonine metabolism	1.18E-10	43	42	108	27
One carbon pool by folate	2.89E-10	14	14	42	4
Phenylalanine, tyrosine and tryptophan biosynthesis	3.79E-09	20	19	54	11
Lysine biosynthesis	2.12E-07	15	15	42	7
Purine metabolism	3.47E-06	89	89	106	10
Sphingolipid metabolism	1.37E-05	13	13	19	5
Biosynthesis of steroids	0.000185	21	21	43	9
Alanine and aspartate metabolism	0.000324	34	31	66	10
Biosynthesis of phenylpropanoids	0.000727	35	33	65	8
Drug metabolism - other enzymes	0.000735	8	8	23	2
Valine, leucine and isoleucine biosynthesis	0.000739	18	18	60	10
Porphyrin and chlorophyll metabolism	0.000815	15	15	33	1
Nitrogen metabolism	0.00127	15	12	29	10
Selenoamino acid metabolism	0.00127	19	19	42	6
Histidine metabolism	0.00149	17	17	37	6
Sulfur metabolism	0.00253	12	12	40	5
Glutamate metabolism	0.0046	29	29	60	13
Methionine metabolism	0.00576	17	17	40	4
Glycolysis / Gluconeogenesis	0.00907	47	45	61	5
