# Supplementary material for: Mapping Condition-Dependent Regulation of Lipid Metabolism in Saccharomyces cerevisiae
Source: G3 (Bethesda). 2013 Nov 1;3(11):1979–95. doi: 10.1534/g3.113.006601 (PMC3815060; doi:10.1534/g3.113.006601)
Supplement: Supporting Information [file supp_g3.113.006601_FigureS15.pdf]

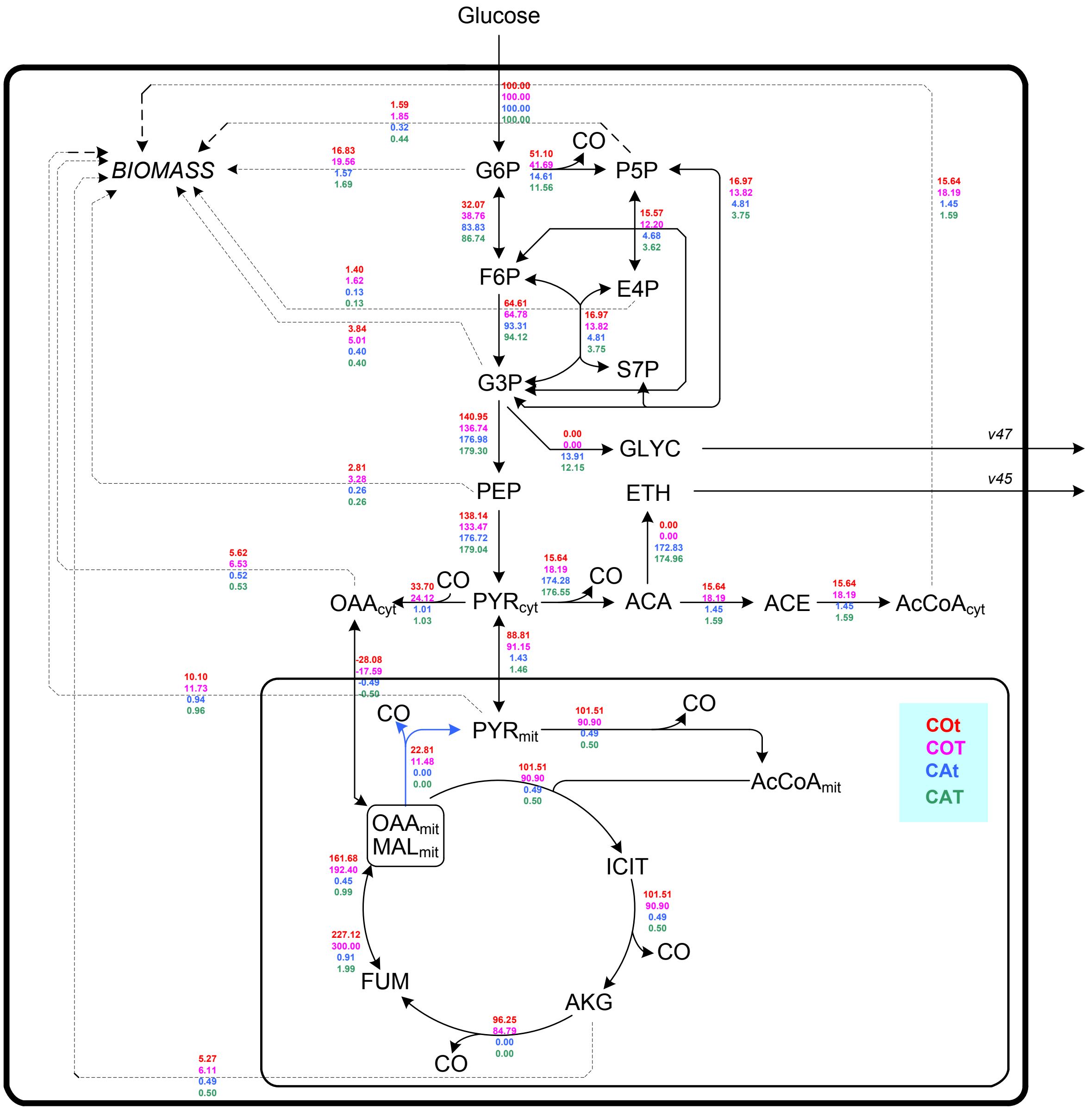

Figure S15 ^{13}C-flux analysis demonstrates carbon flux through the pentose phosphate pathway is significantly decreased under anaerobic conditions (CAt & CAT relative to COt & COT)**.** For example, flux from glucose-6-phosphate (G6P) to pentose-5-phosphate is approximately 3.5-fold higher in the aerobic case. All abbreviations are directly taken from Gombert et al., 2001. Carbon fluxes, measured here, are normalized to glucose uptake. Each experiment is given a three letter code (C-limited, "C"; N-limited, "N"; aerobic, "O"; anaerobic, "A"; 30°C, "T"; and 15°C, "t").
